# Connectomics Analysis Reveals First-, Second-, and Third-Order Thermosensory and Hygrosensory Neurons in the Adult *Drosophila* Brain

**DOI:** 10.1016/j.cub.2020.06.028

**Published:** 2020-08-17

**Authors:** Elizabeth C. Marin, Laurin Büld, Maria Theiss, Tatevik Sarkissian, Ruairí J.V. Roberts, Robert Turnbull, Imaan F.M. Tamimi, Markus W. Pleijzier, Willem J. Laursen, Nik Drummond, Philipp Schlegel, Alexander S. Bates, Feng Li, Matthias Landgraf, Marta Costa, Davi D. Bock, Paul A. Garrity, Gregory S.X.E. Jefferis

**Affiliations:** 1 Department of Zoology, University of Cambridge, Cambridge CB2 3EJ, UK; 2 Department of Biology, Brandeis University, Waltham, MA 02454, USA; 3 Division of Neurobiology, MRC Laboratory of Molecular Biology, Cambridge, Cambridgeshire CB2 0QH, UK; 4 Janelia Research Campus, Howard Hughes Medical Institute, Ashburn, VA 20147, USA; 5 Larner College of Medicine, University of Vermont, Burlington, VT 05405, USA

**Keywords:** thermosensation, hygrosensation, connectomics, *Drosophila*, antennal lobe, projection neuron, mushroom body, lateral horn, lateral accessory calyx, circadian clock

## Abstract

Animals exhibit innate and learned preferences for temperature and humidity—conditions critical for their survival and reproduction. Leveraging a whole-brain electron microscopy volume*,* we studied the adult *Drosophila melanogaster* circuitry associated with antennal thermo- and hygrosensory neurons. We have identified two new target glomeruli in the antennal lobe, in addition to the five known ones, and the ventroposterior projection neurons (VP PNs) that relay thermo- and hygrosensory information to higher brain centers, including the mushroom body and lateral horn, seats of learned and innate behavior. We present the first connectome of a thermo- and hygrosensory neuropil, the lateral accessory calyx (lACA), by reconstructing neurons downstream of heating- and cooling-responsive VP PNs. A few mushroom body-intrinsic neurons solely receive thermosensory input from the lACA, while most receive additional olfactory and thermo- and/or hygrosensory PN inputs. Furthermore, several classes of lACA-associated neurons form a local network with outputs to other brain neuropils, suggesting that the lACA serves as a hub for thermo- and hygrosensory circuitry. For example, DN1a neurons link thermosensory PNs in the lACA to the circadian clock via the accessory medulla. Finally, we survey strongly connected downstream partners of VP PNs across the protocerebrum; these include a descending neuron targeted by dry-responsive VP PNs, meaning that just two synapses might separate hygrosensory inputs from motor circuits. These data provide a comprehensive first- and second-order layer analysis of *Drosophila* thermo- and hygrosensory systems and an initial survey of third-order neurons that could directly modulate behavior.

## Highlights

• Two novel thermo- and/or hygrosensory glomeruli in the fly antennal lobe
• First complete set of thermosensory and hygrosensory projection neurons
• First connectome for a thermo- and hygrosensory neuropil
• Third-order thermo- and hygrosensory neurons, including link to circadian clock

## Graphical Abstract

**Figure:**
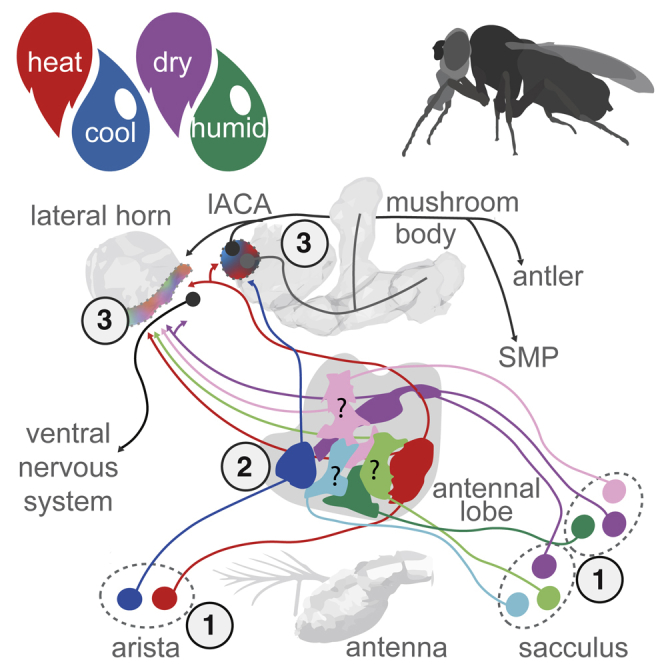

## Teaser

Marin et al. use connectomics and genetics for comprehensive identification of temperature and humidity sensory neurons in the *Drosophila* brain. They reconstruct all projections to higher brain areas and select higher-order targets, including the mushroom body lateral accessory calyx, linking thermosensation to memory and the circadian clock.

## Introduction

Temperature and humidity are interrelated environmental variables with dramatic effects on animal physiology and survival. Temperature affects all aspects of organismal function, making the maintenance of optimal body temperature and avoidance of thermal extremes critical for survival [1]. Humidity impacts hydration state, especially in insects, whose large surface-area-to-volume ratios make them vulnerable to dehydration [2]. Temperature and humidity also cue essential behavioral programs; for example, female mosquitoes use heat and humidity to locate warm-blooded hosts for blood feeding [3, 4], and temperature can entrain the circadian clock [5, 6], regulating rhythms of activity and sleep.

While fundamental for survival, the neuronal mechanisms of thermosensation and hygrosensation are not well understood. In mammals, the molecules and circuits underlying thermal nociception have been extensively investigated, but less is known regarding body temperature homeostasis [7]. In insects, hygro- and thermosensory neurons have been found on the antennae of diverse species, with individual sensilla often containing pairs of cooling- and heating-responsive neurons or triads of cold-, dry-, and humid-responsive neurons [8]. These neurons (like olfactory sensory neurons) project from the antennae to the antennal lobe (AL) in the brain, where they innervate stereotyped glomerular subcompartments [9]. Projection neurons (PNs) relay information from these glomeruli to higher brain regions including the mushroom body calyx (CA) and lateral horn (LH) of the protocerebrum [10], respectively involved in learned and innate olfactory behaviors [11].

Recent studies in *Drosophila melanogaster* provide some insight into the molecules and brain circuits underlying insect thermosensation and hygrosensation [12]. In flies, the third antennal segment contains two structures housing thermo- and hygrosensory neurons: a feathery protrusion called the arista and a multi-chambered invagination called the sacculus [13, 14]. The arista houses three (sometimes four) pairs of phasic thermosensory neurons, one transiently activated by heating and the other by cooling [15, 16]. The sacculus contains several types of sensilla, some of which resemble thermo- and/or hygrosensory sensilla [17]. Dry-responsive neurons have been identified in chambers I and II [18, 19] and humid-responsive neurons in chamber II [20, 21].

**Figure 1:**
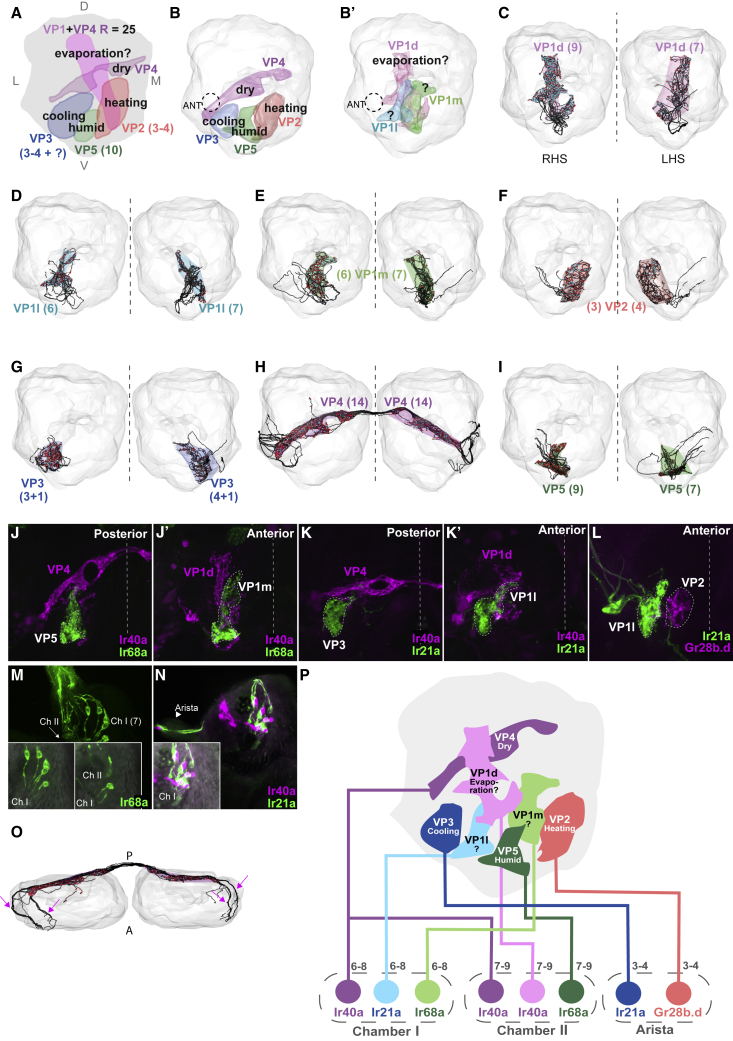
Sensory Neurons Define Seven Distinct Glomeruli in the Ventroposterior Antennal Lobe (A) Frontal view illustration of the VP AL summarizing the sensory modalities, locations, and numbers of thermo- and hygrosensory neurons reported in previous studies. D: dorsal; L: lateral; M: medial; V: ventral. (B and B’) Glomerular meshes generated from sensory neurons reconstructed in the right hemisphere of FAFB. Dashed circle labeled ANT: right antennal nerve. (B) Posterior meshes: VP2, VP3, VP4, and VP5. (B’) Anterior meshes: VP1d, VP1l, and VP1m. (C–I) ALs with glomerular meshes enclosing reconstructions of individual VP sensory neurons (in black, with presynaptic sites in red and postsynaptic sites in cyan, and number of neurons indicated in parentheses). Dashed line: the midline. (C) VP1d, (D) VP1l, (E) VP1m, (F) VP2, (G) VP3, (H) VP4, (I) VP5. (J–L) Frontal views of receptor expression data in the AL. White dashed line: the midline. (J and J’) VP1m and VP5 sensory neurons express Ir68a (green) but not Ir40a (magenta). (K and K’) VP1l and VP3 sensory neurons express Ir21a (green) but not Ir40a (magenta). (L) VP2 sensory neurons express Gr28b.d (magenta), but Ir21a+ VP1l sensory neurons (green) do not. (M) Chamber I and II sensory neurons express Ir68a (green). Inserts show bright field overlay with GFP of selected frames. (N) Chamber I sensory neurons express Ir21a (green). Inserts show bright field overlay with GFP of selected frames. Arrowhead: Ir21a+ neurons in the arista. (O) Dorsal view of VP4 RNs. Arrows: distinct axon bundles from each antennal nerve. (P) A model of the organization of thermo- and hygrosensory neurons in the sacculus and arista, with the specific receptors they express and the VP AL glomeruli they innervate. See also Figures S1 and S2.

Antennal thermo- and hygrosensory neurons innervate ventroposterior (VP) AL glomeruli. Heating-responsive aristal thermosensory neuron axons expressing the gustatory receptor isoform Gr28b.d [22] arborize in VP2 and cooling-responsive ones expressing the ionotropic receptor Ir21a [16] in VP3 [15, 23]. Saccular Ir40a-expressing, dry-air-responsive neurons arborize in VP4 [18, 19, 20, 24] and Ir68a-expressing, humid-air-responsive ones in VP5 [20, 21]. Finally, Ir40a-expressing putative thermosensors in chamber II project to VP1 [18, 19, 24] (Figure 1A); these have been reported to respond weakly to cooling [18], dry air [19], or ammonia [25] and may represent evaporative cooling.

Several PN classes relay information from VP glomeruli to higher brain centers. For example, a slow-adapting, cool-sensing (“slow-cool”) VP3 PN targets the lateral accessory calyx (lACA) of the mushroom body [26, 27]. Fast-adapting thermosensory PNs target the posterior lateral protocerebrum (PLP) and posterior slope [27, 28], and a broadly tuned PN innervating several VP glomeruli targets the PLP [21]. These studies suggest some integration of thermo- and hygrosensory inputs at the level of the AL PNs and indicate that these pathways project to multiple brain regions.

Despite these advances, our current description of thermo- and hygrosensory systems in *Drosophila* remains incomplete. The sacculus contains uncharacterized sensory neurons that likely innervate unknown AL glomeruli. Uniglomerular PNs have not yet been reported for some known VP glomeruli. Finally, the only higher-order neurons identified thus far are α’/β’ Kenyon cells (KCs) of the mushroom body, presumed targets of the slow-cool VP3 PN in the lACA [29].

To obtain a more comprehensive view of the thermo- and hygrosensory system, we used the full adult fly brain (FAFB) electron microscopy (EM) volume for whole neuron reconstruction and synapse annotation [30]. We identified the sensory neurons innervating five known VP glomeruli and two novel glomeruli and showed that these neurons express receptor molecules consistent with thermosensation or hygrosensation. We also reconstructed every PN projecting from VP glomeruli to higher brain centers; they are highly diverse in tract, neuropil target, and neuroblast of origin. We elucidated the “connectome” of the lACA, which not only provides input to both dedicated and integrative KCs but also serves as a hub for thermo- and hygrosensory information and a link to the circadian clock. Finally, we identified additional novel third-order neurons, including a descending neuron that relays hygrosensory information to the ventral nerve cord. Together, these data provide a comprehensive catalog of first- and second-order *Drosophila* thermo- and hygrosensory neurons and an initial survey of third-order neurons that would allow integration with other sensory modalities and modulation of behavior.

## Results

### Sensory Neurons Define Two Novel Glomeruli in the Ventroposterior Antennal Lobe

To define the five known VP glomeruli (Figure 1A), we reconstructed all putative VP sensory neurons in both hemispheres of FAFB, completing (including synapses) those in the right-hand side (RHS) AL. We found four known VP glomeruli in the posteriormost AL (Figure 1B): VP2 (heating), VP3 (cooling), VP4 (dry), and VP5 (humid).

Three unilateral receptor neurons (RNs) with complex arbors on the RHS and four on the left-hand side (LHS) innervated VP2 (Figure 1F). Four RHS unilateral RNs (five LHS) innervated VP3 (Figure 1G), with all but one forming complex arbors (Figures S1E’ and S1E’’) and the last forming a simpler arbor and traveling separately (Figure S1E’’’). Morphological clustering with NBLAST [31] paired the simpler VP3 RNs on each side with one another, within the larger VP3 RN group (Figure S2A), implying that they are of the same type and distinct from the complex VP3 RNs. Moreover, the simpler VP3 RN provided most of the input to the RHS slow-cool VP3 PN, whereas the others targeted distinct VP3 PNs (Figure S2B). These results are consistent with reports of three to four pairs of aristal sensory neurons that project to VP2 (heating) and VP3 (cooling) [15, 16] and an unknown number of non-aristal sensory neurons supplying the slow-cool VP3 PN [27].

We traced 28 bilateral RNs that collectively defined the VP4 glomeruli (Figure 1H). These VP4 RNs traveled in two distinct areas of each antennal nerve (Figure 1O), perhaps reflecting their origins in sacculus chambers I versus II. We also found nine unilateral RHS (seven LHS) RNs occupying VP5 (Figure 1I), consistent with previous reports [20]. Since VP4 and VP5 RNs are found in the same triad sensilla in chamber II [20, 21, 32], this suggests that nine of 14 RHS VP4 RNs (seven of 14 LHS RNs) originated in chamber II and the remainder in chamber I.

To our surprise, unilateral RN axons in the predicted location of VP1 formed not one, but three distinct populations. These defined adjacent glomeruli (Figure 1B’) that we designated according to their relative positions: VP1d, VP1l, and VP1m (Figures 1C–1E). NBLAST clustering supported six distinct classes of unilateral RNs: VP1d, VP1l, VP1m, VP2, VP3, and VP5 (Figure S2A). VP1d, VP1l, and VP1m RNs each formed synaptic connections with other neurons from the same glomerulus, consistent with our separate designations (Figure S2C).

VP1d RNs most closely resembled the Ir40a-positive “VP1” neurons in morphology and location. Light-level studies (relying on IR40a-specific antibodies and drivers) had identified a total of 25 saccular sensory neurons projecting to VP1 and/or VP4 [19, 20, 24], consistent with our VP1d and VP4 RN totals (23 RHS and 21 LHS). Moreover, we found the same numbers of VP1d and VP5 RNs in each AL (Figures 1C and 1I), suggesting that these classes might cohabit the triad sensilla in chamber II, along with the VP4 RNs [20, 21, 32].

To elucidate the likely modalities of VP1l and VP1m, we generated novel tools to label neurons expressing receptors previously associated with thermo- and hygrosensation (STAR Methods). We observed Ir68a expression in VP1m RNs as well as in VP5 (Figures 1J and 1J’). Ir21a (Figures 1K and 1K’), but not Gr28b.d (Figure 1L), was expressed in VP1l RNs, suggesting that they may be cooling responsive. These expression data, together with their characteristic neuronal and sensillar morphologies, suggest these new VP sensory neurons are thermo- or hygrosensory. Finally, Ir68a (Figure 1M) and Ir21a expression (Figure 1N) were each detected in approximately seven neurons of chamber I, and the same numbers of VP1l and VP1m RNs were reconstructed in each AL (Figures 1D and 1E). These data suggest that VP1l and VP1m RNs form a triad with the Ir40a-expressing VP4 neurons in chamber I (Figure 1N).

Our data, along with published expression data, support a model in which VP2 and VP3 are innervated by heating- and cooling-responsive sensory neurons from the arista and five additional VP glomeruli by sensory neurons from the sacculus (Figure 1P). Sensory neurons from seven to nine triad sensilla in chamber II innervate VP4 (Ir40a, dry), VP5 (Ir68a, humid), and VP1d (Ir40a, putative evaporative cooling). Sensory neurons from six to eight sensilla in chamber I innervate VP4 (Ir40a, dry), VP1l (Ir21a, putative cold/cooling), and VP1m (Ir68a, putative humid).

### A Diverse Population of Antenna Lobe Projection Neurons Relays Thermo- and Hygrosensory Information to Higher Brain Centers

**Figure 2:**
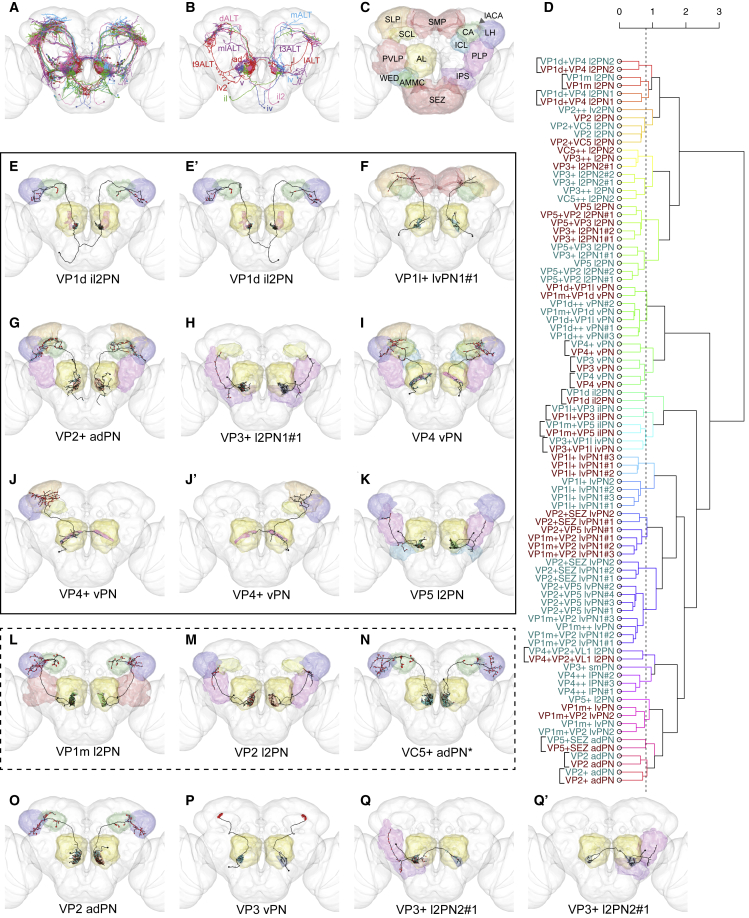
Projection Neurons Relay Information from Individual VP Glomeruli to Higher Brain Centers (A) Frontal view of all VP PNs, color-coded by primary glomerulus. (B) Summary diagram of the tracts and neuroblast lineages of VP PNs. (C) Summary diagram of the target brain neuropils of VP PNs, color-coded to correspond with all other figure panels. AL: antennal lobe; AMMC: antennal mechanosensory and motor center; CA: mushroom body calyx; ICL: inferior clamp; IPS: inferior posterior slope; lACA: lateral accessory calyx; LH: lateral horn; PLP: posterior lateral protocerebrum; PVLP: posterior ventral protocerebrum; SCL: superior clamp; SEZ: subesophageal zone; SLP: superior lateral protocerebrum; SMP: superior medial protocerebrum; WED: wedge. (D) Morphological hierarchical clustering based on NBLAST scores for VP PNs in each hemisphere, cut at height 0.8 (dashed line), which groups the PNs by type. Dark cyan: RHS. Dark red: LHS. Square brackets: unique VP PNs matched across hemispheres. For each type with multiple examples, numbers (#1, etc.) reflect order of appearance in the SKID_R or SKID_L columns in Table 1. (E–Q’) Frontal views of reconstructions (black) of candidate thermo- and hygrosensory PNs that innervate one primary glomerulus (color-coded as in Figures 1B and 1B’, non-VP glomeruli in gray). Presynaptic sites in red and postsynaptic sites in cyan. Neuropils color-coded as in (C). Asterisks: primarily olfactory PNs; black outline: candidate novel thermosensory PNs; dashed outline: PNs previously described but not recognized as VP or thermosensory. (E) VP1d il2PN, (E') VP1d il2PN, (F) VP1l+ lvPN1#1, (G) VP2+ adPN, (H) VP3+ l2PN1#1, (I) VP4 vPN, (J) VP4+ vPN, (J') VP4+ vPN, (K) VP5 l2PN, (L) VP1m l2PN, (M) VP2 l2PN, (N) VC5+ adPN*, (O) VP2 adPN, (P) VP3 vPN, (Q) VP3+ l2PN2#1, (Q') VP3+ l2PN2#1.

We next sought to identify the projection neurons (PNs) that relay activity from VP AL glomeruli to higher brain centers. By systematic tracing of the AL tracts, we identified every PN on the RHS, including 62 innervating a VP glomerulus more than any other ([33], this issue of *Current Biology*) (Figure 2A; Table 1). Ten of these are still predicted to receive majority olfactory input; the remaining 52 were designated “VP PNs” and classified into 38 morphological types, using lineage, tract, glomerulus, and axon arborization (Table 1, Type).

**Table 1:** Candidate Thermo- and Hygrosensory Projection Neurons

Type	Tract	Panel	SKID_R	SKID_L	FC Match	Score	Novel	Previous Names	Driver	Score	NT
VP2 adPN	mALT	2O	57516	9159128	Gad1-F-000090	0.615	false	VP2 uACT1 [23]	R21C11	0.568	ACh
VP2+ adPN	mALT	2G	1712057	8864990		NA	true			NA	ACh
VP5+SEZ adPN	mALT	3L	14003783	9511430		NA	true^∗^	poly[emb] adPN [34]		NA	ACh
VP1l+VP3 ilPN	mALT	3B, 3B’	57495	57487	Cha-F-100277	0.656	false	VP3 bilateral PN [23]	R82F02	0.521	
VP1m+VP5 ilPN	mALT	3A, 3A’	57503	46105	Gad1-F-300444	NA	false	R24G07 hPN [21]	R24G07-L [21]; R25B12	NA; 0.470	
VP1d il2PN	dALT	2E, 2E’	192423	203504	VGlut-F-000344	0.571	true		R18A08	0.531	
VP3+VP1l ivPN	mALT	3C, 3C’	45882	37513		NA	false	slow cold mALT PN [28]	VT26020 [28]; R80F03	NA; 0.636	
VP4++ lPN	mALT	S3H, S4E, S4E’	3648956, 57430, 57434		Gad1-F-700019	0.657	true		R82C09	0.679	ACh
VP4+VP2+VL1 l2PN	t3ALT	3M, 3M’	1664544	4631122		NA	false	warm-cool PN [27]	R54A03 [27]; R82C09	NA; 0.603	
VP5+ l2PN	mALT	S3E	10078400			NA	true				
VP1d+VP4 l2PN1	lALT	3D, 3D’	23005	9716626		NA	true^∗^	biPN-2 [35]	R38A04	0.517	
VP1d+VP4 l2PN2	lALT	3E	3908507	9740174	Cha-F-500003	0.683	true^∗^	VP3 oACT [23]	R59B04	0.641	
VP1m l2PN	lALT	2L	11524119	3078	Cha-F-500056	0.655	true^∗^	unPN-2 [35]; lPN1 [36]		NA	
VP2 l2PN	lALT	2M	5672990	13154015		NA	true^∗^	unPN-4 [35]	VT046265	NA	
VP2+VC5 l2PN	lALT	3N, 3N’	2600341	10515783	Cha-F-100163	0.684	true^∗^	biPN-3 [35]	R32H03?	0.609	
VP3+ l2PN1	t10ALT	2H, S4C	11234968	13159728,13159715	Trh-M-300005?	0.605	true		R95C02	0.638	
VP3+ l2PN2	t10ALT	2Q, 2Q’, S4A	4869882, 1356477	13159741	Trh-M-300005	0.699	false	fast-cool PN [27]	R95C02 [27]	0.627	
VP3++ l2PN	t10ALT	S3D, S3D’	7017522	5946637		NA	true		R95C02?	0.506	
VP5 l2PN	t10ALT	2K	4672650	13053419	Cha-F-100123	0.680	true		R78E05	0.588	
VP5+VP2 l2PN	t10ALT	S3A, S4B	5946848, 4876532	13159719	Cha-F-100123	0.693	false	warm-PN [27]	R95C02 [27]; R78E05	NA; 0.571	
VP5+VP3 l2PN	t10ALT	S3B	4671556	9704839	Trh-M-200074	0.710	false	unPN-6 [35]; cool lALT PN [28]	R95C02 [28]	0.593	
VP1l+ lvPN1	mALT	2F, S4F, S4F’	57224, 56999, 57200	4177248, 7463926, 13492671		NA	true		R14F11?	0.564	ACh
VP1l+ lvPN2	mALT	S3I	3742499			NA	true		R14F11?	0.491	ACh
VP1m++ lvPN	mALT	S3J	57142			NA	true			NA	ACh
VP1m+VP2 lvPN1	mALT	3F, 3F’, 3F’’	57122, 57059, 57114	9101937, 12681995, 13931917		NA	true		R31H04	0.629	ACh
VP2+VP5 lvPN	mALT	S3M, S4G, S4G’, S4G’’	57138, 57134, 57051, 1372988	11052217		NA	true		R31H04	0.601	ACh
VP2+SEZ lvPN2	mALT	S3K	57063	4495405	VGlut-F-700270?	0.579	true		R31H04?	0.539	ACh
VP2+SEZ lvPN1	mALT	3K, S4I, S4I’	57166, 57146	7423431	VGlut-F-700270	0.606	true		R31H04?	0.558	ACh
VP1m+ lvPN	t2ALT	3J	192430	9588978		NA	Ttrue		R24B03	0.657	
VP1m+VP2 lvPN2	t2ALT	3I	4058666	9590169		NA	true		R24B03	0.615	
VP2+ lv2PN	t9ALT	S3N	4002166			NA	true		R93D06?	0.564	
VP3+ smPN	mALT	S3O	57475		VGlut-F-200354?	0.628	true		R82F02	0.549	
VP1d++ vPN	mlALT	S3P, S4I, S4I’	3813447, 3813442, 4632023		VGlut-F-500199	0.682	true		R49F09	0.694	GABA
VP1d+VP1l vPN	mlALT	S3Q	3813438	5643689		NA	true		R21C11	0.660	GABA
VP1m+VP1d vPN	mlALT	S3S	3813424	13272972		NA	true		R21C11	0.675	GABA
VP4+ vPN	mlALT	2J, 2J’	2484510	12805737		NA	true		R11F08	0.600	GABA
VP3 vPN	mlALT	2P	5471515	561100	Fru-M-100050	0.523	false	AL-t5PN1 [26]; slow-cool PN [27]	R60H12 [28]	0.600	GABA
VP4 vPN	mlALT	2I	1149173	9039579	VGlut-F-300564	0.659	true		R21A01	0.616	GABA
VP1d++ lPN1^∗^	mALT	S3F, S4D-S4D’’’	37235, 37212, 182684, 57426, 57446		VGlut-F-200115	0.663	true		R47G06?	0.666	ACh
VP1l++ lPN^∗^	mALT	S3G	57442		Cha-F-300107	0.636	true		R41H09	0.616	ACh
VP1m++ l2PN^∗^	lALT	S3C	4195469	14005954	VGlut-F-000370?	0.541	true^∗^	unPN-5 [35]	R47A12	0.605	
VP2++ lvPN^∗^	mALT	S3L	57126			NA	true		R14F11?	0.494	ACh
VP1m++ smPN^∗^	t6ALT	6D	11546775	9717951		NA	true		R82F02?	0.486	
VP1m++ vPN^∗^	mlALT	S3R	4954519			NA	true		R49F09?	0.680	GABA
VC5+ adPN^∗^	mALT	2N	39254	3420237	Cha-F-200265	0.579	true^∗^	VM6+VP1 adPN [34]		NA	ACh
VC5++ l2PN1^∗^	t3ALT	3O, 3O’	1363077, 8825246	5122761, 13950267	VGlut-F-100102	0.693	false	slow hot and cold t3ALT PN [28]	R84E08 [28]; R82C09; R13B06	NA; 0.584; 0.538	
VC5++ l2PN2	t10ALT	S3T, S3T’	8406430	5582907		NA	true^∗^	biPN-4 [35]		NA	
VC5+ lvPN1^∗^	mALT	S3U, S3U’, S4J	57130, 57236	4724830		NA	true		R53D01?	0.538	ACh
VC5+ lvPN2^∗^	mALT	S3V, S4K	57015, 57175			NA	true		R14F11?	0.516	ACh

Type: our designation, based on neuroblast lineage and top glomerulus innervated. Asterisk indicates PN predicted to be primarily olfactory. Tract: antennal lobe tract. Panel: corresponding panel(s) in this paper. SKID_R: RHS skeleton IDs (first listed is #1, etc.). SKID_L: LHS skeleton IDs (first listed is #1, etc.). FC Match: best corresponding single-cell clone in FlyCircuit. Score: Average of forward and reverse NBLAST scores for FC match. Previous Names: name(s) previously assigned to this type in the literature. Driver: sparse driver line(s) that label this neuron, based on previous reports and/or our NBLAST. Score: forward NBLAST score for driver. NT: likely neurotransmitter, based on neuroblast lineage and tract (please see [33] for details). See also Figures S3 and S4.

We also reconstructed 36 VP PNs on the LHS and matched them to RHS types. NBLAST clustering helped confirm morphological stereotypy across brain hemispheres; 14 uniquely identifiable VP PNs paired with their contralateral counterparts instead of with ipsilateral VP PNs of similar type (Figure 2D). Of our 38 types, nine had been described in the thermo- and hygrosensory light-level literature, while six had been reported but not associated with the correct glomeruli (e.g., [34, 35]) (Table 1, Previous Names), leaving 23 completely novel VP PN types.

Our reconstructed VP PNs constituted a highly diverse population. Based on their primary soma tracts, they belonged to nine distinct neuroblast lineages or hemilineages: AL anterodorsal (ad), lateral (l), ventral (v), or lateroventral (lv) [37], or the previously unidentified lateroventral 2 (lv2), inferior lateral (il), inferior lateral 2 (il2), inferior ventral (iv), or superior medial (sm) [33]. They projected to higher brain centers via any of seven AL tracts: the mALT, mlALT, and lALT; one of the small transversal tracts leaving those main tracts [36]; or an unusual route we dubbed the dALT (Figure 2B; Table 1). Like canonical excitatory olfactory PNs, many VP PNs targeted the CA and LH, but some targeted additional neuropils (Figure 2C).

**Figure 3:**
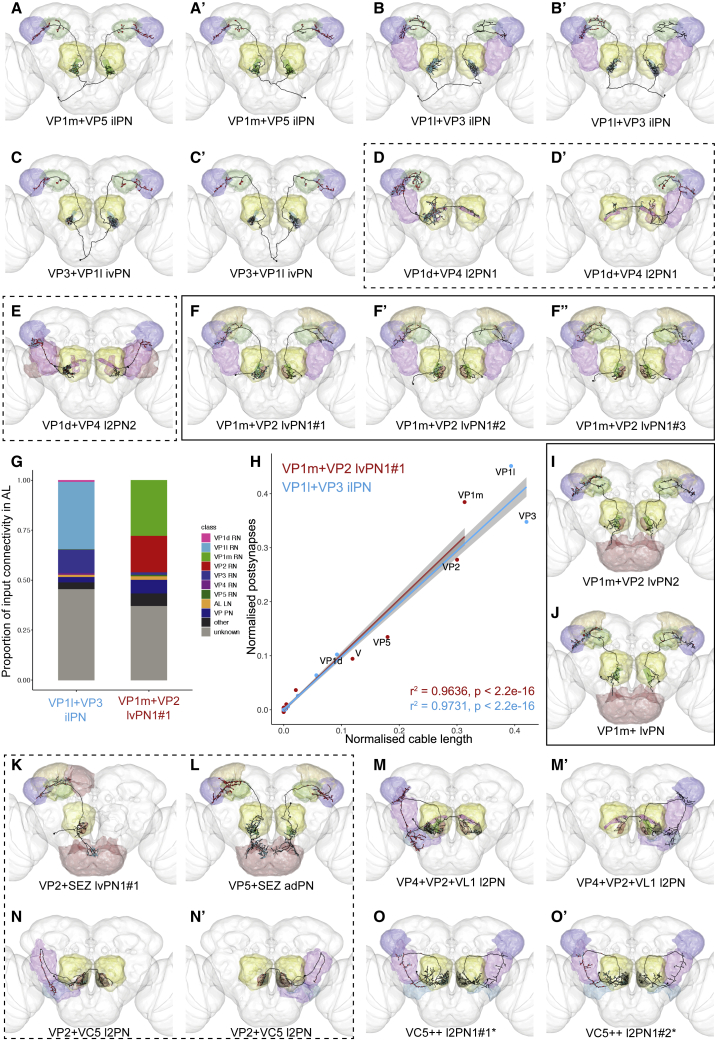
Projection Neurons Relay Information from Multiple VP Glomeruli and Other Modalities to Higher Brain Centers (A–F’’’) Frontal views of reconstructions (black) of candidate thermo- and hygrosensory PNs that innervate more than one VP glomerulus. (A) VP1m+VP5 ilPN, (A') VP1m+VP5 ilPN, (B) VP1l+VP3 ilPN, (B') VP1l+VP3 ilPN, (C) VP3+VP1l ivPN, (C') VP3+VP1l ivPN, (D) VP1d+VP4 l2PN1, (D') VP1d+VP4 l2PN1, (E) VP1d+VP4 l2PN2, (F) VP1m+VP2 lvPN1#1, (F') VP1m+VP2 lvPN1#2, (F'') VP1m+VP2 lvPN1#3. (G and H) Proportion of inputs of each type (G) and best fit line to linear regression model plotted in R with the lm() function showing correlation of cable length in each glomerulus with RN input for two RHS multiglomerular VP PNs, VP1m+VP2 lvPN1#1 and VP1l+VP3 ilPN (H). (I–L) Frontal views of reconstructions (black) of candidate thermo- and hygrosensory PNs that innervate the subesophageal ganglion (red) as well as VP glomeruli. (I) VP1m+VP2 lvPN2, (J) VP1m+ lvPN, (K) VP2+SEZ lvPN1#1, (L) VP5+SEZ adPN. (M–O’) Frontal views of reconstructions (black) of candidate thermo- and hygrosensory PNs that innervate non-VP glomeruli. Presynaptic sites in red and postsynaptic sites in cyan. Brain neuropils color-coded as in Figure 2C. VP glomeruli color-coded as in Figures 1B and 1B’; non-VP glomeruli in gray. Black outline: candidate novel thermosensory PNs; dashed outline: PNs previously described but not recognized as VP or thermosensory, or associated with the wrong glomeruli. (M) VP4+VP2+VL1 l2PN, (M') VP4+VP2+VL1 l2PN, (N) VP2+VC5 l2PN, (N') VP2+VC5 l2PN, (O) VC5++ l2PN1#1•, (O') VC5++ l2PN1#2*.

Each VP glomerulus except VP1l was densely and specifically innervated by at least one “uniglomerular” PN (Figures 2E–2Q’), validating the boundaries of the VP glomeruli and our distinction between VP1d, VP1l, and VP1m. However, most VP PN types innervated multiple glomeruli (Figure 3); the most common “biglomerular” combinations were VP1l and VP3, VP1m and VP2, VP1d and VP4, and VP5 and VP2 (Table 1), suggesting that these represent stimulus combinations of particular behavioral importance. We selected two examples (VP1l+VP3 and VP1m+VP2) and completely reconstructed their dendrites in the RHS AL, finding that they sampled substantially, but not equally, from each glomerulus (Figures 3G and 3H).

Numerous PNs innervated olfactory or gustatory neuropils in addition to VP glomeruli (Figures 3K–3O’). We identified VP PNs innervating either VP2 or VP5 [34] and the subesophageal zone (SEZ) (Figures 3K and 3L), presumably integrating temperature or humidity with taste. At least eight PNs innervated VP1d together with olfactory glomeruli (Table 1), perhaps adjusting signal strength based on rate of evaporation. Finally, we found one VC5+VP2 PN (Figures 3N and 3N’) and one VC5 PN predicted to receive primarily VP input (Figures S3T and S3T’). Multiglomerular PNs innervating VC5 (Figures 3O and 3O’) have previously been implicated in thermosensation [28].

In summary, we reconstructed 88 VP PNs of 38 putative morphological types, plus 5 types of PNs innervating VC5. We found anatomical (and for seven, functional) descriptions of 15 VP PN types in the *Drosophila* neurobiology literature (Table 1, Previous Names). Elucidation of our 23 novel VP PN types awaits future physiological and behavioral studies, for example, using sparse driver lines (Table 1, Driver).

### VP PNs Relay Thermo- and Hygrosensory Information to the Lateral Accessory Calyx

**Figure 4:**
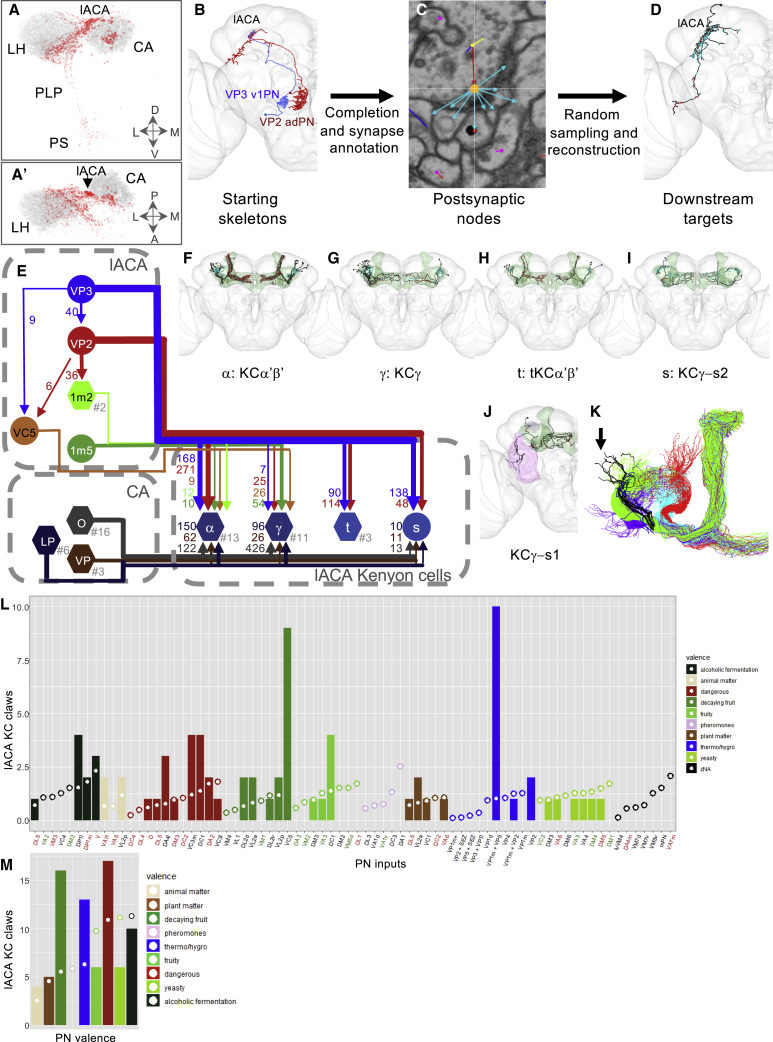
Both Dedicated and Mixed Modality Kenyon Cells Receive Thermosensory Input from the Lateral Accessory Calyx (A and A’) Frontal (A) and dorsal (A’) views of VP PN presynapses (red) versus olfactory PN presynapses (gray). CA: mushroom body main calyx; LH: lateral horn; PLP: posterior lateral protocerebrum; PS: posterior slope. (B–D) Workflow for randomized downstream sampling from key VP PNs in the lACA. (B) Frontal view of reconstructed VP PNs used for sampling from the lACA in the right hemisphere of FAFB. (C) Annotation of presynapses and associated postsynapses from these two VP PNs in the lACA. Orange: presynapse; cyan arrow: edge to associated postsynapse. (D) Frontal view of an example target neuron reconstructed during the sampling process. (E) Partial circuit diagram illustrating connectivity between lACA-associated KCs receiving at least five inputs in the lACA and their VP and olfactory inputs. Circular nodes represent individual neurons; hexagonal nodes represent pooled neurons, with the number pooled after the hashtag. Synapse numbers correlate with line thicknesses. lACA: PNs targeting KCs in the lACA; VP3: VP3 vPN; VP2: VP2 adPN; 1m2: VP1m+VP2 lvPN1#1-2; VC5: VC5+ adPN; 1m5: VP1m+VP5 ilPN; CA: olfactory PNs (O), lACA PNs (LP), and other VP PNs (VP) targeting KC dendrites in the main calyx. (F–J) Frontal views of lACA-associated KCs. Green: whole mushroom body; purple: dACA (J only). (F) α: KCα'β', (G) γ: KCγ, (H) t: tKCα'β', (I) s: KCγ-s2, (J) KCγ-s1. (K) lACA-associated KCs (black) derive from one (green) of four mushroom body neuroblast lineages (green/cyan/red/violet). (L) Expected (circles) versus observed (bars) numbers of lACA KC dendritic claws receiving input from olfactory and VP PN partners in the CA (color-coded by valence, key on right). PN inputs labeled as aversive (red), attractive (green), or unknown (black). (M) Summary of expected (circles) versus observed (bars) numbers of lACA KC dendritic claws receiving input of various valences (key on right) from olfactory and VP PN partners in the CA. See also Figure S5 and Table S1.

Although the VP PNs target many different neuropils, the majority project to the CA and/or LH. Moreover, most presynaptic terminals are restricted to the lACA or anterior part of the main calyx, or the VP LH bordering the PLP (Figures 4A, 4A’, and S5A). We decided to focus our tracing efforts on the lACA, which had the highest density of VP PN innervation (Figures S5B and S5C).

**Figure 5:**
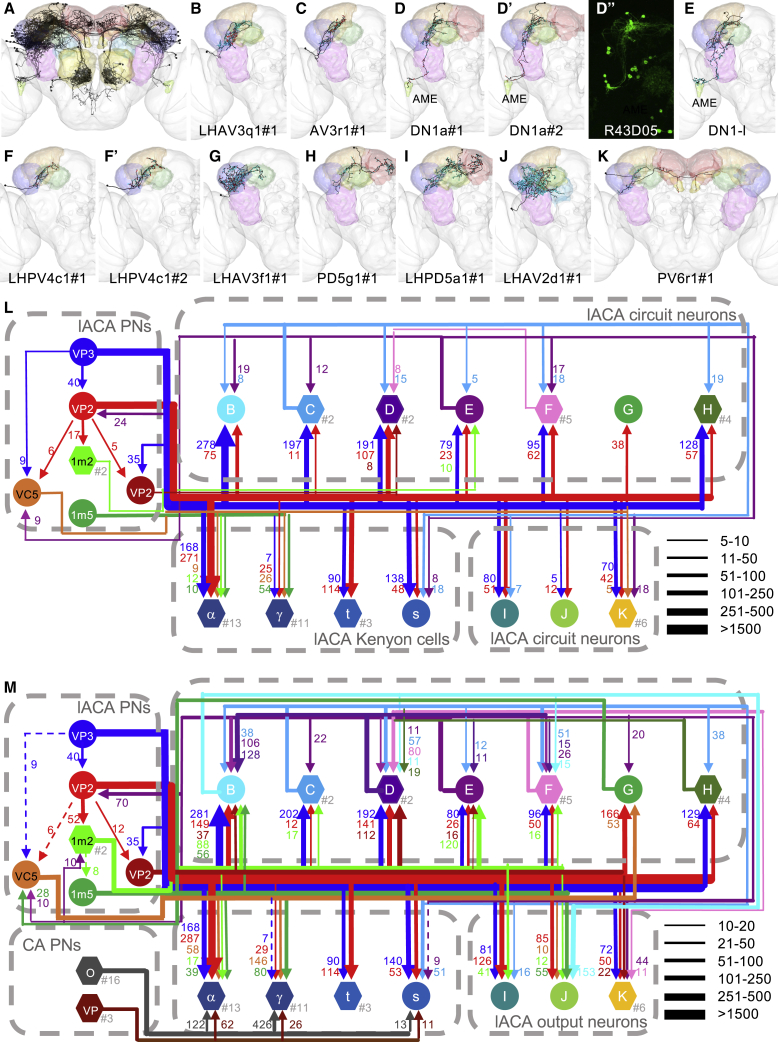
The Lateral Accessory Calyx Is a Thermo- and Hygrosensory Hub with Outputs to Multiple Neuropils (A–K, except D’’) Frontal views of reconstructions (black) of non-KC lACA target neurons. Brain neuropils have been color-coded to correspond with all other figure panels. (A) All identified targets with at least five connections to lACA PNs within the lACA. (B–K) Strong downstream targets of lACA PNs. Presynaptic sites in red and postsynaptic sites in cyan. (B) LHAV3q1#1, (C) AV3r1#1, (D) DN1a#1, (D') DN1a#2, (E) DN1-l, (F) LHPV4c1#1, (F') LHPV4c1#2, (G) LHAV3f1#1, (H) PD5g1#1, (I) LHPD5a1#1, (J) LHAV2d1#1, (K) PV6r1#1. AME: accessory medulla. (D’’) Gal4 driver line labeling DN1a neurons (source: Fly Light). (L–M) Circuit diagrams depicting (L) within-lACA and (M) total connectivity between lACA PNs and their strong downstream targets. Letters correspond to those of panels in this figure (except for elements carried over from Figure 3 and dark red VP2: VP2+ adPN). Circular nodes represent individual neurons; hexagonal nodes represent pooled neurons, with the number pooled after the hashtag. Synapse numbers correlate with line thicknesses, with dashed arrows in (M) representing inputs from (L) of <10 connections. CA PNs: olfactory PNs (O) and VP PNs (VP) targeting KC dendrites in the main calyx. lACA output neurons: neurons receiving input from lACA PNs that have <10 outputs to other lACA circuit neurons. See also Figure S6 and Table S2.

Only the slow-cool VP3 vPN was previously reported to innervate the lACA [26, 27]. Complete reconstruction of all RHS PN axons [33] revealed that 11 provide input to the lACA (Table S5), seven of which were conserved on the LHS: the VP3 vPN, the VP2 and VP2+ adPNs, a VP1m+VP5 ilPN, two VP1m+VP2 adPNs, and the VC5+ adPN. Axo-axonic connections between these PNs (Figures 4E, 5L, and 5M) may permit modulation of heating-responsive VP2 adPNs by the cooling-responsive VP3 vPN.

The VP3 vPN and VP2 adPN together provided the large majority (>90%) of PN presynapses to each lACA (Table S5). We annotated all of their associated postsynapses on the RHS and reconstructed a random sample (Figures 4B–4D), allowing identification of all strong downstream partners (STAR Methods; Figures S5C–S5C’’). We reconstructed the same strongly connected types for the LHS VP3 vPN, suggesting stereotypy (Figures 4F–4I, 5A–5K, and S6).

Distinct classes of KCs, KCγd, KCγ, KCα’/β’, KCα/βp, and KCα/β, are sequentially generated during development [38]. As expected from light-level studies [29], we recovered KCα’/β’ from sampling, three of which exclusively received input from the lACA (Figure 4H). We reconstructed 13 additional KCα’/β’ (Figure 4F) and 11 KCγ in the RHS lACA (Figure 4G) that also received olfactory and/or VP PN input in the CA (Figure 4E). We also reconstructed a KCγ (KCγ-s2) with unusually complex axon branching (Figure 4I) that received input from the lACA VP3 and VP2 PNs, multiglomerular PNs and thermo- and hygrosensory PNs in the CA (Figure 4E), and visual PNs in the dorsal accessory calyx (Table S1). All these lACA-associated KCs belonged to the lateral neuroblast lineage (Figure 4K). Finally, we identified a KCγ (Figure 4J) with complex axon branching and dendritic branches in the PLP and ventral accessory calyx, a visual neuropil [39, 40].

The main calycal inputs to lACA-associated KCs were not identical on both sides (Table S1). However, we found that lACA-associated KCs receive disproportionately more inputs in the main calyx from lACA PNs, especially the VC5+ adPN and VP1m+VP5 ilPNs (STAR Methods; Figure 4L). PNs activated by odors associated with decaying fruit or danger were also overrepresented; conversely, there were no inputs from pheromone-responsive PNs (Figure 4M). This suggests that, while the synaptic partners (and thus the sensory cues) of lACA-associated KCs are not completely predetermined, they may be biased during development.

Nearly two-thirds of PN inputs to the lACA were received, not by KCs, but by neurons projecting to other brain areas, primarily the LH, SMP (superior medial protocerebrum), and antlers (Figures 5A and S6; Tables S2 and S6). Many lACA target neurons received more input from lACA PNs outside of the lACA than inside (Figures 5L and 5M). Most were connected to other targets within the lACA (Figure 5L), while all were connected to at least one other target elsewhere (Figure 5M), forming a thermo- and/or hygrosensory network. In many cases, neurons of similar morphological type could not be distinguished by NBLAST (Figure S6) but exhibited distinct connectivity (e.g., Figures 5B–5E).

Notably, we reconstructed three neurons connecting the lACA and accessory medulla (Figures 5D, 5D’, and 5E) that resembled DN1 neurons [41], which can be entrained to temperature [42]. Two had very similar connectivity (Figure S6B), anterior somas, and morphologies (Figures 5D and 5D’) matching that of the DN1a subclass [43], and both were excellent NBLAST matches (Table S2) for DN1a driver R43D05 [44] (Figure 5D’’). We called the third DN1-l (for DN1-like) (Figure 5E); it had a more posterior soma, received strong input from visual PNs (Figure S6B), and was presynaptic to most lACA network neurons and to four out of seven lACA PNs (Figure 5M). This suggests a distinct role such as modulating circadian rhythms of temperature preference [45].

In summary, we found that seven PNs (representing VP3, VP2, VC5, VP1m+VP2, and VP1m+VP5) consistently targeted the lACA, providing inputs to dedicated thermosensory and integrative KCs (Figure 4), a partially overlapping subset of PNs, and at least 19 other neuron types (Figure 5; Table S2). Most lACA target neurons were not connected to Kenyon cells, but rather to each other, forming a local thermo- and/or hygrosensory network with outputs to circadian circuits and to brain regions implicated in innate and learned olfactory responses.

### VP PNs Project to Diverse Targets, Including at Least One Descending Neuron

We noticed that many targets receiving strong VP3 and VP2 input in the lACA also received VP PN input in other neuropils. We considered the entire population of VP PNs and identified the 18 most strongly connected reconstructed partners, with >150 inputs from VP PNs. However, since tracing in this dataset has been focused on the LH and CA, many VP PNs projecting to other neuropils shared no connections with those targets and were therefore excluded from further analysis.

**Figure 6:**
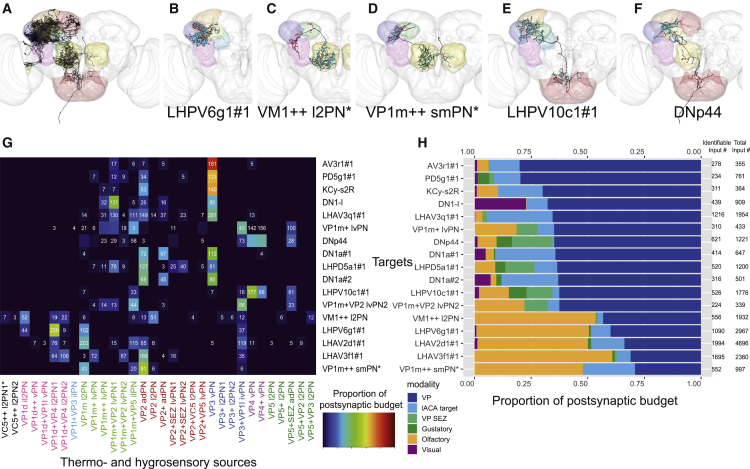
VP PNs Relay Information to Diverse Targets, Including a Descending Neuron (A–F) Frontal views of selected strong downstream targets of RHS and bilaterally symmetric VP PNs, with brain neuropils color coded as in Figure 2C. Reconstructed neurons in black, with presynaptic sites in red and postsynaptic sites in cyan. (A) Seventeen strong downstream targets of VP PNs. (B) Local LH neuron LHPV6g1#1. (C) VM1++ l2PN. (D) VP1m++ smPN^∗^. (E) LH and SEZ interneuron LHPV10c1#1. (F) Descending neuron DNp44. (G) Heatmap showing contributions by VP PN classes to 17 downstream targets, normalized with total VP PN input = 1. Cell numbers denote raw connectivity. Heatmap is thresholded >2 synapses. Source labels are color coded by top glomerulus. See also Table S3. (H) Summary stacked bar graph showing proportion of inputs from VP, VP + SEZ, gustatory, olfactory, and visual PNs, and lACA target neurons onto each of the 17 targets, normalized with total identified inputs = 1. Total inputs and total identified inputs are shown on the right. For both (G) and (H), only the dendrites of VM1++ l2PN and KCγ-s2R and the axon of VP1m++ smPN were considered. See also Table S3.

Of the strong targets (Figure 6A), two were themselves VP PNs with arbors in the VP LH (Figures 3J and 3K), and ten received significant input in the lACA (Figure 5). We selected five of the remaining six neurons to showcase their diversity: a local LH interneuron, two multiglomerular projection neurons, an LH-SLP (superior lateral protocerebrum) and SEZ connector, and a descending neuron (Figures 5B–5F). All received substantial VP PN input inside or at the border of the LH, except for the VM1++ l2PN, which received it via dendrodendritic synapses in the AL [33].

We tabulated all VP PN input (Table S3) for each of these neurons (Figure 6G), also noting the modality or category (Figure 6H). Strikingly, the VP3 vPN alone supplied ∼80% of the inputs to AV3r1#1, indicating that this target neuron is specialized for cooling. In contrast, LHAV3q1 was broadly tuned to thermo- and hygrosensory input from multiple VP glomeruli, demonstrating distinct innervation patterns of LH neurons of similar morphology. Along the same lines, the two putative DN1a neurons received very similar inputs, while DN1-l integrated inputs from the VP3 vPN and VP1m+VP2 lvPNs with substantial visual input.

The KCγ-s2 neuron received 70% of its inputs from VP PNs, primarily the VP3 vPN, but also ∼20% from lACA target neurons. This suggests that it has a special role in the mushroom body for more complex integration of thermosensory information in temperature memory or preference.

VP1d (possibly evaporation) and VP1m (possibly humidity) PNs were strong upstream partners of LH neurons with large proportions of their postsynaptic budgets dedicated to olfactory input. The LH is therefore a site of multimodal integration where innate valence responses might change with perceived concentration [46, 47], in turn dependent on environmental humidity conditions and/or volatility of the odorant.

Finally, the large proportion of dry-responsive VP4 PN input to the DNp44 descending neuron might represent the shortest known *Drosophila* brain circuit from sensory periphery to descending motor control (two synapses). It could mediate a “reflex”-like escape response to dryness, as *Drosophila*, like all invertebrates, are particularly sensitive to desiccation through cuticular evaporation.

## Discussion

Neural circuits for temperature and humidity are vital for survival and reproduction, but they are much less well-characterized than the olfactory system, with which they share many anatomical features. Thermo- and hygrosensory RNs in *Drosophila* are housed in the antennae, innervate dedicated glomeruli in the AL, and target stereotyped projection neurons that relay information to higher brain centers to mediate innate and learned behaviors [48]. By reconstructing individual neurons and their synapses in a whole-brain EM volume, we have identified novel primary, secondary, and tertiary neurons of the thermo- and hygrosensory systems.

We reconstructed sensory neurons in the five known VP glomeruli and two novel ones. Surprisingly, the average number per VP glomerulus was only approximately one-quarter that of olfactory glomeruli [49]. Our VP1d most likely corresponds to the glomerulus originally designated VP1, which may represent evaporative cooling. We suggest, based on Ir receptor expression, that VP1l might represent cooling, while VP1m might represent humidity, with confirmation awaiting future physiological and behavioral experiments.

We reconstructed 89 VP PNs (comprising 38 morphological types, 23 completely novel) connecting VP glomeruli to higher brain centers; further studies will be necessary to define their valences and functions. Many stereotyped VP PNs received substantial input from more than one glomerulus, typically additional VP glomeruli but also VC5; we report 13 VC5 PNs (Table 1) and speculate that VC5 represents temperature and/or humidity directly or olfactory inputs especially sensitive to temperature and/or humidity. Biglomerular PNs generally innervated two glomeruli of the same predicted modality (e.g., VP1l+VP3) or paired humidity with temperature (e.g., VP5+VP2). Integration of antagonistic inputs (e.g., heating and cooling or dry and humid) was more characteristic of third-order neurons.

The lACA had previously been identified as a mushroom body accessory calyx targeted by the slow-cool VP3 vPN [27]. We found that several other VP PNs target this structure, most notably the VP2 adPN, as does the VC5+ adPN, again hinting that this glomerulus might be thermo- or hygrosensory. Unexpectedly, both Kenyon cells and other target neurons generally received more input from lACA-innervating PNs outside of the lACA neuropil than inside it. We therefore speculate that one important role of the lACA is to permit modulation of thermo- and hygrosensory PNs and targets by the slow-cool VP3 vPN en route to other neuropils.

By characterizing the connectome of the RHS lACA, we identified three α’/β’ KCs specialized for thermosensation and 24 others that integrated thermosensory with olfactory and/or hygrosensory information. The two unique lACA-associated KCγ neurons exhibited highly branched axons that wrapped around the γ lobe instead of projecting to its dorsal tip like those of KCγds [36]. Future analysis of downstream partners should reveal whether these connect to distinct subsets of dopaminergic neurons (DANs) and/or mushroom body output neurons (MBONs) to influence memory formation or retrieval.

We also identified 19 novel classes of lACA-associated neurons, most of which connect not to KCs, but rather to each other, to lACA PNs, and/or to as-yet unidentified neurons in other neuropils. Three of the lACA network’s main output neuropils are the LH, SMP, and antlers, all previously associated with olfactory responses [50]; in particular, the SMP features both DAN inputs and MBON outputs [39]. Indeed, two neurons had previously been identified as downstream targets of DA2 PNs, an aversive olfactory channel [51]. However, the specific downstream partners of these lACA output neurons have yet to be identified.

We reconstructed two putative DN1a neurons [43] and a third neuron that was morphologically similar but upstream of them (Figure 5). DN1 neurons have been reported to entrain the circadian clock to temperature in the absence of light signals, firing in the 20°C–29°C temperature range [52]. We observed DN1 innervation by both cooling- and heating-responsive VP PNs (Figures 5L and 6G), which could reflect sensitivity to temperature fluctuations at higher temperature ranges for adjusting temperature preferences more dynamically.

We found that while distinct subsets of thermo- and hygrosensory inputs were spatially segregated in the AL, they were integrated with each other and with olfactory inputs deeper in the pathway. It seems reasonable to hypothesize that the rate of encountering odor molecules changes with varying temperature and humidity, requiring some knowledge of context for accurate odor perception. It is also plausible that antennal thermo- and hygrosensory circuits are more ancient, with neurons for detecting, remembering, and responding to specific volatile chemicals evolving later.

While this study has significantly improved our picture of thermo- and hygrosensory circuitry, many gaps remain at the level of third-order target neurons in higher brain areas. Given the time-consuming labor of manual reconstruction, our efforts were focused on the lACA. Taking full advantage of whole EM volumes to elucidate the organization of brainwide circuits, and making comparisons between animals to assess natural and experimental variation, will require rapid and accurate automated segmentation of neuronal profiles and their synapses [53]. This study should provide a lasting platform for this next stage of exploration and the functional studies that will accompany them.

## STAR★Methods

### Key Resources Table

**Table:** 

REAGENT or RESOURCE	SOURCE	IDENTIFIER
**Antibodies**		

mouse anti-nc82 (1:200)	DSHB, University of Iowa	Bruchpilot; RRID:AB_2314866
chicken anti-GFP (1:100)	Avēs Labs Inc.	Cat#: GFP-1010; RRID:AB_10000240
rabbit anti-DsRED (1:200)	Takara Bio	Cat#: 632392; RRID:AB_10013483
goat anti-mouse Cy5 (1:200)	Jackson ImmunoResearch Labs	Cat#: 115-175-166; RRID:AB_2338714
goat anti-chicken 488 (1:200)	Invitrogen	Cat#: A-11039; RRID:AB_142924
goat anti-rabbit Cy3 (1:200)	Jackson ImmunoResearch Labs	Cat#: #115-165-166; RRID:AB_2338692

**Experimental Models: Organisms/Strains**		

Ir40a-LexA	[25]	N/A
Gr28b.d-Gal4	[54]	N/A
UAS-myr:GFP (P[10UAS-IVS-myr::GFP]attP1)	[55]	N/A
LexAop-mRFP	Bloomington Stock Center	Stock# 29956; RRID:BDSC_29956
Ir21a-T2A-Gal4	This paper	N/A
Ir68a-T2A-Gal4	This paper	N/A
yw, 3XP3-EGFP, Vasa-Cas9 attp18	Kate Koles, Rodal Lab Brandeis University	N/A

**Oligonucleotides**		

gRNA against Ir21a intronic region: GCGCGTGAGTATTGCTTAAT	This paper	N/A
gRNA against Ir68a intronic region: GTGTGATATGAAAAGCATTC	This paper	N/A
Primer 1: Ir21a 5′ homology arm: atacgactcactatagggcgacccaccggtCGATATG TCATATTATTGGGTAGCTCTGGT	This paper	N/A
Primer 2: Ir21a 5′ homology arm: ccgaaaaccgcttctgacctggggcggccgcAA GCAATACTCACGCGCAT	This paper	N/A
Primer 3: Ir21a 3′ homology arm: ccgaaaaccgcttctgacctgggggcgcgccAATAGG GATACGTTTTTGTAACAAATAATGCGCTT CACACAGGAG	This paper	N/A
Primer 4: Ir21a 3′ homology arm: aagggaacctccccactagtggtaccCTCAAATGA AGCGCCGATCGG	This paper	N/A
Primer 5: Ir68a 5′ homology arm: atacgactcactatagggcgacccaccggtGGAAC CTCTTTGCCAGTTGCC	This paper	N/A
Primer 6: Ir68a 5′ homology arm: ccgaaaaccgcttctgacctggggcggccgcTGCTT TTCATATCACACTAGATTATTTTG	This paper	N/A
Primer 7: Ir68a 3′ homology arm: ccgaaaaccgcttctgacctgggggcgcgccTTCC GGCGACTTAATGGCTTTGT	This paper	N/A
Primer 8: Ir68a 3′ homology arm: aagggaacctccccactagtggtaccCTTCTAAAG AGATGGCCAAGCAAAAGC	This paper	N/A

**Recombinant DNA**		

pCFD3-dU6:3gRNA	[56]	N/A
pGEM (phase 0)	[57]	N/A

**Deposited Data**		

The Full Adult Fly Brain ssTEM dataset	[30]	https://temca2data.org/
FAFB manual neuronal reconstructions	This paper [30, 33, 51, 58, 59],	https://fafb.catmaid.virtualflybrain.org/ Neurons can be retrieved by their SKID (skeleton identifier).
Data S1 Skeletons and meshes	This paper [30, 33, 51, 58, 59],	https://doi.org/10.5281/zenodo.3879033
FlyCircuit 1.2 database of *Drosophila* brain neurons	[60]	http://www.flycircuit.tw/
Fly Light GAL4/LexA collection	[26]	http://flweb.janelia.org/cgi-bin/flew.cgi

**Software and Algorithms**		

CATMAID	[61, 62]	https://github.com/catmaid/CATMAID
natverse, including the nat, nat.flybrains, elmr, tracerutils, flycircuit, and rcatmaid packages	[63]	https://github.com/natverse/
NBLAST algorithm and R package	[31, 63]	https://github.com/natverse/nat.nblast
Pymaid, python code for interacting with CATMAID	[33]	https://github.com/schlegelp/pymaid
FAFBseg, python code for working with the partial autosegmentation of FAFB from [53]	[33]	https://github.com/flyconnectome/fafbseg-py
CATMAID-to-Blender	[64]	https://github.com/schlegelp/CATMAID-to-Blender
rmushroom package	[30]	https://github.com/flyconnectome/rmushroom

### Resource Availability

#### Lead Contact

All queries regarding EM reconstructions, software and algorithms should be directed to the Lead Contact, Gregory Jefferis (jefferis@mrc-lmb.cam.ac.uk).

#### Materials Availability

All plasmids and fly strains generated in this study are freely available upon request. Please contact Paul A. Garrity (pgarrity@brandeis.edu).

#### Data and Code Availability

All neuron reconstructions described in this study will be uploaded to a public CATMAID instance hosted by Virtual Fly Brain (https://fafb.catmaid.virtualflybrain.org/) following publication. Additionally, the skeletons and meshes have been deposited in Zenodo: https://doi.org/10.5281/zenodo.3879033. The full source code and documentation for natverse packages is available at https://github.com/natverse/ and http://natverse.org/.

### Experimental Model and Subject Details

Standard *Drosophila* husbandry techniques were used for the maintenance and propagation of flies. For immunohistochemistry, flies were raised at 25°C on standard food.

### Method Details

#### *Drosophila* Strains and Driver Line Generation

Prior to this publication, Ir21a- and Ir68a-Gal4 drivers were available, but neither one portrayed complete expression pattern of their respective genes as these lines were made by making a guess about what sequences that might comprise a gene’s regulatory region (e.g., 5′ sequences) and placing those sequences upstream of Gal4. The Ir21a(putative promoter)-Gal4 expression in the larva was described previously [65]. Unfortunately, that construct lacks key regulatory information and does not express in the adult. The construction and expression of the Ir68a(putative promoter)-Gal4 was also previously described [20]. That Gal4 drives very weak expression in the adult and can only be detected in sacculus chamber II. For these reasons we sought to generate new drivers using the T2A-Gal4 knock-in strategy that would provide a more complete view of these genes’ expression patterns.

Ir21a-T2A-Gal4 and Ir68a-T2A-Gal4 were generated using techniques previously described [57]. Briefly, intronic regions in the Ir21a and Ir68a genes were targeted via homology directed repair by injecting two plasmids, one containing gRNA (Ir21a: 5′-GCGCGTGAGTATTGCTTAAT; Ir68a: 5′-GTGTGATATGAAAAGCATTC) and another with T2A-Gal4 3XP3-RFP at phase0 (flanked by homology arms; Ir21a: 5′arm primers 1-2, 3′arm primers 3-4; Ir68a: 5′arm: primers 5-6, 3′arm primers 7-8) into embryos carrying Vasa-Cas9 3XP3-EGFP (kindly shared by Kate Koles and Rodal Lab, Brandeis University).

#### Immunohistochemistry

For brain dissections, whole flies were fixed for 2 h in 4% paraformaldehyde while rotating and washed several times prior to dissections. The dissected brains were incubated at room temperature in blocking solution (10% normal goat serum) for one h. Primary and secondary antibody incubations were done at 4°C for 48 h each. Washing between antibody incubations was performed over 24 h at 4°C.

Third antennal sections were dissected, fixed in 4% paraformaldehyde for 1 h on ice, and incubated in blocking solution for 1 h at room temperature. Primary and secondary antibody incubations were done at 4°C for 24 h each while rotating. Washing between antibody incubations were performed at room temperature 3-4 times with PBS-Tx (PBS with 0.5% Triton X).

#### IHC Image Acquisition

Imaging of antennae and the antennal lobe were performed using a Zeiss LSM 880 confocal microscope with a 63X oil lens. Maximum intensity z stack projections were made from z stack sections taken every 1μm.

#### Sparse EM Reconstruction

Neuron skeletons were traced in a full adult female *Drosophila* brain ssTEM (serial section transmission electron microscope) volume (FAFB, https://fafb.catmaid.virtualflybrain.org/, http://temca2data.org), either manually as previously reported [30] or by automated segmentation, using a modified version of CATMAID (http://www.catmaid.org) [61, 62]. Skeleton fragments that had been automatically segmented with the flood-filling technique [53] were manually joined in a separate CATMAID instance, imported into the manual CATMAID instance, and merged with manually traced partial skeletons where possible (https://github.com/flyconnectome/fafbseg-py). Skeletons were either traced to identification (with soma, backbone, and main branches) or to completion (with all twigs, including pre- and postsynapses, and fully reviewed) [33].

Chemical synapses were annotated based on previously described criteria: thick, dark active zone, presynaptic membrane specializations (T-bars, vesicles), and a synaptic cleft [66]. We scored each continuous synaptic cleft as a single presynapse and opposing neuronal membranes in contact with that synaptic cleft as associated postsynapses.

#### Analysis and Representation of Traced Skeletons

R packages from the natverse collection (http://natverse.org/) [63] were used to plot traced skeletons and analyze their morphology. Custom R and Python scripts were used to perform additional analysis, as described below.

#### Neuron Clustering via NBLAST

The nat.nblast R package (https://github.com/natverse/nat.nblast) was used to compare neuron skeletons by morphology and position and generate a hierarchical clustering with Ward’s method of the neurons within each group (e.g., VP PNs), providing a method for identifying neuron types [31].

#### Light versus EM Comparisons of Neuron Morphology

To assist in the identification of our reconstructed neurons, we used nat.nblast to compare them with segmentations of annotated PNs in the light-level FlyCircuit database [31, 60] (http://www.flycircuit.tw/). We used linear and then non-rigid transformations to bring neurons from FAFB into FCWB (FlyCircuit whole brain) space, then performed an all-by-all NBLAST to look for the closest matches. Neurons that did not have clear matches in FlyCircuit could sometimes be manually identified by comparing morphology to published data. Sparse driver lines predicted to label our reconstructed neurons were identified in a similar way through comparisons with the Fly Light database (http://flweb.janelia.org/cgi-bin/flew.cgi) [26].

#### Neuronal Nomenclature

Sensory neurons were typed by the AL glomerulus they innervated. Projection neurons were typed according to their AL glomerular innervation, AL tract, and neuroblast lineage [33, 37] and their likely neurotransmitter types assigned by tract [33, 36]. Kenyon cells were typed according to their positions in the peduncle and the MB axon lobes they innervated [39]. Lateral horn neurons were typed based on their primary neurite tracts and areas of innervation, according to previously established conventions [67]. Numbers were added to names with # when multiple individuals were assigned the same type.

### Quantification and Statistical Analysis

#### Analysis of Biglomerular PN Inputs

A best fit line to a linear regression model showing correlation of cable length in each glomerulus with RN input was plotted in R using the lm() function. This function provided the Multiple R-squared and p values (Figure 3H).

#### Analysis of lACA Circuit Connectivity

Meshes were made in Blender from the completed axons and synapses of the VP3 vPN and VP2 adPN, which ramify throughout the lACA, and imported into CATMAID as lACA volumes. All PNs with presynapses in these lACA volumes were identified with the CATMAID connectivity widget and designated as lACA PNs. We manually annotated all postsynapses downstream of these VP3 vPN and VP2 adPN, which together contribute > 90% of lACA presynapses. These downstream postynapses were then randomly sampled and the corresponding skeletons reconstructed to identification. We continued sampling until the 10 most recent novel hits featured fewer than five connections with the query neuron (Figures S5D–S5D’’), corresponding to 21 - 30% of postsynapses for each PN. All identified neurons were reconstructed in the lACA, and those with at least 5 connections to lACA PNs were designated as lACA circuit neurons. We reconstructed 42 sampled neurons within the lACA to determine their inputs, then completely reconstructed 20 (47.6%) of these, including representatives of all morphological types receiving more than 15 lACA PN inputs. Input PNs and target neurons of the same type were pooled, and adjacency matrices were constructed in CATMAID using the Graph widget. For simplicity, a few neurons in the adjacency matrices were omitted from the circuit diagrams (Figures 5L and 5M).

#### Analysis of lACA-associated Kenyon Cell Inputs

The dendrites of KCs that shared 5 or more synapses with lACA-associated PNs within the lACA volume on the RHS were reconstructed in CATMAID, and their postsynapses were manually associated with upstream neurons - mainly antennal lobe PNs which had already been traced to identification [30, 33]. Analysis of KC inputs was performed in R with a customised script, utilizing the nat and elmr packages from the natverse. KC skeletons were bridged from FAFB space into the JFRC2 template [26] (http://www.virtualflybrain.org/) using the nat.flybrains package (natverse) and then pruned with the split_neuron_local() function in the tracerutils package (https://flyconnectome.github.io/tracerutils/) to include only dendrites (branches off the main tract in the calyx). For each KC branch, all upstream PNs contributing at least 5 synapses were identified and weighted equally as inputs observed. (Non-PN partners and inputs from lACA-associated PNs within the lACA volume were omitted from further analysis.)

Expected numbers of inputs representing each AL glomerulus were based on the total number of boutons reported [30], or newly identified, for each PN class. PN boutons were manually annotated and extracted using the rmushroom package (https://github.com/zhihaozheng). A virtual KC population with the same number of KCs and PN inputs was created by random draw, with the probability of each input based on the percentage share of associated PN boutons. This was repeated 1000 times to generate an average number of expected inputs for the population (Figures 4L and 4M). Inputs from each glomerulus were assigned a putative valence based on the olfactory literature [33].

## Supplemental Information

Document S1. Figures S1–S6 and Tables S1–S3

Table S4. Marin 2020 All Neurons, Related to STAR Methods Table of all reconstructed skeletons in this study that have been deposited in public repository https://fafb.catmaid.virtualflybrain.org/. SKID: skeleton identifier. NAME: name assigned in this paper. TYPE: broad neuron type. PREV_RELEASED: whether the skeleton, or an earlier version of it, was published in a previous study. PAPER: other paper(s) in which the skeleton has been published, if applicable. BEST_FIB: neuron identifier of the best match for this skid in the hemibrain:v1.1 data set. NAME_FIB: the instance assigned to that neuron in neuPrint, https://neuprint.janelia.org/.

Table S5. lACA Presynapses, Related to Figure 4 Table generated by the CATMAID connectivity widget showing all presynapses within the lACA volume, with all PNs identified and total and percentage of inputs contributed by each PN. Skeleton ID: reconstruction in CATMAID. Name: name assigned in this paper. Connector ID: connector ID in CATMAID. Relation: presynapse. Percentage: percentage of PN presynapses in lACA contributed by PN. Total: total presynapses in lACA contributed by PN.

Table S6. lACA PN Targets, Related to Figures 4 and 5 Table generated by the CATMAID connectivity widget showing all neurons receiving at least five connections from lACA PNs within the lACA volume. Downstream neuron: skeleton ID of postsynaptic neuron. Sum: total annotated connections downstream of lACA PNs. #Synapses with XXXXX: inputs from a particular presynaptic lACA PN. Node count: number of nodes (reflecting size) of postsynaptic fragment.

Data S1. Skeletons and Meshes, Related to STAR Methods *marin2020-skeletons* contains files describing the morphology of each neuron in this study (in FAFB14 space, .swc format). *glomeruli_meshes* contains the glomerular meshes (in FAFB14 space, .stl format). *neuropil_meshes* contains the glomerular meshes (in FAFB14 space, .stl format).

Document S2. Article plus Supplemental Information
