# Supplementary material for: Connectomics Analysis Reveals First-, Second-, and Third-Order Thermosensory and Hygrosensory Neurons in the Adult Drosophila Brain
Source: Curr Biol. 2020 Aug 17;30(16):3167–3182.e4. doi: 10.1016/j.cub.2020.06.028 (PMC7443704; doi:10.1016/j.cub.2020.06.028)
Supplement: Document S1. Figures S1–S6 and Tables S1–S3 [file mmc1.pdf]

Current Biology, Volume *30*

Supplemental Information

Connectomics Analysis Reveals First-, Second-, and Third-Order Thermosensory and Hygrosensory Neurons in the Adult *Drosophila* Brain

Elizabeth C. Marin, Laurin Büld, Maria Theiss, Tatevik Sarkissian, Ruairí J.V. Roberts, Robert Turnbull, Imaan F.M. Tamimi, Markus W. Pleijzier, Willem J. Laursen, Nik Drummond, Philipp Schlegel, Alexander S. Bates, Feng Li, Matthias Landgraf, Marta Costa, Davi D. Bock, Paul A. Garrity, and Gregory S.X.E. Jefferis

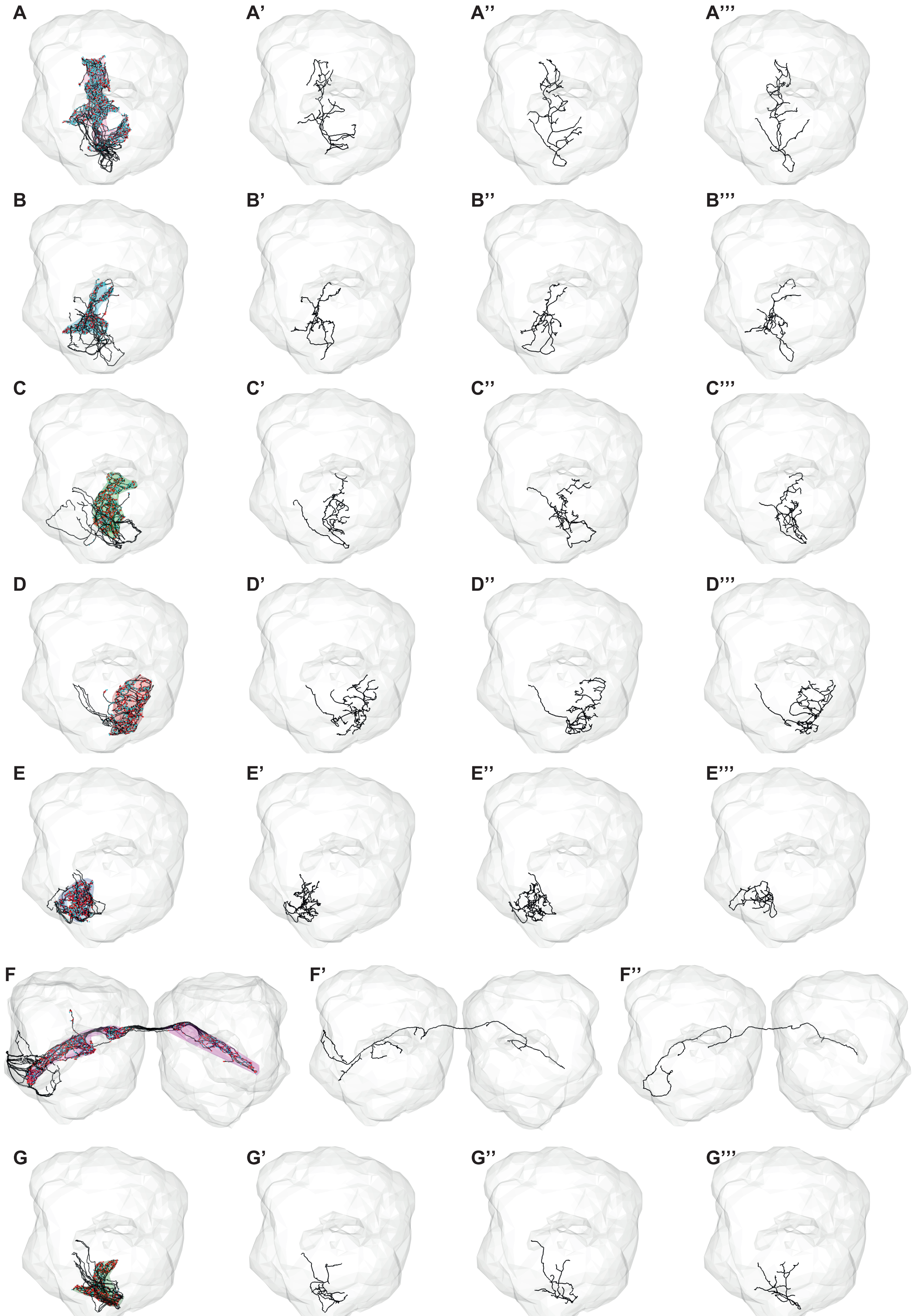
A
A'
A''
A'''
B
B'
B''
B'''
C
C'
C''
C'''
D
D'
D''
D'''
E
E'
E''
E'''
F
F'
F''
G
G'
G''
G'''

Figure S1. Individual reconstructions reveal morphological stereotypy for seven distinct classes of VP sensory neurons, related to Figure 1. Right antennal lobe, frontal view. Reconstructed neurons in black, with presynaptic sites in red and postsynaptic sites in cyan. **A-A'''.** Full population (9) and three completed examples of VP1d RNs. **B-B'''.** Full population (6) and three completed examples of VP1l RNs. **C-C'''.** Full population (6) and three completed examples of VP1m RNs. **D-D'''.** Full population (3) and three completed examples of VP2 RNs. **E-E'''.** Full population (4) and three completed examples of VP3 RNs. **E'''** depicts VP3RNbr#1 (the simpler, suspected non-aristal, RN). **F-F'''.** Full population (14) and two completed examples of VP4 RNs. **G-G'''.** Full population (9) and three completed examples of VP5 RNs.

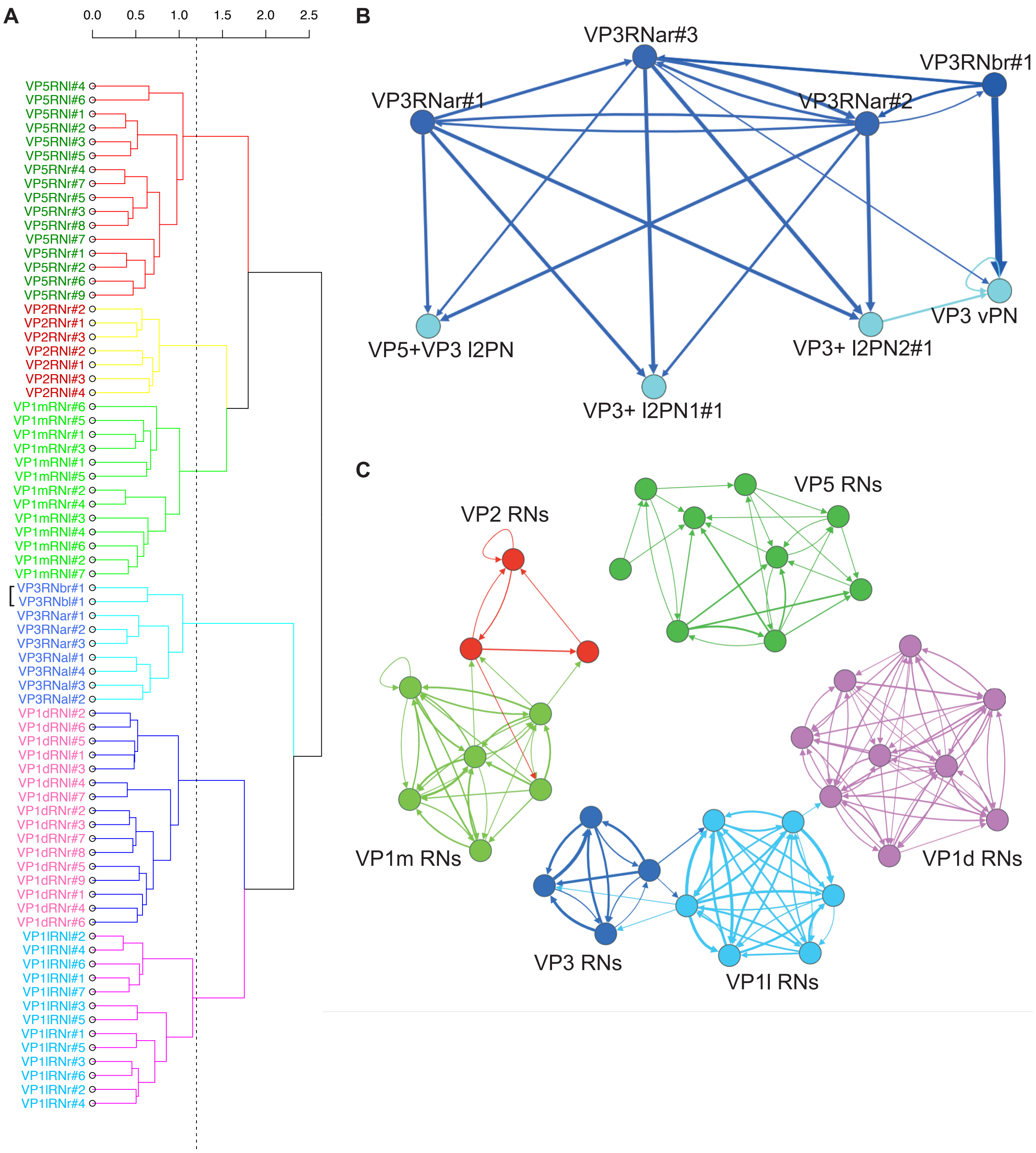

A
0.0
0.5
1.0
1.5
2.0
2.5
VP5RNl#4
VP5RNl#6
VP5RNl#1
VP5RNl#2
VP5RNl#3
VP5RNl#5
VP5RNr#4
VP5RNr#7
VP5RNr#5
VP5RNr#3
VP5RNr#8
VP5RNl#7
VP5RNr#1
VP5RNr#2
VP5RNr#6
VP5RNr#9
VP2RNr#2
VP2RNr#1
VP2RNr#3
VP2RNl#2
VP2RNl#1
VP2RNl#3
VP2RNl#4
VP1mRNr#6
VP1mRNr#5
VP1mRNr#1
VP1mRNr#3
VP1mRNl#1
VP1mRNl#5
VP1mRNr#2
VP1mRNr#4
VP1mRNl#3
VP1mRNl#4
VP1mRNl#6
VP1mRNl#2
VP1mRNl#7
VP3RNbr#1
VP3RNbl#1
VP3RNar#1
VP3RNar#2
VP3RNar#3
VP3RNal#1
VP3RNal#4
VP3RNal#3
VP3RNal#2
VP1dRNl#2
VP1dRNl#6
VP1dRNl#5
VP1dRNl#1
VP1dRNl#3
VP1dRNl#4
VP1dRNl#7
VP1dRNr#2
VP1dRNr#3
VP1dRNr#7
VP1dRNr#8
VP1dRNr#5
VP1dRNr#9
VP1dRNr#1
VP1dRNr#4
VP1dRNr#6
VP1lRNl#2
VP1lRNl#4
VP1lRNl#6
VP1lRNl#1
VP1lRNl#7
VP1lRNl#3
VP1lRNl#5
VP1lRNr#1
VP1lRNr#5
VP1lRNr#3
VP1lRNr#6
VP1lRNr#2
VP1lRNr#4
B
VP3RNar#3
VP3RNbr#1
VP3RNar#1
VP3RNar#2
VP3 vPN
VP5+VP3 I2PN
VP3+ I2PN2#1
VP3+ I2PN1#1
C
VP5 RNs
VP2 RNs
VP1m RNs
VP1d RNs
VP3 RNs
VP1l RNs

Figure S2. There are seven distinct populations of thermosensory and hygrosensory neurons, related to Figure 1. A. Morphological hierarchical clustering of the unilateral sensory neurons from both hemispheres. Cut at height 1.2 (dashed line) which groups the RNs by type. Neuron names are colour-coded by glomerulus. Bracket: simple VP3 TRNs. **B-C.** Connectivity graphs for thermo- and hygrosensory neuron populations. Arrow directions and thicknesses reflect direction of information flow and numbers of synaptic connections. Each circle (node) represents one neuron, colour-coded by class. **B.** The simpler VP3 TRN (VP3RNbr#1) provides input to the "slow-cool" VP3 vPN but not to VP5+VP3 lPN, VP3+ lPN1#1, or VP3+ lPN2#1. VP3RNar#1-3: remaining three (putative aristal) VP3 TRNs. Edges of <3 connections have been omitted for visual clarity. **C.** VP1d, VP1l, and VP1m sensory neurons constitute three separate populations.

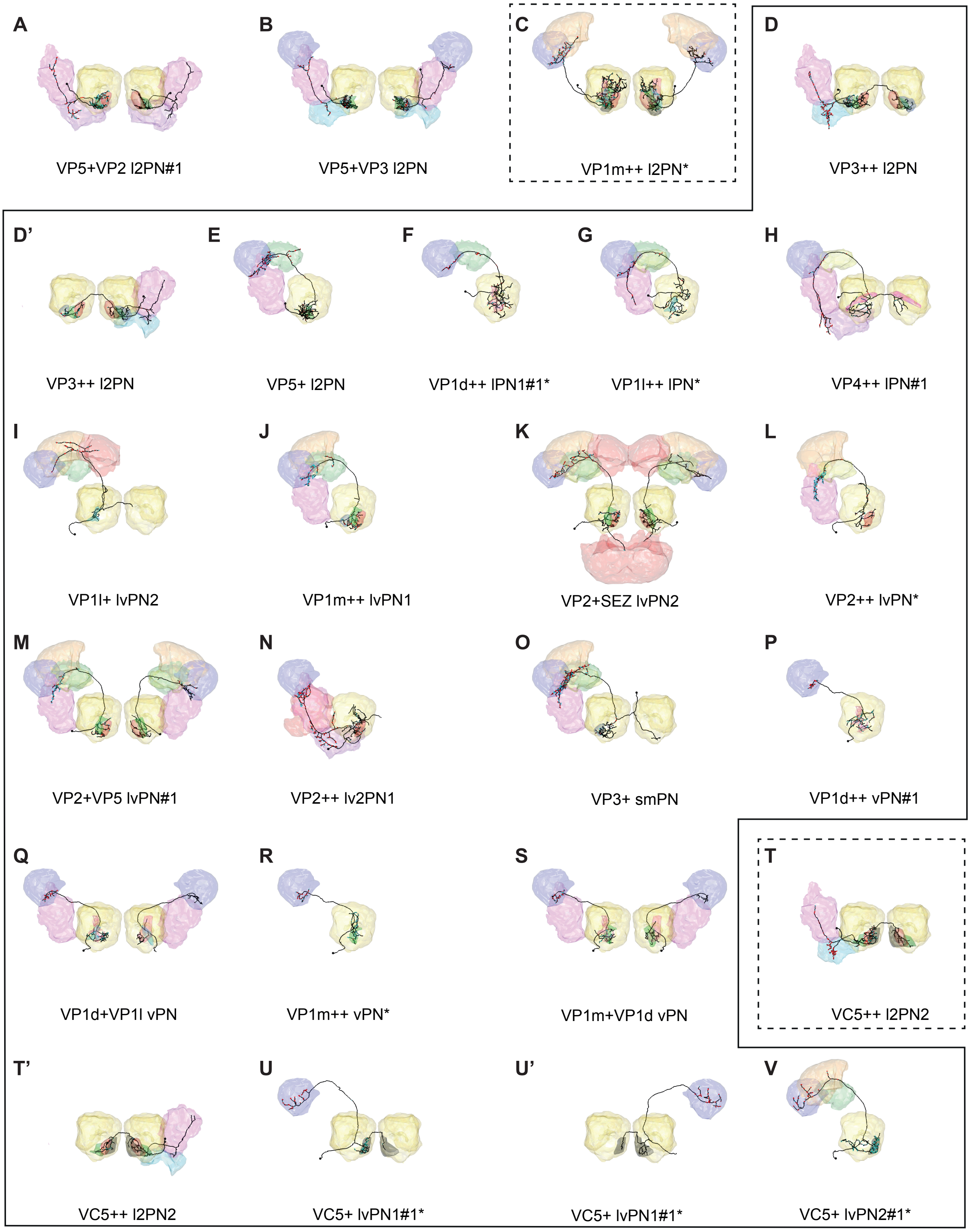
A
VP5+VP2 l2PN#1
B
VP5+VP3 l2PN
C
VP1m++ l2PN*
D
VP3++ l2PN
D'
VP3++ l2PN
E
VP5+ l2PN
F
VP1d++ lPN1#1*
G
VP1l++ lPN*
H
VP4++ lPN#1
I
VP1l+ lvPN2
J
VP1m++ lvPN1
K
VP2+SEZ lvPN2
L
VP2++ lvPN*
M
VP2+VP5 lvPN#1
N
VP2++ lv2PN1
O
VP3+ smPN
P
VP1d++ vPN#1
Q
VP1d+VP1l vPN
R
VP1m++ vPN*
S
VP1m+VP1d vPN
T
VC5++ l2PN2
T'
VC5++ l2PN2
U
VC5+ lvPN1#1*
U'
VC5+ lvPN1#1*
V
VC5+ lvPN2#1*

Figure S3. Additional types of putative thermo- and hygrosensory PNs, related to Table 1. Frontal views of reconstructions (black) of additional candidate thermo- and hygrosensory PNs that innervate VP glomeruli (colour-coded as in Figure 1B-B’, non-VP glomeruli in grey). Presynaptic sites in red and postsynaptic sites in cyan. Brain neuropils colour-coded as in Figure 2C. Asterisks: primarily olfactory PNs. Black outline: candidate novel thermosensory PNs. Dashed outline: PNs previously described but not recognised as VP/thermosensory, or associated with the wrong glomeruli.

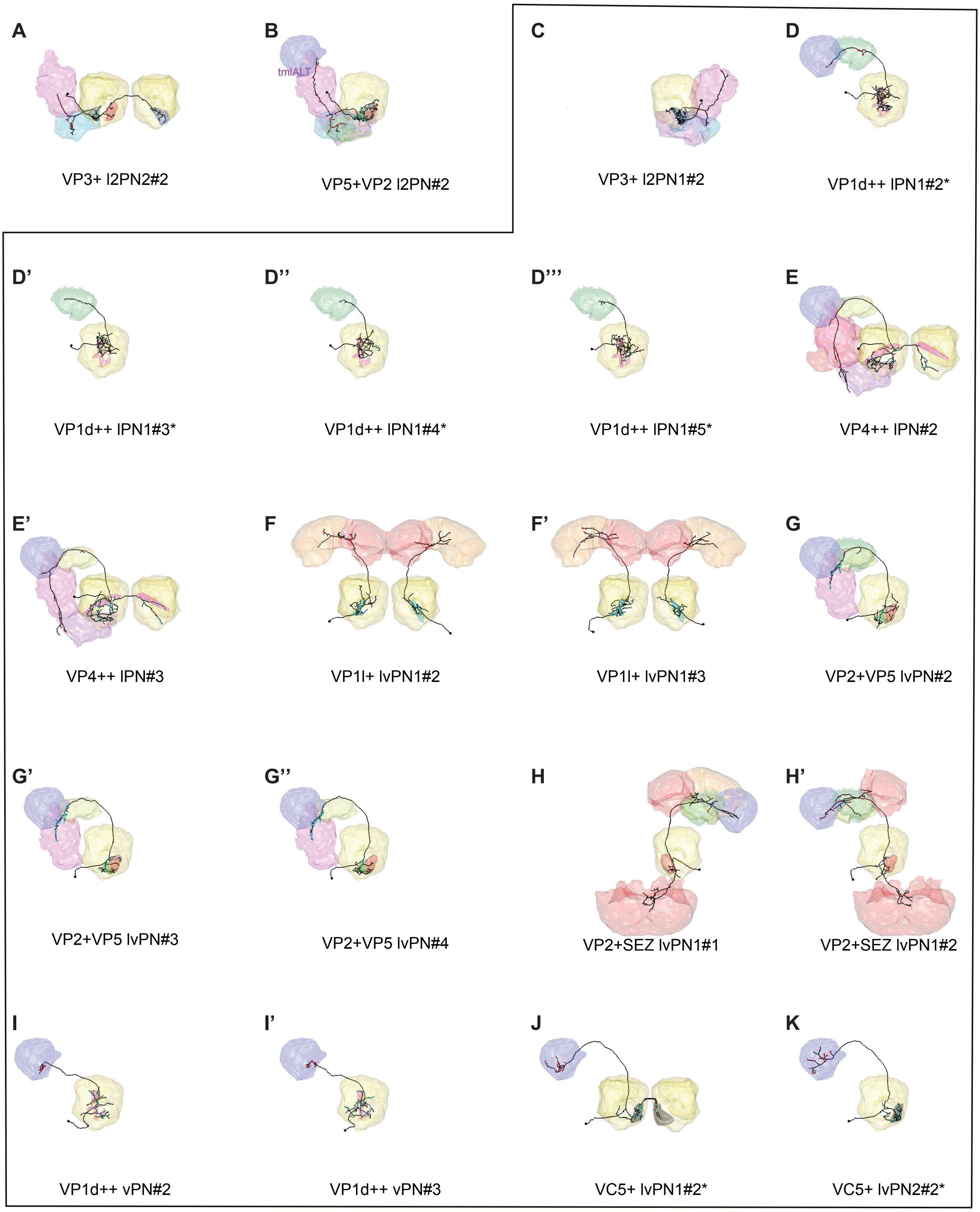

A
VP3+ l2PN2#2
B
tmlALT
VP5+VP2 l2PN#2
C
VP3+ l2PN1#2
D
VP1d++ lPN1#2*
D'
VP1d++ lPN1#3*
D''
VP1d++ lPN1#4*
D'''
VP1d++ lPN1#5*
E
VP4++ lPN#2
E'
VP4++ lPN#3
F
VP1l+ lvPN1#2
F'
VP1l+ lvPN1#3
G
VP2+VP5 lvPN#2
G'
VP2+VP5 lvPN#3
G''
VP2+VP5 lvPN#4
H
VP2+SEZ lvPN1#1
H'
VP2+SEZ lvPN1#2
I
VP1d++ vPN#2
I'
VP1d++ vPN#3
J
VC5+ lvPN1#2*
K
VC5+ lvPN2#2*

Figure S4. Extra examples of putative thermo- and hygrosensory PN types, related to Table 1. Frontal views of reconstructions (black) of additional candidate thermo- and hygrosensory PNs that innervate VP glomeruli (colour-coded as in Figure 1B-B', non-VP glomeruli in grey). Presynaptic sites in red and postsynaptic sites in cyan. Brain neuropils colour-coded as in Figure 2C. Asterisks: primarily olfactory PNs. Black outline: candidate novel thermosensory PNs.

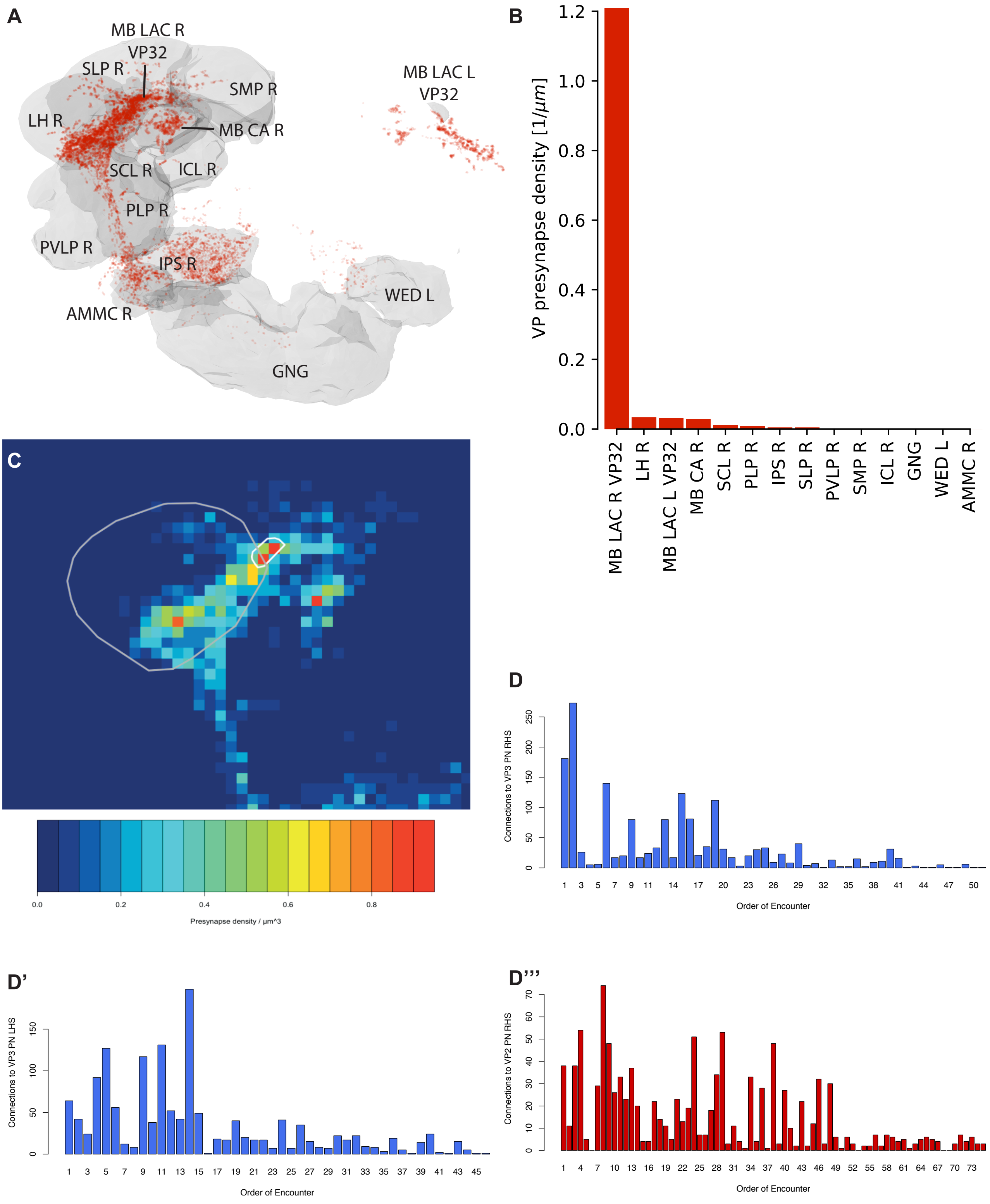
A
MB LAC R
VP32
SLP R
SMP R
LH R
MB CA R
SCL R
ICL R
PLP R
PVLP R
IPS R
AMMC R
WED L
GNG
MB LAC L
VP32
B
VP presynapse density [1/μm]
MB LAC R VP32
LH R
MB LAC L VP32
MB CA R
SCL R
PLP R
IPS R
SLP R
PVLP R
SMP R
ICL R
GNG
WED L
AMMC R
C
Presynapse density / µm^3
D
Connections to VP3 PN RHS
Order of Encounter
D'
Connections to VP3 PN LHS
Order of Encounter
D'''
Connections to VP2 PN RHS
Order of Encounter

Figure S5. Random sampling to define circuitry in the lACA, the neuropil most densely targeted by VP PNs, related to Figure 4. A. Presynapses of all RHS AL VP PNs, plotted with neuropil volumes. AMMC R: RHS antennal mechanosensory and motor centre. MB CA R: RHS mushroom body calyx. ICL R: RHS inferior clamp. IPS R: RHS inferior posterior slope. LH: RHS lateral horn. MB LAC R VP32: RHS lateral accessory calyx. MB LAC L VP32: LHS lateral accessory calyx. PLP R: RHS posterior lateral protocerebrum. PVLP R: RHS posterior ventral protocerebrum. SCL R: RHS superior clamp. GNG: suboesophageal zone. SLP R: RHS superior lateral protocerebrum. SMP R: RHS superior medial protocerebrum. WED L: LHS wedge. **B.** VP PN presynapse density plotted in each neuropil (abbreviations as in A). **C.** VP PN presynapse density (presynapses/ cubic micron), with LH and lACA outlined in white. **D-D''.** Histograms depicting connection strength of recovered novel hits from successive sampling of randomised postsynaptic profiles downstream of the RHS and LHS VP3 PN and the RHS VP2 PN in the lACA.

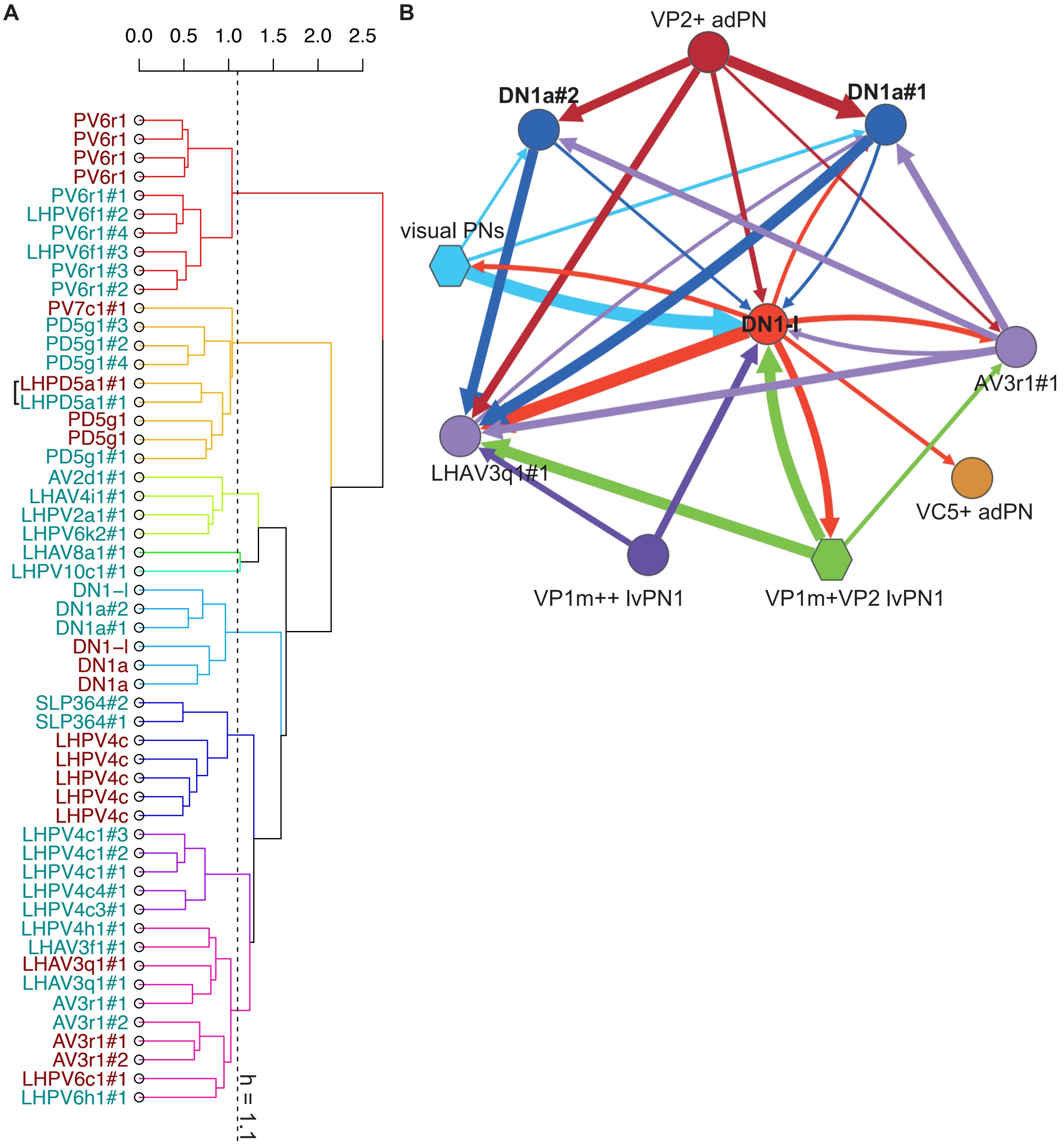

A
0.0 0.5 1.0 1.5 2.0 2.5
PV6r1
PV6r1
PV6r1
PV6r1
PV6r1#1
LHPV6f1#2
PV6r1#4
LHPV6f1#3
PV6r1#3
PV6r1#2
PV7c1#1
PD5g1#3
PD5g1#2
PD5g1#4
LHPD5a1#1
LHPD5a1#1
PD5g1
PD5g1
PD5g1#1
AV2d1#1
LHAV4i1#1
LHPV2a1#1
LHPV6k2#1
LHAV8a1#1
LHPV10c1#1
DN1−l
DN1a#2
DN1a#1
DN1−l
DN1a
DN1a
SLP364#2
SLP364#1
LHPV4c
LHPV4c
LHPV4c
LHPV4c
LHPV4c
LHPV4c1#3
LHPV4c1#2
LHPV4c1#1
LHPV4c4#1
LHPV4c3#1
LHPV4h1#1
LHAV3f1#1
LHAV3q1#1
LHAV3q1#1
AV3r1#1
AV3r1#2
AV3r1#1
AV3r1#2
LHPV6c1#1
LHPV6h1#1
h = 1.1
B
VP2+ adPN
DN1a#2
DN1a#1
visual PNs
DN1-l
AV3r1#1
LHAV3q1#1
VC5+ adPN
VP1m++ lvPN1
VP1m+VP2 lvPN1

Figure S6. Non-Kenyon cell IACA circuit neuron stereotypy and connectivity, related to Figure 5. A. Morphological hierarchical clustering of non-KC IACA neurons using NBLAST. Cut at height 1.1 (dashed line) to retrieve groups by neuron type. Dark cyan: RHS targets. Dark red: LHS targets. Square brackets: unique neurons matched across hemispheres. **B.** Connectivity graph showing that DN1a#1 and DN1a#2 have similar connectivity, distinct from that of DN1-l. Each circle (node) represents one neuron and each hexagon represents multiple pooled neurons of the same modality. Arrow directions and thicknesses reflect direction of information flow and numbers of synaptic connections. Edges of <10 connections and the VP3 vPN and VP2 adPN have been omitted for clarity.

SKID	NAME	SIDE	VP3	VP2	LACA	LP	VP	OTHER
5816884	KCg-s2R	RHS	138	48	187	9	11	AV3f2#1, DN1-l, mPN, visual
4181431	tKCa'b'R#1	RHS	26	48	75	0	0	NA
5824701	tKCa'b'R#2	RHS	33	34	67	0	0	NA
6279367	tKCa'b'R#3	RHS	31	32	63	0	0	NA
8799774	KCa'b'R#1	RHS	31	22	53	0	0	VL2p
4182914	KCa'b'R#2	RHS	23	38	61	0	0	DC1
8139894	KCa'b'R#3	RHS	24	30	57	0	20	NA
5824641	KCa'b'R#4	RHS	15	38	53	0	0	V, DL2d
5832021	KCa'b'R#5	RHS	16	26	43	16	0	(DA4l)
8821288	KCa'b'R#6	RHS	17	27	45	11	0	NA
5813673	KCa'b'R#7	RHS	13	33	49	0	0	VC1
4180842	KCa'b'R#8	RHS	17	21	41	5	0	DA2
478805	KCa'b'R#9	RHS	9	23	32	0	0	DP1l
16274	KCa'b'R#10	RHS	2	5	7	4	31	VL2p
175027	KCa'b'R#11	RHS	1	4	11	31	11	NA
8808581	KCa'b'R#12	RHS	0	4	7	18	0	DP1m, (VL2a), (DC1)
9117955	KCa'b'R#13	RHS	0	0	11	14	0	NA
9210	KCgR#1	RHS	0	3	17	25	0	DC1, DP1l, VA3, VA7l, VL2a
5459431	KCgR#2	RHS	1	6	7	1	0	VC3m, DL5, DA4l
8808541	KCgR#3	RHS	2	5	15	16	6	VL2a, DA2, VP1m + VP5
8808887	KCgR#4	RHS	0	4	6	15	0	DL2d, VC3m, DA4l, DM4
8822544	KCgR#5	RHS	1	3	19	8	0	VC3l, DL2v
8142	KCgR#6	RHS	1	3	6	27	0	DM4, DP1l, VA4, DM3, V
10228816	KCgR#7	RHS	0	0	14	13	0	DC1, DP1m, VC3m
10586365	KCgR#8	RHS	0	0	5	5	0	DC1, (VC1)
8361575	KCgR#9	RHS	0	0	10	4	20	(DA2), DA4l, DM5
36909	KCgR#10	RHS	1	1	7	23	0	DP1l, VA6, VC3m
10527316	KCgR#11	RHS	1	0	6	13	0	DC1, DP1m, V, VC3m
8798	KCg-s1R	RHS	4	0	4	3	0	(AV3f2#1), visual
8853436	KCg-s2L	LHS	92	18	111	6	3	AV3f2#1, (mPN), visual
8854339	tKCa'b'L#1	LHS	41	8	51	1	0	NA
8880337	tKCa'b'L#2	LHS	24	18	42	0	0	NA
8884784	tKCa'b'L#3	LHS	17	14	31	0	0	NA
8867359	KCa'b'L#1	LHS	38	19	57	3	0	VL2p
8885938	KCa'b'L#2	LHS	42	6	48	0	0	(VA7l)
8851349	KCa'b'L#3	LHS	15	23	38	0	0	(DP1m), (VM1)
8866744	KCa'b'L#4	LHS	22	14	36	0	5	NA
8869397	KCa'b'L#5	LHS	20	12	37	8	0	NA
8848927	KCa'b'L#6	LHS	17	11	28	0	0	(DP1l)
8877321	KCa'b'L#7	LHS	24	4	28	6	0	VM1
8847299	KCa'b'L#8	LHS	7	19	28	9	0	V
8870427	KCa'b'L#9	LHS	8	15	23	14	0	(VM1)
8864744	KCa'b'L#10	LHS	14	6	21	10	0	NA

8869803	KCa'b'L#11	LHS	8	8	17	12	4	NA
8865424	KCa'b'L#12	LHS	5	7	13	10	5	NA
9942769	KCgL#1	LHS	12	6	32	4	0	DC2, DM3, VA5, (VM3)
8865505	KCgL#2	LHS	3	0	6	12	0	DL2v, DM4, DM5, DP1m
8865603	KCgL#3	LHS	1	2	10	17	0	VA3, VM2, VL2p

Table S1. Table of lACA-associated Kenyon cells and their inputs, related to Figure 4. SKID: Kenyon cell skeleton ID. NAME: our assigned name. SIDE: brain hemisphere. VP3, VP2, LACA: Number of inputs from the VP3 vPN, VP2 adPN, and all lACA PNs, within the lACA. LP: Number of inputs from lACA PNs outside of the lACA. VP: Number of inputs from other VP PNs outside of the lACA. OTHER: Other inputs, including from lACA targets (Figure 5).

NEW NAME	PAN	SKID_R	SKID_L	FC MATCH	SCORE	NAMES	DRIVER	SCORE
LHAV3q1#1	5B	2315173	8873592	VGlut-F-200441?	0.643		R64A07	0.677
AV3r1#1	5C	6852116	9018826	VGlut-F-200441?	0.496		R64A07	0.690
DN1a#1	5D	4129363	8867611		NA	DN1a [2]	R43D05 [44]	0.648
PD5g1#1	5H	11210831	8883836	Gad1-F-000010?	0.469			NA
DN1a#2	5D'	3973434	9362353		NA	DN1a [2]	R43D05 [44]	0.650
LHPD5a1#1	5I	5982276	8865547	Gad1-F-000010?	0.487		R91C09?	NA
DN1-l	5E	11524453	8876600		NA		R18H11; R91C09?	0.511
LHPV4c1#1	5F	6292583	8853753	VGlut-F-800074	0.671		R64A07	0.696
LHPV4c1#2	5F'	3968249	8883804	VGlut-F-800074	0.661		R64A07	0.668
PV6r1#1	5K	4214681	8867765		NA		R80C12	0.458
VP2+ adPN	2G	1712057	8864990		NA			NA
VP2 adPN	2O	57516	9159128	Gad1-F-000090	0.615	VP2 uACT1 [3]	R21C11	0.568
LHAV3f1#1	5G	11545803	9711486	VGlut-F-200021	0.681	AV3e1#1 [4]		NA
PV6r1#2		4182361	8870514		NA		R80C12	0.501
AV3r1#2		1309241	9159383		NA		R64A07	0.663
LHPV4c1#3		1120955	8854268	VGlut-F-800074	0.688		R64A07	0.735
VP1m+VP2 lvPN1#3	3F''	57114	13931917		NA		R42H11	0.483
LHAV2d1#1	5J	11543519	2610603		NA	AV2d1#1 [4]		
LHPV4c3#1		5605174	8872558	VGlut-F-800074	0.558		R64A07	0.684
VP1m+ lvPN1	3K	192430	9588978	VGlut-F-700270?	0.579		R31H04?	0.539
PD5g1#2		4294123	8875932	Gad1-F-000010	0.542			NA
LHPV4c4#1		1091854	8880798	VGlut-F-800074	0.593		R64A07	0.683
LHAV4i1#1		3439570		Gad1-F-200137	0.649			NA
PV6r1#3		4182148	8896969		NA		R80C12	0.515
VC5+ adPN*	2N	39254	3420237	Cha-F-200265	0.579	VM6+VP1 adPN [5]		NA
PPL202#1		30571		TH-F-000011	0.610			NA
LHPV6k2#1		5062598		Gad1-F-400027	0.625		32G09?	0.631
VP1m+VP2 lvPN1#1	3F	57122	9101937		NA		R31H04	0.629
LHPV6f1#3		5824712	8877293		NA		R80C12	0.509
PV6r1#4		11128057			NA		R80C12	0.594
PD5g1#3		9951834	8880131	Gad1-F-000010	0.578			NA
SLP364#1		6292328			NA		R10G01	0.631
LHPV2a1#1		79579		Cha-F-300168	0.697		R37F05	0.615
LHPV6f1#2		8808525			NA		R80C12	0.526
PD5g1#4		8820702		Gad1-F-000010	0.638			NA
LHPV10c1#1	6E	369583			NA			NA
LHAV8a1#1		57110			NA			NA
LHPV6h1#1		9069835			NA			NA
SLP364#2		6292134			NA		R10G01	0.613
VP1m+VP2 lvPN1#2	3F'	57059	12681995		NA		R31H04	0.605
LHPV4h1#1		4044728		Cha-F-500298	NA			NA
LHPV6c1#1			2466931		NA			NA
PV7c1#1			8880705	Cha-F-000074	0.549			NA

Table S2. Table of lACA-associated non-Kenyon cells, related to Figure 5. NEW NAME: our designation, based on neuroblast lineage or lateral horn tract [67]. PAN: Figure panel where example is plotted. SKID_R: skeleton ID of RHS example. SKID_L: skeleton ID of LHS example. FC MATCH: best corresponding single-cell clone in FlyCircuit. SCORE: Average of forward and reverse NBLAST scores for Fly Circuit match. NAMES: Names used in previous publications. DRIVER: best corresponding sparse driver line. SCORE: Forward NBLAST scores for Fly Light match.

TYPE	SKID1	SKID2	SKID3	SKID4
VC5++ l2PN1*	1363077	8825246		
VC5++ l2PN2	8406430			
VP1d il2PN	192423	203504		
VP1d++ vPN	3813442	3813447	4632023	
VP1d+VP1l vPN	3813438			
VP1d+VP4 l2PN1	23005			
VP1d+VP4 l2PN2	3908507			
VP1l+VP3 ilPN	57487	57495		
VP1m l2PN	11524119			
VP1m+ lvPN	192430			
VP1m++ lvPN	57142			
VP1m+VP2 lvPN1	57059	57114	57122	
VP1m+VP2 lvPN2	4058666			
VP1m+VP5 ilPN	46105	57503		
VP2 adPN	57516			
VP2 l2PN	5672990			
VP2+ adPN	1712057			
VP2++ lv2PN	4002166			
VP2+SEZ lvPN1	57146	57166		
VP2+SEZ lvPN2	57063			
VP2+VC5 l2PN	2600341			
VP2+VP5 lvPN	57051	57134	57138	1372988
VP3 vPN	5471515			
VP3+ lPN1	11234968			
VP3+ lPN2	1356477	4869882		
VP3+VP1l ivPN	37513	45882		
VP4 vPN	1149173			
VP4+ vPN	2484510			
VP5 l2PN	4672650			
VP5+ l2PN	10078400			
VP5+SEZ adPN	14003783			
VP5+VP2 l2PN	4876532	5946848		
VP5+VP3 l2PN	4671556			

Table S3. Table of PNs pooled for downstream target analysis, related to Figure 6.
TYPE: our designated morphological type, as in Table 1. SKID: Skeleton ID.

SUPPLEMENTAL REFERENCES

1. Frechter, S., Bates, A.S., Tootoonian, S., Dolan, M.-J., Manton, J., Jamasb, A.R., Kohl, J., Bock, D., and Jefferis, G. (2019). Functional and anatomical specificity in a higher olfactory centre. Elife *8*. Available at: http://dx.doi.org/10.7554/eLife.44590.

2. Shafer, O.T., Helfrich-Förster, C., Renn, S.C.P., and Taghert, P.H. (2006). Reevaluation of Drosophila melanogaster's neuronal circadian pacemakers reveals new neuronal classes. J. Comp. Neurol. *498*, 180–193.

3. Sekiguchi, M., Inoue, K., Yang, T., Luo, D.-G., and Yoshii, T. (2020). A Catalog of GAL4 Drivers for Labeling and Manipulating Circadian Clock Neurons in. J. Biol. Rhythms *35*, 207–213.

4. Stocker, R.F., Lienhard, M.C., Borst, A., and Fischbach, K.F. (1990). Neuronal architecture of the antennal lobe in Drosophila melanogaster. Cell Tissue Res. *262*, 9–34.

5. Huoviala, P., Dolan, M.-J., Love, F.M., Frechter, S., Roberts, R.J.V., Mitrevica, Z., Schlegel, P., Bates, A.S., Aso, Y., Rodrigues, T., *et al.* (2018). Neural circuit basis of aversive odour processing in Drosophila from sensory input to descending output. bioRxiv, 1180. Available at: http://dx.doi.org/10.1101/394403.

6. Yu, H.-H., Kao, C.-F., He, Y., Ding, P., Kao, J.-C., and Lee, T. (2010). A complete developmental sequence of a Drosophila neuronal lineage as revealed by twin-spot MARCM. PLoS Biol. *8*. Available at: http://dx.doi.org/10.1371/journal.pbio.1000461.

7. Bates, A.S., Schlegel, P., Roberts, R.J.V., Drummond, N., Tamimi, I.F.M., Turnbull, R., Zhao, X., Marin, E.C., Popovici, P.D., Dhawan, S., *et al.* (2020). Complete connectomic reconstruction of olfactory projection neurons in the fly brain. bioRxiv, 9. Available at: https://www.biorxiv.org/content/10.1101/2020.01.19.911453v1.

8. Zheng, Z., Lauritzen, J.S., Perlman, E., Robinson, C.G., Nichols, M., Milkie, D., Torrens, O., Price, J., Fisher, C.B., Sharifi, N., *et al.* (2018). A Complete Electron Microscopy Volume of the Brain of Adult Drosophila melanogaster. Cell *174*, 730–743.e22.

9. Zheng, Z., Li, F., Fisher, C., Ali, I.J., Sharifi, N., Calle-Schuler, S., Hsu, J., Masoodpanah, N., Kmecova, L., Kazimiers, T., *et al.* (2020). Structured sampling of olfactory input by the fly mushroom body. bioRxiv, 8393. Available at: https://www.biorxiv.org/content/10.1101/2020.04.17.047167v2.

10. Dolan, M.-J., Frechter, S., Bates, A.S., Dan, C., Huoviala, P., Roberts, R.J., Schlegel, P., Dhawan, S., Tabano, R., Dionne, H., *et al.* (2019). Neurogenetic dissection of the lateral horn reveals major outputs, diverse behavioural functions, and interactions with the mushroom body. Elife *8*. Available at: http://dx.doi.org/10.7554/eLife.43079.
