# Supplementary material for: Connectomics Analysis Reveals First-, Second-, and Third-Order Thermosensory and Hygrosensory Neurons in the Adult Drosophila Brain
Source: Curr Biol. 2020 Aug 17;30(16):3167–3182.e4. doi: 10.1016/j.cub.2020.06.028 (PMC7443704; doi:10.1016/j.cub.2020.06.028)
Supplement: Document S2. Article plus Supplemental Information [file mmc6.pdf]

Article

Current Biology

Connectomics Analysis Reveals First-, Second-, and Third-Order Thermosensory and Hygrosensory Neurons in the Adult *Drosophila* Brain

Graphical Abstract

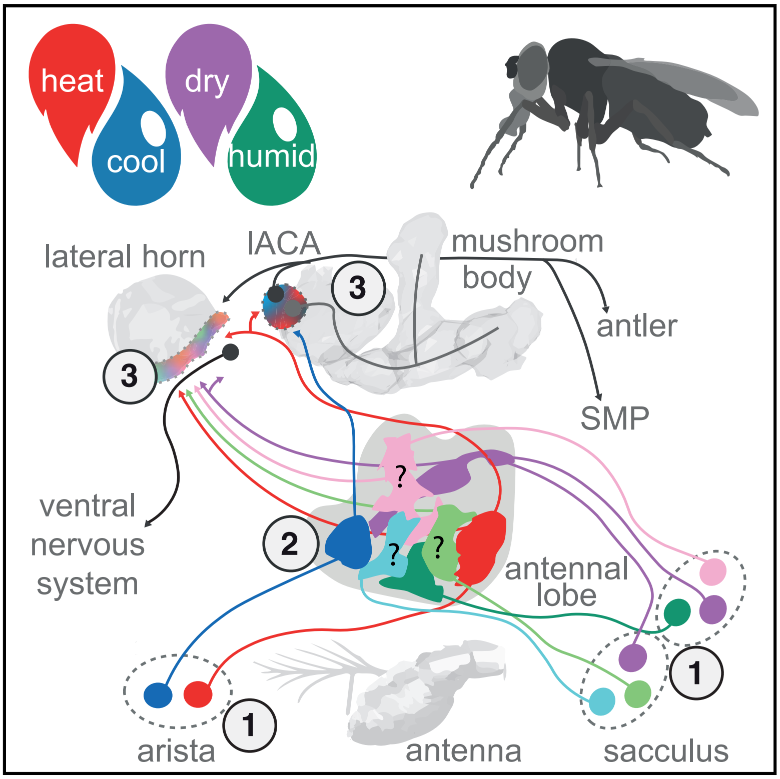

Highlights

- Two novel thermo- and/or hygrosensory glomeruli in the fly antennal lobe
- First complete set of thermosensory and hygrosensory projection neurons
- First connectome for a thermo- and hygrosensory neuropil
- Third-order thermo- and hygrosensory neurons, including link to circadian clock

Authors

Elizabeth C. Marin, Laurin Büld, Maria Theiss, ..., Davi D. Bock, Paul A. Garrity, Gregory S.X.E. Jefferis

Correspondence

jefferis@mrc-lmb.cam.ac.uk

In Brief

Marin et al. use connectomics and genetics for comprehensive identification of temperature and humidity sensory neurons in the *Drosophila* brain. They reconstruct all projections to higher brain areas and select higher-order targets, including the mushroom body lateral accessory calyx, linking thermosensation to memory and the circadian clock.

Marin et al., 2020, Current Biology *30*, 3167–3182
August 17, 2020 © 2020 MRC Laboratory of Molecular Biology.
Published by Elsevier Inc.
https://doi.org/10.1016/j.cub.2020.06.028

CellPress

Current Biology

CellPress OPEN ACCESS

Article

Connectomics Analysis Reveals First-, Second-, and Third-Order Thermosensory and Hygrosensory Neurons in the Adult *Drosophila* Brain

Elizabeth C. Marin,[1] Laurin Büld,[1] Maria Theiss,[1] Tatevik Sarkissian,[2] Ruairí J.V. Roberts,[1] Robert Turnbull,[1] Imaan F.M. Tamimi,[1] Markus W. Pleijzier,[3] Willem J. Laursen,[2] Nik Drummond,[1] Philipp Schlegel,[1,3] Alexander S. Bates,[3] Feng Li,[4] Matthias Landgraf,[1] Marta Costa,[1] Davi D. Bock,[4,5] Paul A. Garrity,[2] and Gregory S.X.E. Jefferis[1,3,6,*]

[1]Department of Zoology, University of Cambridge, Cambridge CB2 3EJ, UK
[2]Department of Biology, Brandeis University, Waltham, MA 02454, USA
[3]Division of Neurobiology, MRC Laboratory of Molecular Biology, Cambridge, Cambridgeshire CB2 0QH, UK
[4]Janelia Research Campus, Howard Hughes Medical Institute, Ashburn, VA 20147, USA
[5]Larner College of Medicine, University of Vermont, Burlington, VT 05405, USA
[6]Lead Contact
*Correspondence: jefferis@mrc-lmb.cam.ac.uk
https://doi.org/10.1016/j.cub.2020.06.028

SUMMARY

Animals exhibit innate and learned preferences for temperature and humidity—conditions critical for their survival and reproduction. Leveraging a whole-brain electron microscopy volume, we studied the adult *Drosophila melanogaster* circuitry associated with antennal thermo- and hygrosensory neurons. We have identified two new target glomeruli in the antennal lobe, in addition to the five known ones, and the ventroposterior projection neurons (VP PNs) that relay thermo- and hygrosensory information to higher brain centers, including the mushroom body and lateral horn, seats of learned and innate behavior. We present the first connectome of a thermo- and hygrosensory neuropil, the lateral accessory calyx (lACA), by reconstructing neurons downstream of heating- and cooling-responsive VP PNs. A few mushroom body-intrinsic neurons solely receive thermosensory input from the lACA, while most receive additional olfactory and thermo- and/or hygrosensory PN inputs. Furthermore, several classes of lACA-associated neurons form a local network with outputs to other brain neuropils, suggesting that the lACA serves as a hub for thermo- and hygrosensory circuitry. For example, DN1a neurons link thermosensory PNs in the lACA to the circadian clock via the accessory medulla. Finally, we survey strongly connected downstream partners of VP PNs across the protocerebrum; these include a descending neuron targeted by dry-responsive VP PNs, meaning that just two synapses might separate hygrosensory inputs from motor circuits. These data provide a comprehensive first- and second-order layer analysis of *Drosophila* thermo- and hygrosensory systems and an initial survey of third-order neurons that could directly modulate behavior.

INTRODUCTION

Temperature and humidity are interrelated environmental variables with dramatic effects on animal physiology and survival. Temperature affects all aspects of organismal function, making the maintenance of optimal body temperature and avoidance of thermal extremes critical for survival [1]. Humidity impacts hydration state, especially in insects, whose large surface-area-to-volume ratios make them vulnerable to dehydration [2]. Temperature and humidity also cue essential behavioral programs; for example, female mosquitoes use heat and humidity to locate warm-blooded hosts for blood feeding [3, 4], and temperature can entrain the circadian clock [5, 6], regulating rhythms of activity and sleep.

While fundamental for survival, the neuronal mechanisms of thermosensation and hygrosensation are not well understood. In mammals, the molecules and circuits underlying thermal nociception have been extensively investigated, but less is known regarding body temperature homeostasis [7]. In insects, hygro- and thermosensory neurons have been found on the antennae of diverse species, with individual sensilla often containing pairs of cooling- and heating-responsive neurons or triads of cold-, dry-, and humid-responsive neurons [8]. These neurons (like olfactory sensory neurons) project from the antennae to the antennal lobe (AL) in the brain, where they innervate stereotyped glomerular subcompartments [9]. Projection neurons (PNs) relay information from these glomeruli to higher brain regions including the mushroom body calyx (CA) and lateral horn (LH) of the protocerebrum [10], respectively involved in learned and innate olfactory behaviors [11].

Recent studies in *Drosophila melanogaster* provide some insight into the molecules and brain circuits underlying insect thermosensation and hygrosensation [12]. In flies, the third antennal segment contains two structures housing thermo- and

This is an open access article under the CC BY license (http://creativecommons.org/licenses/by/4.0/).

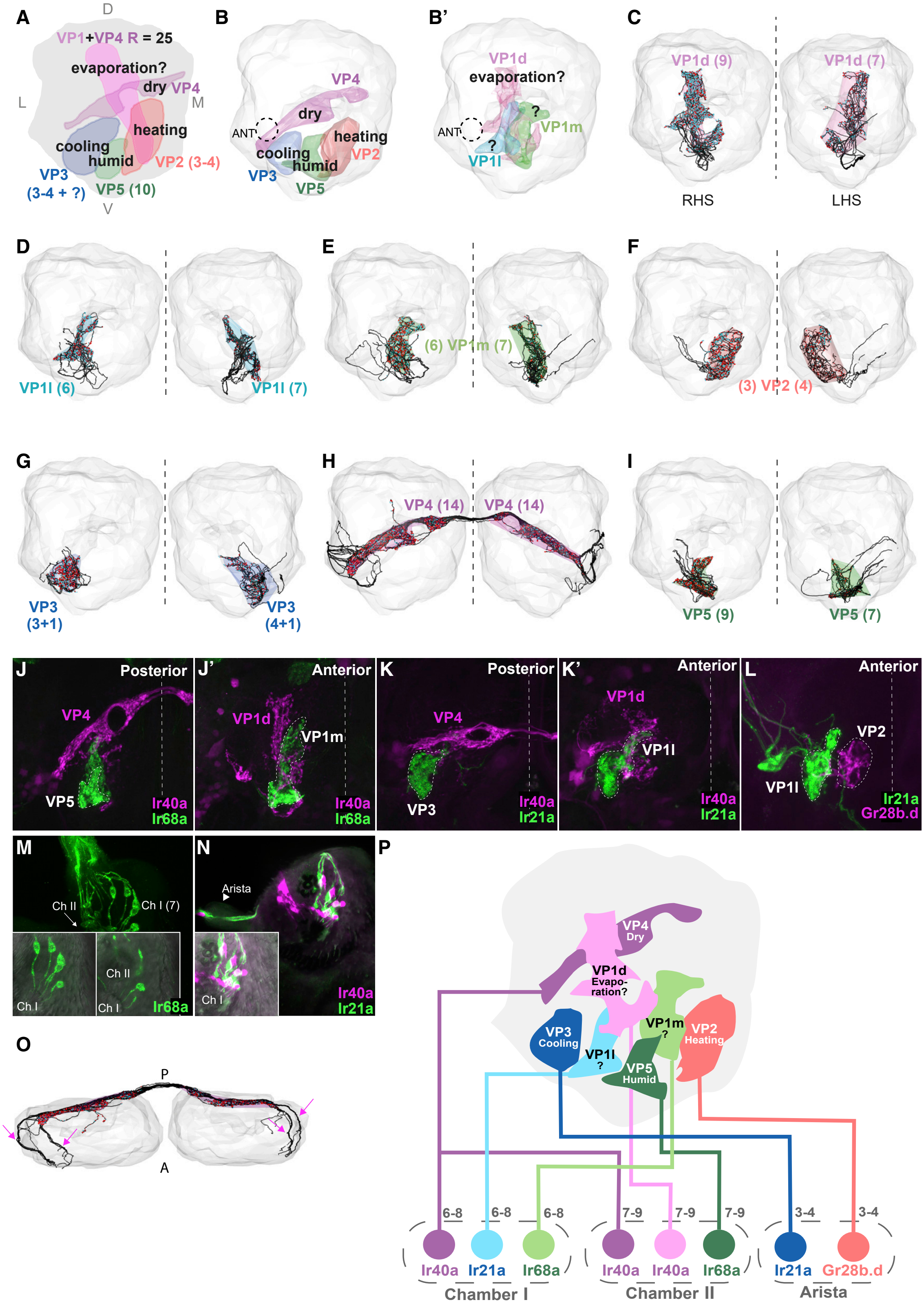

Figure 1. Sensory Neurons Define Seven Distinct Glomeruli in the Ventroposterior Antennal Lobe

(A) Frontal view illustration of the VP AL summarizing the sensory modalities, locations, and numbers of thermo- and hygrosensory neurons reported in previous studies. D: dorsal; L: lateral; M: medial; V: ventral.

(legend continued on next page)

CellPress
OPEN ACCESS

hygrosensory neurons: a feathery protrusion called the arista and a multi-chambered invagination called the sacculus [13, 14]. The arista houses three (sometimes four) pairs of phasic thermosensory neurons, one transiently activated by heating and the other by cooling [15, 16]. The sacculus contains several types of sensilla, some of which resemble thermo- and/or hygrosensory sensilla [17]. Dry-responsive neurons have been identified in chambers I and II [18, 19] and humid-responsive neurons in chamber II [20, 21].

Antennal thermo- and hygrosensory neurons innervate ventroposterior (VP) AL glomeruli. Heating-responsive aristal thermosensory neuron axons expressing the gustatory receptor isoform Gr28b.d [22] arborize in VP2 and cooling-responsive ones expressing the ionotropic receptor Ir21a [16] in VP3 [15, 23]. Saccular Ir40a-expressing, dry-air-responsive neurons arborize in VP4 [18–20, 24] and Ir68a-expressing, humid-air-responsive ones in VP5 [20, 21]. Finally, Ir40a-expressing putative thermosensors in chamber II project to VP1 [18, 19, 24] (Figure 1A); these have been reported to respond weakly to cooling [18], dry air [19], or ammonia [25] and may represent evaporative cooling.

Several PN classes relay information from VP glomeruli to higher brain centers. For example, a slow-adapting, cool-sensing ("slow-cool") VP3 PN targets the lateral accessory calyx (lACA) of the mushroom body [26, 27]. Fast-adapting thermosensory PNs target the posterior lateral protocerebrum (PLP) and posterior slope [27, 28], and a broadly tuned PN innervating several VP glomeruli targets the PLP [21]. These studies suggest some integration of thermo- and hygrosensory inputs at the level of the AL PNs and indicate that these pathways project to multiple brain regions.

Despite these advances, our current description of thermo- and hygrosensory systems in *Drosophila* remains incomplete. The sacculus contains uncharacterized sensory neurons that likely innervate unknown AL glomeruli. Uniglomerular PNs have not yet been reported for some known VP glomeruli. Finally, the only higher-order neurons identified thus far are α'/β' Kenyon cells (KCs) of the mushroom body, presumed targets of the slow-cool VP3 PN in the lACA [29].

To obtain a more comprehensive view of the thermo- and hygrosensory system, we used the full adult fly brain (FAFB) electron microscopy (EM) volume for whole neuron reconstruction and synapse annotation [30]. We identified the sensory neurons innervating five known VP glomeruli and two novel glomeruli and showed that these neurons express receptor molecules consistent with thermosensation or hygrosensation. We also reconstructed every PN projecting from VP glomeruli to higher brain centers; they are highly diverse in tract, neuropil target, and neuroblast of origin. We elucidated the "connectome" of the lACA, which not only provides input to both dedicated and integrative KCs but also serves as a hub for thermo- and hygrosensory information and a link to the circadian clock. Finally, we identified additional novel third-order neurons, including a descending neuron that relays hygrosensory information to the ventral nerve cord. Together, these data provide a comprehensive catalog of first- and second-order *Drosophila* thermo- and hygrosensory neurons and an initial survey of third-order neurons that would allow integration with other sensory modalities and modulation of behavior.

RESULTS

Sensory Neurons Define Two Novel Glomeruli in the Ventroposterior Antennal Lobe

To define the five known VP glomeruli (Figure 1A), we reconstructed all putative VP sensory neurons in both hemispheres of FAFB, completing (including synapses) those in the right-hand side (RHS) AL. We found four known VP glomeruli in the posteriormost AL (Figure 1B): VP2 (heating), VP3 (cooling), VP4 (dry), and VP5 (humid).

Three unilateral receptor neurons (RNs) with complex arbors on the RHS and four on the left-hand side (LHS) innervated VP2 (Figure 1F). Four RHS unilateral RNs (five LHS) innervated VP3 (Figure 1G), with all but one forming complex arbors (Figures S1E' and S1E'') and the last forming a simpler arbor and traveling separately (Figure S1E'''). Morphological clustering with NBLAST [31] paired the simpler VP3 RNs on each side with one another, within the larger VP3 RN group (Figure S2A), implying that they are of the same type and distinct from the complex VP3 RNs. Moreover, the simpler VP3 RN provided most of the input to the RHS slow-cool VP3 PN, whereas the others targeted distinct VP3 PNs (Figure S2B). These results are consistent with reports of three to four pairs of aristal sensory neurons that project to VP2 (heating) and VP3 (cooling) [15, 16] and an unknown number of non-aristal sensory neurons supplying the slow-cool VP3 PN [27].

We traced 28 bilateral RNs that collectively defined the VP4 glomeruli (Figure 1H). These VP4 RNs traveled in two distinct areas of each antennal nerve (Figure 1O), perhaps reflecting their origins in sacculus chambers I versus II. We also found nine unilateral RHS (seven LHS) RNs occupying VP5 (Figure 1I), consistent with previous reports [20]. Since VP4 and VP5 RNs are found

(B and B') Glomerular meshes generated from sensory neurons reconstructed in the right hemisphere of FAFB. Dashed circle labeled ANT: right antennal nerve. (B) Posterior meshes: VP2, VP3, VP4, and VP5. (B') Anterior meshes: VP1d, VP1l, and VP1m.

(C–I) ALs with glomerular meshes enclosing reconstructions of individual VP sensory neurons (in black, with presynaptic sites in red and postsynaptic sites in cyan, and number of neurons indicated in parentheses). Dashed line: the midline. (C) VP1d, (D) VP1l, (E) VP1m, (F) VP2, (G) VP3, (H) VP4, (I) VP5.

(J–L) Frontal views of receptor expression data in the AL. White dashed line: the midline. (J and J') VP1m and VP5 sensory neurons express Ir68a (green) but not Ir40a (magenta). (K and K') VP1l and VP3 sensory neurons express Ir21a (green) but not Ir40a (magenta). (L) VP2 sensory neurons express Gr28b.d (magenta), but Ir21a+ VP1l sensory neurons (green) do not.

(M) Chamber I and II sensory neurons express Ir68a (green). Inserts show bright field overlay with GFP of selected frames.

(N) Chamber I sensory neurons express Ir21a (green). Inserts show bright field overlay with GFP of selected frames. Arrowhead: Ir21a+ neurons in the arista.

(O) Dorsal view of VP4 RNs. Arrows: distinct axon bundles from each antennal nerve.

(P) A model of the organization of thermo- and hygrosensory neurons in the sacculus and arista, with the specific receptors they express and the VP AL glomeruli they innervate.

See also Figures S1 and S2.

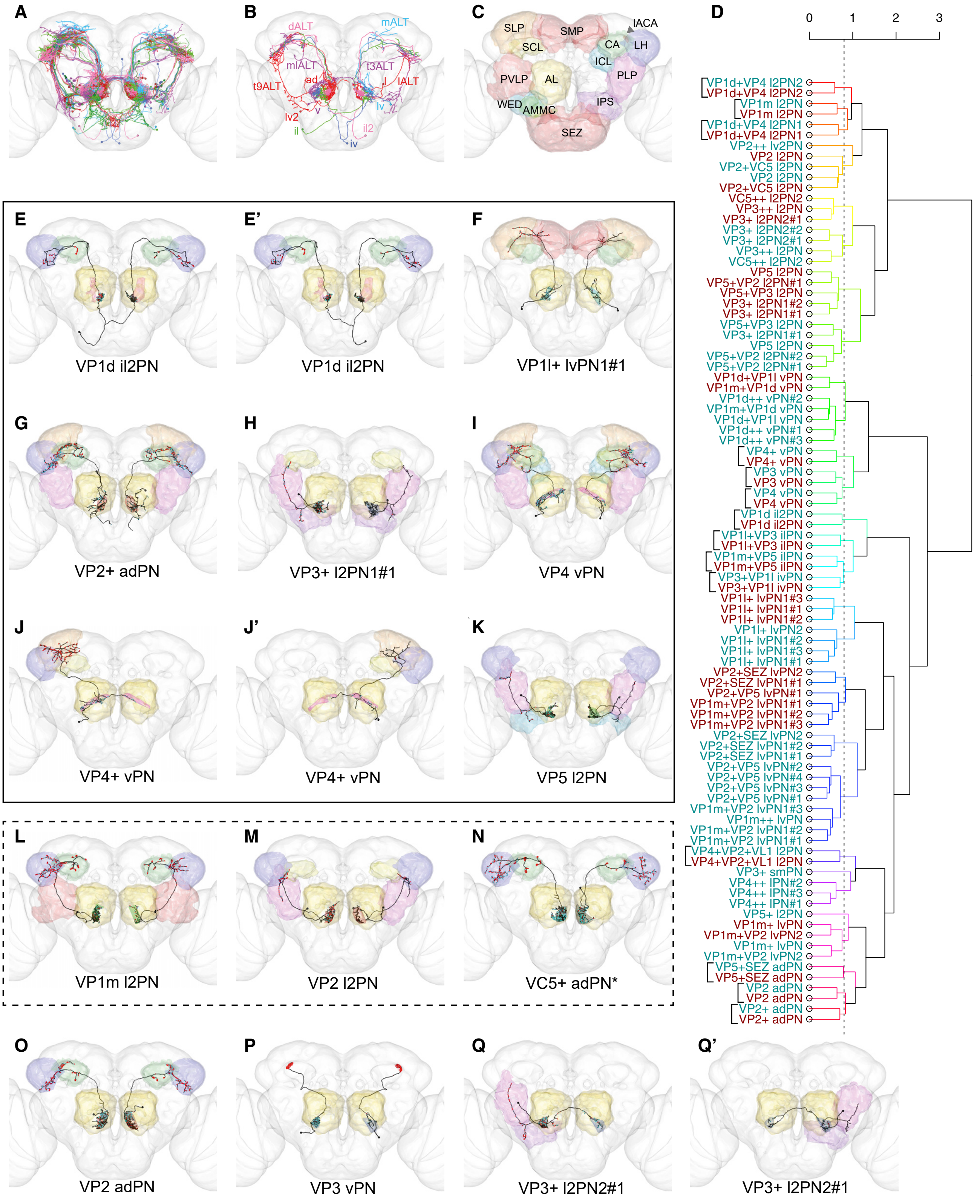

Figure 2. Projection Neurons Relay Information from Individual VP Glomeruli to Higher Brain Centers
(A) Frontal view of all VP PNs, color-coded by primary glomerulus.
(B) Summary diagram of the tracts and neuroblast lineages of VP PNs.

(legend continued on next page)

in the same triad sensilla in chamber II [20, 21, 32], this suggests that nine of 14 RHS VP4 RNs (seven of 14 LHS RNs) originated in chamber II and the remainder in chamber I.

To our surprise, unilateral RN axons in the predicted location of VP1 formed not one, but three distinct populations. These defined adjacent glomeruli (Figure 1B') that we designated according to their relative positions: VP1d, VP1l, and VP1m (Figures 1C–1E). NBLAST clustering supported six distinct classes of unilateral RNs: VP1d, VP1l, VP1m, VP2, VP3, and VP5 (Figure S2A). VP1d, VP1l, and VP1m RNs each formed synaptic connections with other neurons from the same glomerulus, consistent with our separate designations (Figure S2C).

VP1d RNs most closely resembled the Ir40a-positive "VP1" neurons in morphology and location. Light-level studies (relying on IR40a-specific antibodies and drivers) had identified a total of 25 saccular sensory neurons projecting to VP1 and/or VP4 [19, 20, 24], consistent with our VP1d and VP4 RN totals (23 RHS and 21 LHS). Moreover, we found the same numbers of VP1d and VP5 RNs in each AL (Figures 1C and 1I), suggesting that these classes might cohabit the triad sensilla in chamber II, along with the VP4 RNs [20, 21, 32].

To elucidate the likely modalities of VP1l and VP1m, we generated novel tools to label neurons expressing receptors previously associated with thermo- and hygrosensation (STAR Methods). We observed Ir68a expression in VP1m RNs as well as in VP5 (Figures 1J and 1J'). Ir21a (Figures 1K and 1K'), but not Gr28b.d (Figure 1L), was expressed in VP1l RNs, suggesting that they may be cooling responsive. These expression data, together with their characteristic neuronal and sensillar morphologies, suggest these new VP sensory neurons are thermo- or hygrosensory. Finally, Ir68a (Figure 1M) and Ir21a expression (Figure 1N) were each detected in approximately seven neurons of chamber I, and the same numbers of VP1l and VP1m RNs were reconstructed in each AL (Figures 1D and 1E). These data suggest that VP1l and VP1m RNs form a triad with the Ir40a-expressing VP4 neurons in chamber I (Figure 1N).

Our data, along with published expression data, support a model in which VP2 and VP3 are innervated by heating- and cooling-responsive sensory neurons from the arista and five additional VP glomeruli by sensory neurons from the sacculus (Figure 1P). Sensory neurons from seven to nine triad sensilla in chamber II innervate VP4 (Ir40a, dry), VP5 (Ir68a, humid), and VP1d (Ir40a, putative evaporative cooling). Sensory neurons from six to eight sensilla in chamber I innervate VP4 (Ir40a, dry), VP1l (Ir21a, putative cold/cooling), and VP1m (Ir68a, putative humid).

A Diverse Population of Antenna Lobe Projection Neurons Relays Thermo- and Hygrosensory Information to Higher Brain Centers

We next sought to identify the projection neurons (PNs) that relay activity from VP AL glomeruli to higher brain centers. By systematic tracing of the AL tracts, we identified every PN on the RHS, including 62 innervating a VP glomerulus more than any other ([33], this issue of *Current Biology*) (Figure 2A; Table 1). Ten of these are still predicted to receive majority olfactory input; the remaining 52 were designated "VP PNs" and classified into 38 morphological types, using lineage, tract, glomerulus, and axon arborization (Table 1, Type).

We also reconstructed 36 VP PNs on the LHS and matched them to RHS types. NBLAST clustering helped confirm morphological stereotypy across brain hemispheres; 14 uniquely identifiable VP PNs paired with their contralateral counterparts instead of with ipsilateral VP PNs of similar type (Figure 2D). Of our 38 types, nine had been described in the thermo- and hygrosensory light-level literature, while six had been reported but not associated with the correct glomeruli (e.g., [34, 35]) (Table 1, Previous Names), leaving 23 completely novel VP PN types.

Our reconstructed VP PNs constituted a highly diverse population. Based on their primary soma tracts, they belonged to nine distinct neuroblast lineages or hemilineages: AL anterodorsal (ad), lateral (l), ventral (v), or lateroventral (lv) [37], or the previously unidentified lateroventral 2 (lv2), inferior lateral (il), inferior lateral 2 (il2), inferior ventral (iv), or superior medial (sm) [33]. They projected to higher brain centers via any of seven AL tracts: the mALT, mlALT, and lALT; one of the small transversal tracts leaving those main tracts [36]; or an unusual route we dubbed the dALT (Figure 2B; Table 1). Like canonical excitatory olfactory PNs, many VP PNs targeted the CA and LH, but some targeted additional neuropils (Figure 2C).

Each VP glomerulus except VP1l was densely and specifically innervated by at least one "uniglomerular" PN (Figures 2E–2Q'), validating the boundaries of the VP glomeruli and our distinction between VP1d, VP1l, and VP1m. However, most VP PN types innervated multiple glomeruli (Figure 3); the most common "biglomerular" combinations were VP1l and VP3, VP1m and VP2, VP1d and VP4, and VP5 and VP2 (Table 1), suggesting that these represent stimulus combinations of particular behavioral importance. We selected two examples (VP1l+VP3 and VP1m+VP2) and completely reconstructed their dendrites in the RHS AL, finding that they sampled substantially, but not equally, from each glomerulus (Figures 3G and 3H).

Numerous PNs innervated olfactory or gustatory neuropils in addition to VP glomeruli (Figures 3K–3O'). We identified VP

(C) Summary diagram of the target brain neuropils of VP PNs, color-coded to correspond with all other figure panels. AL: antennal lobe; AMMC: antennal mechanosensory and motor center; CA: mushroom body calyx; ICL: inferior clamp; IPS: inferior posterior slope; lACA: lateral accessory calyx; LH: lateral horn; PLP: posterior lateral protocerebrum; PVLP: posterior ventral protocerebrum; SCL: superior clamp; SEZ: subesophageal zone; SLP: superior lateral protocerebrum; SMP: superior medial protocerebrum; WED: wedge.

(D) Morphological hierarchical clustering based on NBLAST scores for VP PNs in each hemisphere, cut at height 0.8 (dashed line), which groups the PNs by type. Dark cyan: RHS. Dark red: LHS. Square brackets: unique VP PNs matched across hemispheres. For each type with multiple examples, numbers (#1, etc.) reflect order of appearance in the SKID_R or SKID_L columns in Table 1.

(E–Q') Frontal views of reconstructions (black) of candidate thermo- and hygrosensory PNs that innervate one primary glomerulus (color-coded as in Figures 1B and 1B', non-VP glomeruli in gray). Presynaptic sites in red and postsynaptic sites in cyan. Neuropils color-coded as in (C). Asterisks: primarily olfactory PNs; black outline: candidate novel thermosensory PNs; dashed outline: PNs previously described but not recognized as VP or thermosensory. (E) VP1d il2PN, (E') VP1d il2PN1#1, (F) VP1l+ lvPN1#1, (G) VP2+ adPN, (H) VP3+ l2PN1#1, (I) VP4 vPN, (J) VP4+ vPN, (J') VP4+ vPN, (K) VP5 l2PN, (L) VP1m l2PN, (M) VP2 l2PN, (N) VC5+ adPN*, (O) VP2 adPN, (P) VP3 vPN, (Q) VP3+ l2PN2#1, (Q') VP3+ l2PN2#1.

CellPress OPEN ACCESS

Table 1. Candidate Thermo- and Hygrosensory Projection Neurons

Type	Tract	Panel	SKID_R	SKID_L	FC Match	Score	Novel	Previous Names	Driver	Score	NT
VP2 adPN	mALT	2O	57516	9159128	Gad1-F-000090	0.615	false	VP2 uACT1 [23]	R21C11	0.568	ACh
VP2+ adPN	mALT	2G	1712057	8864990		NA	true			NA	ACh
VP5+SEZ adPN	mALT	3L	14003783	9511430		NA	true*	poly[emb] adPN [34]		NA	ACh
VP1l+VP3 ilPN	mALT	3B, 3B'	57495	57487	Cha-F-100277	0.656	false	VP3 bilateral PN [23]	R82F02	0.521	
VP1m+VP5 ilPN	mALT	3A, 3A'	57503	46105	Gad1-F-300444	NA	false	R24G07 hPN [21]	R24G07-L [21]; R25B12	NA; 0.470	
VP1d il2PN	dALT	2E, 2E'	192423	203504	VGlut-F-000344	0.571	true		R18A08	0.531	
VP3+VP1l ivPN	mALT	3C, 3C'	45882	37513		NA	false	slow cold mALT PN [28]	VT26020 [28]; R80F03	NA; 0.636	
VP4++ lPN	mALT	S3H, S4E, S4E'	3648956, 57430, 57434		Gad1-F-700019	0.657	true		R82C09	0.679	ACh
VP4+VP2+VL1 l2PN	t3ALT	3M, 3M'	1664544	4631122		NA	false	warm-cool PN [27]	R54A03 [27]; R82C09	NA; 0.603	
VP5+ l2PN	mALT	S3E	10078400			NA	true				
VP1d+VP4 l2PN1	lALT	3D, 3D'	23005	9716626		NA	true*	biPN-2 [35]	R38A04	0.517	
VP1d+VP4 l2PN2	lALT	3E	3908507	9740174	Cha-F-500003	0.683	true*	VP3 oACT [23]	R59B04	0.641	
VP1m l2PN	lALT	2L	11524119	3078	Cha-F-500056	0.655	true*	unPN-2 [35]; lPN1 [36]		NA	
VP2 l2PN	lALT	2M	5672990	13154015		NA	true*	unPN-4 [35]	VT046265	NA	
VP2+VC5 l2PN	lALT	3N, 3N'	2600341	10515783	Cha-F-100163	0.684	true*	biPN-3 [35]	R32H03?	0.609	
VP3+ l2PN1	t10ALT	2H, S4C	11234968	13159728, 13159715	Trh-M-300005?	0.605	true		R95C02	0.638	
VP3+ l2PN2	t10ALT	2Q, 2Q', S4A	4869882, 1356477	13159741	Trh-M-300005	0.699	false	fast-cool PN [27]	R95C02 [27]	0.627	
VP3++ l2PN	t10ALT	S3D, S3D'	7017522	5946637		NA	true		R95C02?	0.506	
VP5 l2PN	t10ALT	2K	4672650	13053419	Cha-F-100123	0.680	true		R78E05	0.588	
VP5+VP2 l2PN	t10ALT	S3A, S4B	5946848, 4876532	13159719	Cha-F-100123	0.693	false	warm-PN [27]	R95C02 [27]; R78E05	NA; 0.571	
VP5+VP3 l2PN	t10ALT	S3B	4671556	9704839	Trh-M-200074	0.710	false	unPN-6 [35]; cool lALT PN [28]	R95C02 [28]	0.593	
VP1l+ lvPN1	mALT	2F, S4F, S4F'	57224, 56999, 57200	4177248, 7463926, 13492671		NA	true		R14F11?	0.564	ACh
VP1l+ lvPN2	mALT	S3I	3742499			NA	true		R14F11?	0.491	ACh
VP1m++ lvPN	mALT	S3J	57142			NA	true			NA	ACh
VP1m+VP2 lvPN1	mALT	3F, 3F', 3F''	57122, 57059, 57114	9101937, 12681995, 13931917		NA	true		R31H04	0.629	ACh

(*Continued on next page*)

Table 1. *Continued*

Type	Tract	Panel	SKID_R	SKID_L	FC Match	Score	Novel	Previous Names	Driver	Score	NT
VP2+VP5 lvPN	mALT	S3M, S4G, S4G', S4G''	57138, 57134, 57051, 1372988	11052217		NA	true		R31H04	0.601	ACh
VP2+SEZ lvPN2	mALT	S3K	57063	4495405	VGlut-F-700270?	0.579	true		R31H04?	0.539	ACh
VP2+SEZ lvPN1	mALT	3K, S4I, S4I'	57166, 57146	7423431	VGlut-F-700270	0.606	true		R31H04?	0.558	ACh
VP1m+ lvPN	t2ALT	3J	192430	9588978		NA	Ttrue		R24B03	0.657	
VP1m+VP2 lvPN2	t2ALT	3I	4058666	9590169		NA	true		R24B03	0.615	
VP2+ lv2PN	t9ALT	S3N	4002166			NA	true		R93D06?	0.564	
VP3+ smPN	mALT	S3O	57475		VGlut-F-200354?	0.628	true		R82F02	0.549	
VP1d++ vPN	mlALT	S3P, S4I, S4I'	3813447, 3813442, 4632023		VGlut-F-500199	0.682	true		R49F09	0.694	GABA
VP1d+VP1l vPN	mlALT	S3Q	3813438	5643689		NA	true		R21C11	0.660	GABA
VP1m+VP1d vPN	mlALT	S3S	3813424	13272972		NA	true		R21C11	0.675	GABA
VP4+ vPN	mlALT	2J, 2J'	2484510	12805737		NA	true		R11F08	0.600	GABA
VP3 vPN	mlALT	2P	5471515	561100	Fru-M-100050	0.523	false	AL-t5PN1 [26]; slow-cool PN [27]	R60H12 [28]	0.600	GABA
VP4 vPN	mlALT	2I	1149173	9039579	VGlut-F-300564	0.659	true		R21A01	0.616	GABA
VP1d++ lPN1*	mALT	S3F, S4D-S4D'''	37235, 37212, 182684, 57426, 57446		VGlut-F-200115	0.663	true		R47G06?	0.666	ACh
VP1l++ lPN*	mALT	S3G	57442		Cha-F-300107	0.636	true		R41H09	0.616	ACh
VP1m++ l2PN*	lALT	S3C	4195469	14005954	VGlut-F-000370?	0.541	true*	unPN-5 [35]	R47A12	0.605	
VP2++ lvPN*	mALT	S3L	57126			NA	true		R14F11?	0.494	ACh
VP1m++ smPN*	t6ALT	6D	11546775	9717951		NA	true		R82F02?	0.486	
VP1m++ vPN*	mlALT	S3R	4954519			NA	true		R49F09?	0.680	GABA
VC5+ adPN*	mALT	2N	39254	3420237	Cha-F-200265	0.579	true*	VM6+VP1 adPN [34]		NA	ACh
VC5++ l2PN1*	t3ALT	3O, 3O'	1363077, 8825246	5122761, 13950267	VGlut-F-100102	0.693	false	slow hot and cold t3ALT PN [28]	R84E08 [28]; R82C09; R13B06	NA; 0.584; 0.538	
VC5++ l2PN2	t10ALT	S3T, S3T'	8406430	5582907		NA	true*	biPN-4 [35]		NA	
VC5+ lvPN1*	mALT	S3U, S3U', S4J	57130, 57236	4724830		NA	true		R53D01?	0.538	ACh
VC5+ lvPN2*	mALT	S3V, S4K	57015, 57175			NA	true		R14F11?	0.516	ACh

Type: our designation, based on neuroblast lineage and top glomerulus innervated. Asterisk indicates PN predicted to be primarily olfactory. Tract: antennal lobe tract. Panel: corresponding panel(s) in this paper. SKID_R: RHS skeleton IDs (first listed is #1, etc.). SKID_L: LHS skeleton IDs (first listed is #1, etc.). FC Match: best corresponding single-cell clone in FlyCircuit. Score: Average of forward and reverse NBLAST scores for FC match. Previous Names: name(s) previously assigned to this type in the literature. Driver: sparse driver line(s) that label this neuron, based on previous reports and/or our NBLAST. Score: forward NBLAST score for driver. NT: likely neurotransmitter, based on neuroblast lineage and tract (please see [33] for details). See also Figures S3 and S4.

CellPress
OPEN ACCESS

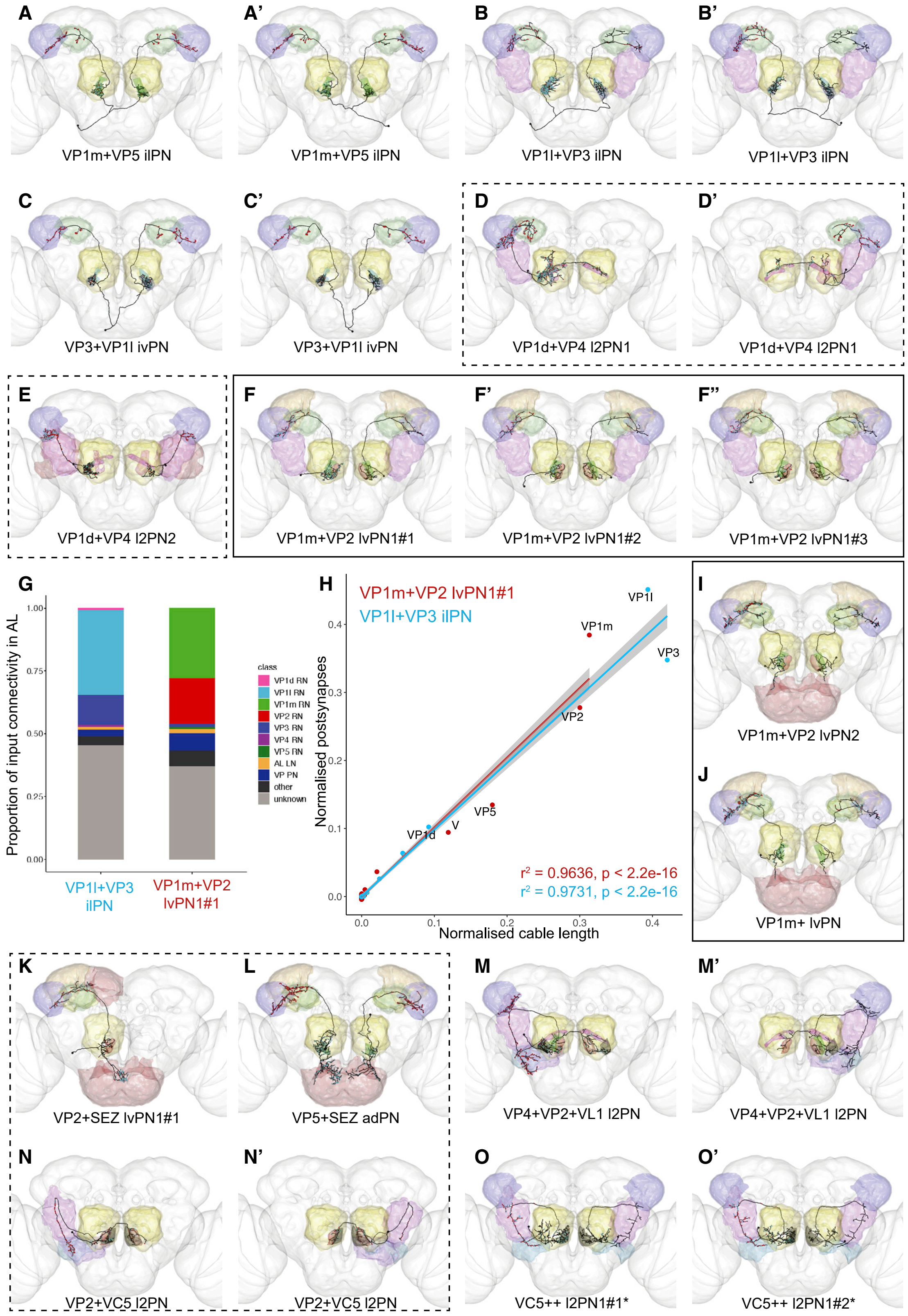

(legend on next page)

CellPress
OPEN ACCESS

PNs innervating either VP2 or VP5 [34] and the subesophageal zone (SEZ) (Figures 3K and 3L), presumably integrating temperature or humidity with taste. At least eight PNs innervated VP1d together with olfactory glomeruli (Table 1), perhaps adjusting signal strength based on rate of evaporation. Finally, we found one VC5+VP2 PN (Figures 3N and 3N') and one VC5 PN predicted to receive primarily VP input (Figures S3T and S3T'). Multiglomerular PNs innervating VC5 (Figures 3O and 3O') have previously been implicated in thermosensation [28].

In summary, we reconstructed 88 VP PNs of 38 putative morphological types, plus 5 types of PNs innervating VC5. We found anatomical (and for seven, functional) descriptions of 15 VP PN types in the *Drosophila* neurobiology literature (Table 1, Previous Names). Elucidation of our 23 novel VP PN types awaits future physiological and behavioral studies, for example, using sparse driver lines (Table 1, Driver).

VP PNs Relay Thermo- and Hygrosensory Information to the Lateral Accessory Calyx

Although the VP PNs target many different neuropils, the majority project to the CA and/or LH. Moreover, most presynaptic terminals are restricted to the lACA or anterior part of the main calyx, or the VP LH bordering the PLP (Figures 4A, 4A', and S5A). We decided to focus our tracing efforts on the lACA, which had the highest density of VP PN innervation (Figures S5B and S5C).

Only the slow-cool VP3 vPN was previously reported to innervate the lACA [26, 27]. Complete reconstruction of all RHS PN axons [33] revealed that 11 provide input to the lACA (Table S5), seven of which were conserved on the LHS: the VP3 vPN, the VP2 and VP2+ adPNs, a VP1m+VP5 ilPN, two VP1m+VP2 adPNs, and the VC5+ adPN. Axo-axonic connections between these PNs (Figures 4E, 5L, and 5M) may permit modulation of heating-responsive VP2 adPNs by the cooling-responsive VP3 vPN.

The VP3 vPN and VP2 adPN together provided the large majority (>90%) of PN presynapses to each lACA (Table S5). We annotated all of their associated postsynapses on the RHS and reconstructed a random sample (Figures 4B–4D), allowing identification of all strong downstream partners (STAR Methods; Figures S5C–S5C''). We reconstructed the same strongly connected types for the LHS VP3 vPN, suggesting stereotypy (Figures 4F–4I, 5A–5K, and S6).

Distinct classes of KCs, KCγd, KCγ, KCα'/β', KCα/βp, and KCα/β, are sequentially generated during development [38]. As expected from light-level studies [29], we recovered KCα'/β' from sampling, three of which exclusively received input from the lACA (Figure 4H). We reconstructed 13 additional KCα'/β' (Figure 4F) and 11 KCγ in the RHS lACA (Figure 4G) that also received olfactory and/or VP PN input in the CA (Figure 4E). We also reconstructed a KCγ (KCγ-s2) with unusually complex axon branching (Figure 4I) that received input from the lACA VP3 and VP2 PNs, multiglomerular PNs and thermo- and hygrosensory PNs in the CA (Figure 4E), and visual PNs in the dorsal accessory calyx (Table S1). All these lACA-associated KCs belonged to the lateral neuroblast lineage (Figure 4K). Finally, we identified a KCγ (Figure 4J) with complex axon branching and dendritic branches in the PLP and ventral accessory calyx, a visual neuropil [39, 40].

The main calycal inputs to lACA-associated KCs were not identical on both sides (Table S1). However, we found that lACA-associated KCs receive disproportionately more inputs in the main calyx from lACA PNs, especially the VC5+ adPN and VP1m+VP5 ilPNs (STAR Methods; Figure 4L). PNs activated by odors associated with decaying fruit or danger were also overrepresented; conversely, there were no inputs from pheromone-responsive PNs (Figure 4M). This suggests that, while the synaptic partners (and thus the sensory cues) of lACA-associated KCs are not completely predetermined, they may be biased during development.

Nearly two-thirds of PN inputs to the lACA were received, not by KCs, but by neurons projecting to other brain areas, primarily the LH, SMP (superior medial protocerebrum), and antlers (Figures 5A and S6; Tables S2 and S6). Many lACA target neurons received more input from lACA PNs outside of the lACA than inside (Figures 5L and 5M). Most were connected to other targets within the lACA (Figure 5L), while all were connected to at least one other target elsewhere (Figure 5M), forming a thermo- and/or hygrosensory network. In many cases, neurons of similar morphological type could not be distinguished by NBLAST (Figure S6) but exhibited distinct connectivity (e.g., Figures 5B–5E).

Notably, we reconstructed three neurons connecting the lACA and accessory medulla (Figures 5D, 5D', and 5E) that resembled DN1 neurons [41], which can be entrained to temperature [42]. Two had very similar connectivity (Figure S6B), anterior somas, and morphologies (Figures 5D and 5D') matching that of the DN1a subclass [43], and both were excellent NBLAST matches (Table S2) for DN1a driver R43D05 [44] (Figure 5D''). We called the third DN1-l (for DN1-like) (Figure 5E); it had a more posterior soma, received strong input from visual PNs (Figure S6B), and was presynaptic to most lACA network neurons and to four out of seven lACA PNs (Figure 5M). This suggests a distinct role such as modulating circadian rhythms of temperature preference [45].

Figure 3. Projection Neurons Relay Information from Multiple VP Glomeruli and Other Modalities to Higher Brain Centers

(A–F''') Frontal views of reconstructions (black) of candidate thermo- and hygrosensory PNs that innervate more than one VP glomerulus. (A) VP1m+VP5 ilPN, (A') VP1m+VP5 ilPN, (B) VP1l+VP3 ilPN, (B') VP1l+VP3 ilPN, (C) VP3+VP1l ivPN, (C') VP3+VP1l ivPN, (D) VP1d+VP4 l2PN1, (D') VP1d+VP4 l2PN1, (E) VP1d+VP4 l2PN2, (F) VP1m+VP2 lvPN1#1, (F') VP1m+VP2 lvPN1#2, (F'') VP1m+VP2 lvPN1#3.

(G and H) Proportion of inputs of each type (G) and best fit line to linear regression model plotted in R with the lm() function showing correlation of cable length in each glomerulus with RN input for two RHS multiglomerular VP PNs, VP1m+VP2 lvPN1#1 and VP1l+VP3 ilPN (H).

(I–L) Frontal views of reconstructions (black) of candidate thermo- and hygrosensory PNs that innervate the subesophageal ganglion (red) as well as VP glomeruli. (I) VP1m+VP2 lvPN2, (J) VP1m+ lvPN, (K) VP2+SEZ lvPN1#1, (L) VP5+SEZ adPN.

(M–O') Frontal views of reconstructions (black) of candidate thermo- and hygrosensory PNs that innervate non-VP glomeruli. Presynaptic sites in red and postsynaptic sites in cyan. Brain neuropils color-coded as in Figure 2C. VP glomeruli color-coded as in Figures 1B and 1B'; non-VP glomeruli in gray. Black outline: candidate novel thermosensory PNs; dashed outline: PNs previously described but not recognized as VP or thermosensory, or associated with the wrong glomeruli. (M) VP4+VP2+VL1 l2PN, (M') VP4+VP2+VL1 l2PN, (N) VP2+VC5 l2PN, (N') VP2+VC5 l2PN, (O) VC5++ l2PN1#1●, (O') VC5++ l2PN1#2*.

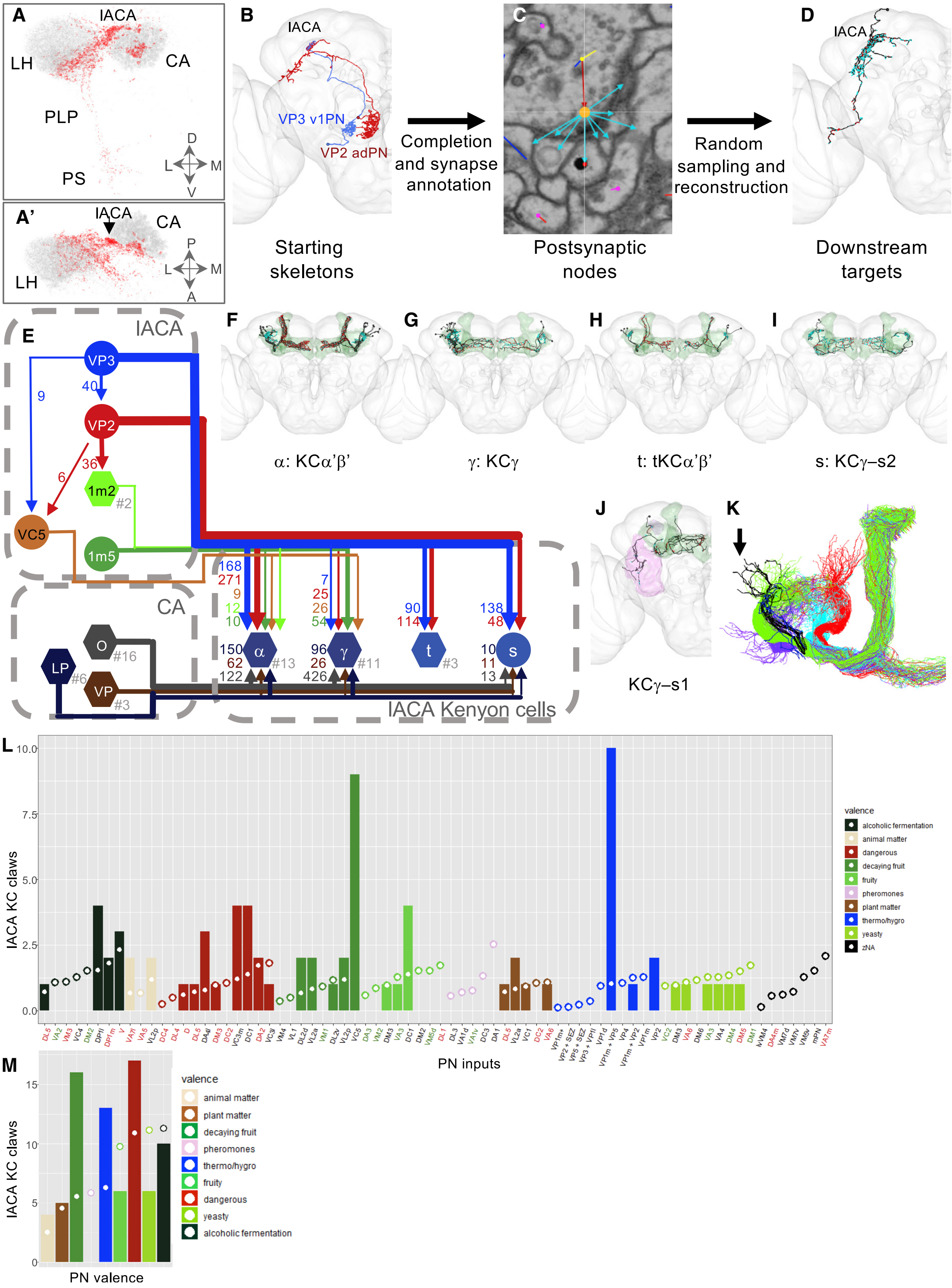

Figure 4. Both Dedicated and Mixed Modality Kenyon Cells Receive Thermosensory Input from the Lateral Accessory Calyx

(A and A') Frontal (A) and dorsal (A') views of VP PN presynapses (red) versus olfactory PN presynapses (gray). CA: mushroom body main calyx; LH: lateral horn; PLP: posterior lateral protocerebrum; PS: posterior slope.

(legend continued on next page)

In summary, we found that seven PNs (representing VP3, VP2, VC5, VP1m+VP2, and VP1m+VP5) consistently targeted the lACA, providing inputs to dedicated thermosensory and integrative KCs (Figure 4), a partially overlapping subset of PNs, and at least 19 other neuron types (Figure 5; Table S2). Most lACA target neurons were not connected to Kenyon cells, but rather to each other, forming a local thermo- and/or hygrosensory network with outputs to circadian circuits and to brain regions implicated in innate and learned olfactory responses.

VP PNs Project to Diverse Targets, Including at Least One Descending Neuron

We noticed that many targets receiving strong VP3 and VP2 input in the lACA also received VP PN input in other neuropils. We considered the entire population of VP PNs and identified the 18 most strongly connected reconstructed partners, with >150 inputs from VP PNs. However, since tracing in this dataset has been focused on the LH and CA, many VP PNs projecting to other neuropils shared no connections with those targets and were therefore excluded from further analysis.

Of the strong targets (Figure 6A), two were themselves VP PNs with arbors in the VP LH (Figures 3J and 3K), and ten received significant input in the lACA (Figure 5). We selected five of the remaining six neurons to showcase their diversity: a local LH interneuron, two multiglomerular projection neurons, an LH-SLP (superior lateral protocerebrum) and SEZ connector, and a descending neuron (Figures 5B–5F). All received substantial VP PN input inside or at the border of the LH, except for the VM1++ l2PN, which received it via dendrodendritic synapses in the AL [33].

We tabulated all VP PN input (Table S3) for each of these neurons (Figure 6G), also noting the modality or category (Figure 6H). Strikingly, the VP3 vPN alone supplied ~80% of the inputs to AV3r1#1, indicating that this target neuron is specialized for cooling. In contrast, LHAV3q1 was broadly tuned to thermo- and hygrosensory input from multiple VP glomeruli, demonstrating distinct innervation patterns of LH neurons of similar morphology. Along the same lines, the two putative DN1a neurons received very similar inputs, while DN1-l integrated inputs from the VP3 vPN and VP1m+VP2 lvPNs with substantial visual input.

The KCγ-s2 neuron received 70% of its inputs from VP PNs, primarily the VP3 vPN, but also ~20% from lACA target neurons. This suggests that it has a special role in the mushroom body for more complex integration of thermosensory information in temperature memory or preference.

VP1d (possibly evaporation) and VP1m (possibly humidity) PNs were strong upstream partners of LH neurons with large proportions of their postsynaptic budgets dedicated to olfactory input. The LH is therefore a site of multimodal integration where innate valence responses might change with perceived concentration [46, 47], in turn dependent on environmental humidity conditions and/or volatility of the odorant.

Finally, the large proportion of dry-responsive VP4 PN input to the DNp44 descending neuron might represent the shortest known *Drosophila* brain circuit from sensory periphery to descending motor control (two synapses). It could mediate a "reflex"-like escape response to dryness, as *Drosophila*, like all invertebrates, are particularly sensitive to desiccation through cuticular evaporation.

DISCUSSION

Neural circuits for temperature and humidity are vital for survival and reproduction, but they are much less well-characterized than the olfactory system, with which they share many anatomical features. Thermo- and hygrosensory RNs in *Drosophila* are housed in the antennae, innervate dedicated glomeruli in the AL, and target stereotyped projection neurons that relay information to higher brain centers to mediate innate and learned behaviors [48]. By reconstructing individual neurons and their synapses in a whole-brain EM volume, we have identified novel primary, secondary, and tertiary neurons of the thermo- and hygrosensory systems.

We reconstructed sensory neurons in the five known VP glomeruli and two novel ones. Surprisingly, the average number per VP glomerulus was only approximately one-quarter that of olfactory glomeruli [49]. Our VP1d most likely corresponds to the glomerulus originally designated VP1, which may represent evaporative cooling. We suggest, based on Ir receptor expression, that VP1l might represent cooling, while VP1m might represent humidity, with confirmation awaiting future physiological and behavioral experiments.

We reconstructed 89 VP PNs (comprising 38 morphological types, 23 completely novel) connecting VP glomeruli to higher brain centers; further studies will be necessary to define their valences and functions. Many stereotyped VP PNs received substantial input from more than one glomerulus, typically additional VP glomeruli but also VC5; we report 13 VC5 PNs (Table 1) and

(B–D) Workflow for randomized downstream sampling from key VP PNs in the lACA. (B) Frontal view of reconstructed VP PNs used for sampling from the lACA in the right hemisphere of FAFB. (C) Annotation of presynapses and associated postsynapses from these two VP PNs in the lACA. Orange: presynapse; cyan arrow: edge to associated postsynapse. (D) Frontal view of an example target neuron reconstructed during the sampling process.

(E) Partial circuit diagram illustrating connectivity between lACA-associated KCs receiving at least five inputs in the lACA and their VP and olfactory inputs. Circular nodes represent individual neurons; hexagonal nodes represent pooled neurons, with the number pooled after the hashtag. Synapse numbers correlate with line thicknesses. lACA: PNs targeting KCs in the lACA; VP3: VP3 vPN; VP2: VP2 adPN; 1m2: VP1m+VP2 lvPN1#1-2; VC5: VC5+ adPN; 1m5: VP1m+VP5 ilPN; CA: olfactory PNs (O), lACA PNs (LP), and other VP PNs (VP) targeting KC dendrites in the main calyx.

(F–J) Frontal views of lACA-associated KCs. Green: whole mushroom body; purple: dACA (J only). (F) α: KCα'β', (G) γ: KCγ, (H) t: tKCα'β', (I) s: KCγ-s2, (J) KCγ-s1.

(K) lACA-associated KCs (black) derive from one (green) of four mushroom body neuroblast lineages (green/cyan/red/violet).

(L) Expected (circles) versus observed (bars) numbers of lACA KC dendritic claws receiving input from olfactory and VP PN partners in the CA (color-coded by valence, key on right). PN inputs labeled as aversive (red), attractive (green), or unknown (black).

(M) Summary of expected (circles) versus observed (bars) numbers of lACA KC dendritic claws receiving input of various valences (key on right) from olfactory and VP PN partners in the CA.

See also Figure S5 and Table S1.

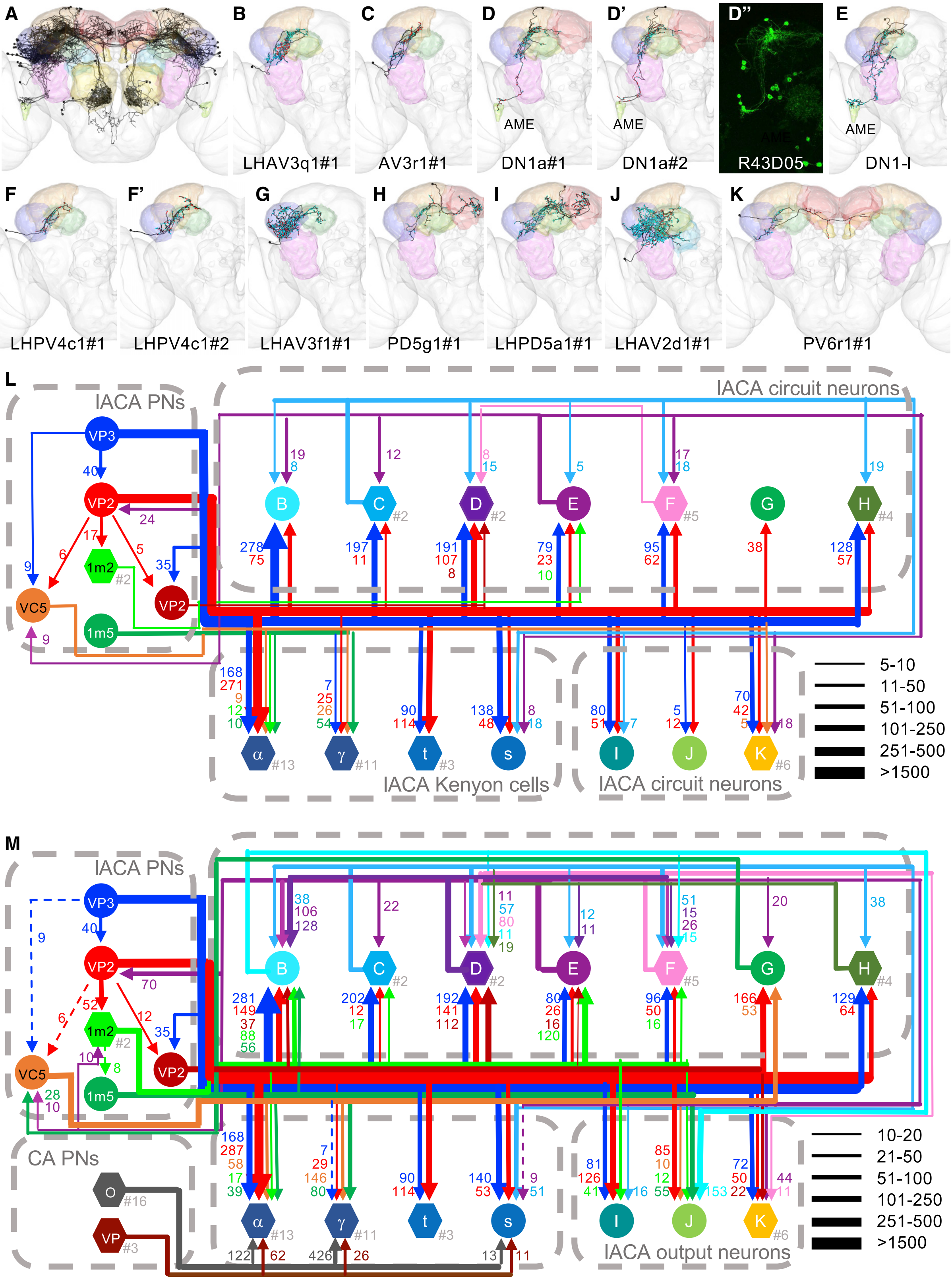

(legend on next page)

CellPress
OPEN ACCESS

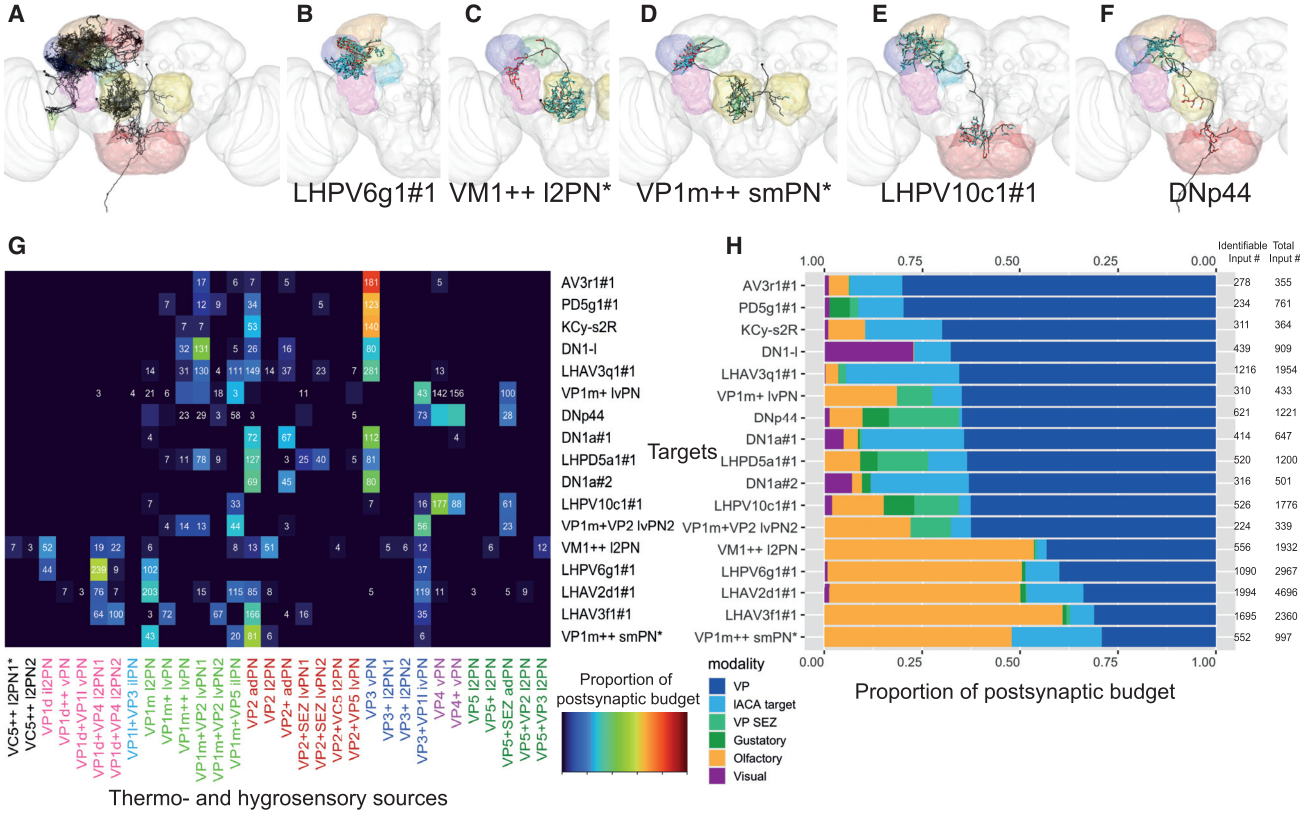

Figure 6. VP PNs Relay Information to Diverse Targets, Including a Descending Neuron

(A–F) Frontal views of selected strong downstream targets of RHS and bilaterally symmetric VP PNs, with brain neuropils color coded as in Figure 2C. Reconstructed neurons in black, with presynaptic sites in red and postsynaptic sites in cyan. (A) Seventeen strong downstream targets of VP PNs. (B) Local LH neuron LHPV6g1#1. (C) VM1++ l2PN. (D) VP1m++ smPN*. (E) LH and SEZ interneuron LHPV10c1#1. (F) Descending neuron DNp44.

(G) Heatmap showing contributions by VP PN classes to 17 downstream targets, normalized with total VP PN input = 1. Cell numbers denote raw connectivity. Heatmap is thresholded >2 synapses. Source labels are color coded by top glomerulus. See also Table S3.

(H) Summary stacked bar graph showing proportion of inputs from VP, VP + SEZ, gustatory, olfactory, and visual PNs, and lACA target neurons onto each of the 17 targets, normalized with total identified inputs = 1. Total inputs and total identified inputs are shown on the right. For both (G) and (H), only the dendrites of VM1++ l2PN and KCγ-s2R and the axon of VP1m++ smPN were considered. See also Table S3.

speculate that VC5 represents temperature and/or humidity directly or olfactory inputs especially sensitive to temperature and/or humidity. Biglomerular PNs generally innervated two glomeruli of the same predicted modality (e.g., VP1l+VP3) or paired humidity with temperature (e.g., VP5+VP2). Integration of antagonistic inputs (e.g., heating and cooling or dry and humid) was more characteristic of third-order neurons.

The lACA had previously been identified as a mushroom body accessory calyx targeted by the slow-cool VP3 vPN [27]. We found that several other VP PNs target this structure, most notably the VP2 adPN, as does the VC5+ adPN, again hinting that this glomerulus might be thermo- or hygrosensory. Unexpectedly, both Kenyon cells and other target neurons generally received more input from lACA-innervating PNs outside of the lACA neuropil than inside it. We therefore speculate that one important role of the lACA is to permit modulation of thermo- and hygrosensory PNs and targets by the slow-cool VP3 vPN en route to other neuropils.

Figure 5. The Lateral Accessory Calyx Is a Thermo- and Hygrosensory Hub with Outputs to Multiple Neuropils

(A–K, except D'') Frontal views of reconstructions (black) of non-KC lACA target neurons. Brain neuropils have been color-coded to correspond with all other figure panels. (A) All identified targets with at least five connections to lACA PNs within the lACA. (B–K) Strong downstream targets of lACA PNs. Presynaptic sites in red and postsynaptic sites in cyan. (B) LHAV3q1#1, (C) AV3r1#1, (D) DN1a#1, (D') DN1a#2, (E) DN1-l, (F) LHPV4c1#1, (F') LHPV4c1#2, (G) LHAV3f1#1, (H) PD5g1#1, (I) LHPD5a1#1, (J) LHAV2d1#1, (K) PV6r1#1. AME: accessory medulla.

(D'') Gal4 driver line labeling DN1a neurons (source: Fly Light).

(L–M) Circuit diagrams depicting (L) within-lACA and (M) total connectivity between lACA PNs and their strong downstream targets. Letters correspond to those of panels in this figure (except for elements carried over from Figure 3 and dark red VP2: VP2+ adPN). Circular nodes represent individual neurons; hexagonal nodes represent pooled neurons, with the number pooled after the hashtag. Synapse numbers correlate with line thicknesses, with dashed arrows in (M) representing inputs from (L) of <10 connections. CA PNs: olfactory PNs (O) and VP PNs (VP) targeting KC dendrites in the main calyx. lACA output neurons: neurons receiving input from lACA PNs that have <10 outputs to other lACA circuit neurons.

See also Figure S6 and Table S2.

By characterizing the connectome of the RHS lACA, we identified three α'/β' KCs specialized for thermosensation and 24 others that integrated thermosensory with olfactory and/or hygrosensory information. The two unique lACA-associated KCγ neurons exhibited highly branched axons that wrapped around the γ lobe instead of projecting to its dorsal tip like those of KCγds [36]. Future analysis of downstream partners should reveal whether these connect to distinct subsets of dopaminergic neurons (DANs) and/or mushroom body output neurons (MBONs) to influence memory formation or retrieval.

We also identified 19 novel classes of lACA-associated neurons, most of which connect not to KCs, but rather to each other, to lACA PNs, and/or to as-yet unidentified neurons in other neuropils. Three of the lACA network's main output neuropils are the LH, SMP, and antlers, all previously associated with olfactory responses [50]; in particular, the SMP features both DAN inputs and MBON outputs [39]. Indeed, two neurons had previously been identified as downstream targets of DA2 PNs, an aversive olfactory channel [51]. However, the specific downstream partners of these lACA output neurons have yet to be identified.

We reconstructed two putative DN1a neurons [43] and a third neuron that was morphologically similar but upstream of them (Figure 5). DN1 neurons have been reported to entrain the circadian clock to temperature in the absence of light signals, firing in the 20°C–29°C temperature range [52]. We observed DN1 innervation by both cooling- and heating-responsive VP PNs (Figures 5L and 6G), which could reflect sensitivity to temperature fluctuations at higher temperature ranges for adjusting temperature preferences more dynamically.

We found that while distinct subsets of thermo- and hygrosensory inputs were spatially segregated in the AL, they were integrated with each other and with olfactory inputs deeper in the pathway. It seems reasonable to hypothesize that the rate of encountering odor molecules changes with varying temperature and humidity, requiring some knowledge of context for accurate odor perception. It is also plausible that antennal thermo- and hygrosensory circuits are more ancient, with neurons for detecting, remembering, and responding to specific volatile chemicals evolving later.

While this study has significantly improved our picture of thermo- and hygrosensory circuitry, many gaps remain at the level of third-order target neurons in higher brain areas. Given the time-consuming labor of manual reconstruction, our efforts were focused on the lACA. Taking full advantage of whole EM volumes to elucidate the organization of brainwide circuits, and making comparisons between animals to assess natural and experimental variation, will require rapid and accurate automated segmentation of neuronal profiles and their synapses [53]. This study should provide a lasting platform for this next stage of exploration and the functional studies that will accompany them.

STAR★METHODS

Detailed methods are provided in the online version of this paper and include the following:

- KEY RESOURCES TABLE
- RESOURCE AVAILABILITY
  - ○ Lead Contact
  - ○ Materials Availability
  - ○ Data and Code Availability
- EXPERIMENTAL MODEL AND SUBJECT DETAILS
- METHOD DETAILS
  - ○ *Drosophila* Strains and Driver Line Generation
  - ○ Immunohistochemistry
  - ○ IHC Image Acquisition
  - ○ Sparse EM Reconstruction
  - ○ Analysis and Representation of Traced Skeletons
  - ○ Neuron Clustering via NBLAST
  - ○ Light versus EM Comparisons of Neuron Morphology
  - ○ Neuronal Nomenclature
- QUANTIFICATION AND STATISTICAL ANALYSIS
  - ○ Analysis of Biglomerular PN Inputs
  - ○ Analysis of lACA Circuit Connectivity
  - ○ Analysis of lACA-associated Kenyon Cell Inputs

SUPPLEMENTAL INFORMATION

Supplemental Information can be found online at https://doi.org/10.1016/j.cub.2020.06.028.

ACKNOWLEDGMENTS

We thank Zachary Knecht for preliminary experiments and assistance with glomerular identification and Shigehiro Namiki for assistance with identification of DNp44. We thank all contributing CATMAID tracers from the FAFB community, especially Ilenia Salaris, Jawaid Ali, Najla Masoodpanah, and Laia Serratosa Capdevila. We thank Tom Kazimiers for maintaining CATMAID and the instances used for this study, Peter Li for access to his FAFB automatic segmentation results, and Eric Perlman for making these available in CATMAID. We thank all members of the Jefferis and *Drosophila* Connectomics groups who shared code adapted for use in this study, particularly Kimberly Meechan, Fiona Love, Amelia Edmondson-Stait, and Istvan Taisz. We also thank Laia Serratosa Capdevila for helpful comments on the manuscript. This work was supported by a Wellcome Trust Collaborative Award (203261/Z/16/Z to G.S.X.E.J., M.L., and D.D.B.), a National Institute of General Medical Sciences training grant (T32 GM07122 to T.S.), grants from the National Institute of Allergy and Infectious Diseases (R01 AI122802 and R21 AI40018) and National Science Foundation (IOS 1557781) to P.A.G., MRC LMB graduate studentship to M.W.P., a Boehringer Ingelheim Fonds PhD fellowship and a Herchel Smith studentship to A.S.B., a Cambridge Neuroscience-PSL collaborative grant supported by the Embassy of France in London to G.S.X.E.J., and core support from the MRC (MC-U105188491) to G.S.X.E.J.

AUTHOR CONTRIBUTIONS

E.C.M., P.A.G., M.C., and G.S.X.E.J. conceived the project and designed experiments. T.S. performed and analyzed immunohistochemistry experiments. E.C.M., L.B., M.T., M.W.P., R.J.V.R., R.T., I.F.M.T., N.D., P.S., A.S.B., M.C., F.L., D.D.B., and G.S.X.E.J. carried out EM tracing and data analysis. W.J.L., T.S., P.A.G., P.S., A.S.B., M.C., and G.S.X.E.J. built novel molecular or computational tools. M.L., D.D.B., and G.S.X.E.J. obtained the funding. The manuscript was written by E.C.M. and P.A.G. and revised by G.S.X.E.J. and M.C. with input from all authors.

DECLARATION OF INTERESTS

The authors declare no competing interests.

Received: January 21, 2020
Revised: May 8, 2020
Accepted: June 8, 2020
Published: July 2, 2020

CellPress
OPEN ACCESS

REFERENCES

1. Huey, R.B., and Kingsolver, J.G. (2011). Variation in universal temperature dependence of biological rates. Proc. Natl. Acad. Sci. USA *108*, 10377–10378.
2. Chown, S.L., Sørensen, J.G., and Terblanche, J.S. (2011). Water loss in insects: an environmental change perspective. J. Insect Physiol. *57*, 1070–1084.
3. Brown, A.W.A. (1966). The attraction of mosquitoes to hosts. JAMA *196*, 249–252.
4. Howlett, F.M. (1910). The Influence of Temperature upon the Biting of Mosquitoes. Parasitology *3*, 479–484.
5. Liu, Y., Merrow, M., Loros, J.J., and Dunlap, J.C. (1998). How temperature changes reset a circadian oscillator. Science *281*, 825–829.
6. Buhr, E.D., Yoo, S.-H., and Takahashi, J.S. (2010). Temperature as a universal resetting cue for mammalian circadian oscillators. Science *330*, 379–385.
7. Vriens, J., Nilius, B., and Voets, T. (2014). Peripheral thermosensation in mammals. Nat. Rev. Neurosci. *15*, 573–589.
8. Altner, H., and Loftus, R. (1985). Ultrastructure and Function of Insect Thermo- And Hygroreceptors. Annual Review of Entomology *30*, 273–295.
9. Nishikawa, M., Yokohari, F., and Ishibashi, T. (1995). Central projections of the antennal cold receptor neurons and hygroreceptor neurons of the cockroach Periplaneta americana. J. Comp. Neurol. *361*, 165–176.
10. Nishikawa, M., Yokohari, F., and Ishibashi, T. (1991). Deutocerebral interneurons responding to thermal stimulation on the antennae of the cockroach,Periplaneta americana L. Naturwissenschaften *78*, 563–565.
11. Masse, N.Y., Turner, G.C., and Jefferis, G.S.X.E. (2009). Olfactory information processing in Drosophila. Curr. Biol. *19*, R700–R713.
12. Barbagallo, B., and Garrity, P.A. (2015). Temperature sensation in Drosophila. Curr. Opin. Neurobiol. *34*, 8–13.
13. Altner, H., Schaller-Selzer, L., Stetter, H., and Wohlrab, I. (1983). Poreless sensilla with inflexible sockets. A comparative study of a fundamental type of insect sensilla probably comprising thermo- and hygroreceptors. Cell Tissue Res. *234*, 279–307.
14. Sayeed, O., and Benzer, S. (1996). Behavioral genetics of thermosensation and hygrosensation in Drosophila. Proc. Natl. Acad. Sci. USA *93*, 6079–6084.
15. Gallio, M., Ofstad, T.A., Macpherson, L.J., Wang, J.W., and Zuker, C.S. (2011). The coding of temperature in the Drosophila brain. Cell *144*, 614–624.
16. Budelli, G., Ni, L., Berciu, C., van Giesen, L., Knecht, Z.A., Chang, E.C., Kaminski, B., Silbering, A.F., Samuel, A., Klein, M., et al. (2019). Ionotropic Receptors Specify the Morphogenesis of Phasic Sensors Controlling Rapid Thermal Preference in Drosophila. Neuron *101*, 738–747.e3.
17. Ferris, C.F. (1965). External morphology of the adult. In Biology of Drosophila, M. Demerec, ed. (New York: Hafner), pp. 368–419.
18. Enjin, A., Zaharieva, E.E., Frank, D.D., Mansourian, S., Suh, G.S.B., Gallio, M., and Stensmyr, M.C. (2016). Humidity Sensing in Drosophila. Curr. Biol. *26*, 1352–1358.
19. Knecht, Z.A., Silbering, A.F., Ni, L., Klein, M., Budelli, G., Bell, R., Abuin, L., Ferrer, A.J., Samuel, A.D., Benton, R., and Garrity, P.A. (2016). Distinct combinations of variant ionotropic glutamate receptors mediate thermosensation and hygrosensation in *Drosophila*. eLife *5*, e17879.
20. Knecht, Z.A., Silbering, A.F., Cruz, J., Yang, L., Croset, V., Benton, R., and Garrity, P.A. (2017). Ionotropic Receptor-dependent moist and dry cells control hygrosensation in *Drosophila*. eLife *6*, e26654.
21. Frank, D.D., Enjin, A., Jouandet, G.C., Zaharieva, E.E., Para, A., Stensmyr, M.C., and Gallio, M. (2017). Early Integration of Temperature and Humidity Stimuli in the Drosophila Brain. Curr. Biol. *27*, 2381–2388.e4.
22. Ni, L., Bronk, P., Chang, E.C., Lowell, A.M., Flam, J.O., Panzano, V.C., Theobald, D.L., Griffith, L.C., and Garrity, P.A. (2013). A gustatory receptor paralogue controls rapid warmth avoidance in Drosophila. Nature *500*, 580–584.
23. Stocker, R.F., Lienhard, M.C., Borst, A., and Fischbach, K.F. (1990). Neuronal architecture of the antennal lobe in Drosophila melanogaster. Cell Tissue Res. *262*, 9–34.
24. Silbering, A.F., Rytz, R., Grosjean, Y., Abuin, L., Ramdya, P., Jefferis, G.S.X.E., and Benton, R. (2011). Complementary function and integrated wiring of the evolutionarily distinct Drosophila olfactory subsystems. J. Neurosci. *31*, 13357–13375.
25. Silbering, A.F., Bell, R., Münch, D., Cruchet, S., Gomez-Diaz, C., Laudes, T., Galizia, C.G., and Benton, R. (2016). Ir40a neurons are not DEET detectors. Nature *534*, E5–E7.
26. Jenett, A., Rubin, G.M., Ngo, T.-T.B., Shepherd, D., Murphy, C., Dionne, H., Pfeiffer, B.D., Cavallaro, A., Hall, D., Jeter, J., et al. (2012). A GAL4-driver line resource for Drosophila neurobiology. Cell Rep. *2*, 991–1001.
27. Liu, W.W., Mazor, O., and Wilson, R.I. (2015). Thermosensory processing in the Drosophila brain. Nature *519*, 353–357.
28. Frank, D.D., Jouandet, G.C., Kearney, P.J., Macpherson, L.J., and Gallio, M. (2015). Temperature representation in the Drosophila brain. Nature *519*, 358–361.
29. Yagi, R., Mabuchi, Y., Mizunami, M., and Tanaka, N.K. (2016). Convergence of multimodal sensory pathways to the mushroom body calyx in Drosophila melanogaster. Sci. Rep. *6*, 29481.
30. Zheng, Z., Lauritzen, J.S., Perlman, E., Robinson, C.G., Nichols, M., Milkie, D., Torrens, O., Price, J., Fisher, C.B., Sharifi, N., et al. (2018). A Complete Electron Microscopy Volume of the Brain of Adult Drosophila melanogaster. Cell *174*, 730–743.e22.
31. Costa, M., Manton, J.D., Ostrovsky, A.D., Prohaska, S., and Jefferis, G.S.X.E. (2016). NBLAST: Rapid, Sensitive Comparison of Neuronal Structure and Construction of Neuron Family Databases. Neuron *91*, 293–311.
32. Shanbhag, S.R., Singh, K., and Singh, R.N. (1995). Fine structure and primary sensory projections of sensilla located in the sacculus of the antenna of Drosophila melanogaster. Cell Tissue Res. *282*, 237–249.
33. Bates, A.S., Schlegel, P.S., Roberts, R.J.V., Drummond, N., Tamimi, I.F.M., Turnbull, R., et al. (2020). Complete connectomic reconstruction of olfactory projection neurons in the fly brain. Curr. Biol. *bxs.
34. Yu, H.-H., Kao, C.-F., He, Y., Ding, P., Kao, J.-C., and Lee, T. (2010). A complete developmental sequence of a Drosophila neuronal lineage as revealed by twin-spot MARCM. PLoS Biol. *8*, e1000461.
35. Lin, S., Kao, C.-F., Yu, H.-H., Huang, Y., and Lee, T. (2012). Lineage analysis of Drosophila lateral antennal lobe neurons reveals notch-dependent binary temporal fate decisions. PLoS Biol. *10*, e1001425.
36. Tanaka, N.K., Endo, K., and Ito, K. (2012). Organization of antennal lobe-associated neurons in adult Drosophila melanogaster brain. J. Comp. Neurol. *520*, 4067–4130.
37. Ito, M., Masuda, N., Shinomiya, K., Endo, K., and Ito, K. (2013). Systematic analysis of neural projections reveals clonal composition of the Drosophila brain. Curr. Biol. *23*, 644–655.
38. Lee, T., Lee, A., and Luo, L. (1999). Development of the Drosophila mushroom bodies: sequential generation of three distinct types of neurons from a neuroblast. Development *126*, 4065–4076. https://www.ncbi.nlm.nih.gov/pubmed/10457015.
39. Aso, Y., Hattori, D., Yu, Y., Johnston, R.M., Iyer, N.A., Ngo, T.-T.B., Dionne, H., Abbott, L.F., Axel, R., Tanimoto, H., and Rubin, G.M. (2014). The neuronal architecture of the mushroom body provides a logic for associative learning. eLife *3*, e04577.
40. Vogt, K., Aso, Y., Hige, T., Knapek, S., Ichinose, T., Friedrich, A.B., Turner, G.C., Rubin, G.M., and Tanimoto, H. (2016). Direct neural pathways convey distinct visual information to Drosophila mushroom bodies. eLife *5*, e14009.
41. Helfrich-Förster, C., Shafer, O.T., Wülbeck, C., Grieshaber, E., Rieger, D., and Taghert, P. (2007). Development and morphology of the clock-gene-

expressing lateral neurons of Drosophila melanogaster. J. Comp. Neurol. *500*, 47–70.

42. Miyasako, Y., Umezaki, Y., and Tomioka, K. (2007). Separate sets of cerebral clock neurons are responsible for light and temperature entrainment of Drosophila circadian locomotor rhythms. J. Biol. Rhythms *22*, 115–126.
43. Shafer, O.T., Helfrich-Förster, C., Renn, S.C.P., and Taghert, P.H. (2006). Reevaluation of Drosophila melanogaster's neuronal circadian pacemakers reveals new neuronal classes. J. Comp. Neurol. *498*, 180–193.
44. Sekiguchi, M., Inoue, K., Yang, T., Luo, D.-G., and Yoshii, T. (2020). A Catalog of GAL4 Drivers for Labeling and Manipulating Circadian Clock Neurons in *Drosophila melanogaster*. J. Biol. Rhythms *35*, 207–213.
45. Goda, T., and Hamada, F.N. (2019). *Drosophila* Temperature Preference Rhythms: An Innovative Model to Understand Body Temperature Rhythms. Int. J. Mol. Sci. *20*, 1988.
46. Wang, Y., Wright, N.J., Guo, H., Xie, Z., Svoboda, K., Malinow, R., Smith, D.P., and Zhong, Y. (2001). Genetic manipulation of the odor-evoked distributed neural activity in the Drosophila mushroom body. Neuron *29*, 267–276.
47. Wang, Y., Chiang, A.-S., Xia, S., Kitamoto, T., Tully, T., and Zhong, Y. (2003). Blockade of neurotransmission in Drosophila mushroom bodies impairs odor attraction, but not repulsion. Curr. Biol. *13*, 1900–1904.
48. Mizunami, M., Nishino, H., and Yokohari, F. (2016). Status of and Future Research on Thermosensory Processing. Front. Physiol. *7*, 150.
49. Grabe, V., Baschwitz, A., Dweck, H.K.M., Lavista-Llanos, S., Hansson, B.S., and Sachse, S. (2016). Elucidating the Neuronal Architecture of Olfactory Glomeruli in the Drosophila Antennal Lobe. Cell Rep. *16*, 3401–3413.
50. Aimon, S., Katsuki, T., Jia, T., Grosenick, L., Broxton, M., Deisseroth, K., Sejnowski, T.J., and Greenspan, R.J. (2019). Fast near-whole-brain imaging in adult Drosophila during responses to stimuli and behavior. PLoS Biol. *17*, e2006732.
51. Huoviala, P., Dolan, M.-J., Love, F.M., Frechter, S., Roberts, R.J.V., Mitrevica, Z., Schlegel, P., Bates, A.S., Aso, Y., Rodrigues, T., et al. (2018). Neural circuit basis of aversive odour processing in Drosophila from sensory input to descending output. bioRxiv.
52. Gentile, C., Sehadova, H., Simoni, A., Chen, C., and Stanewsky, R. (2013). Cryptochrome antagonizes synchronization of Drosophila's circadian clock to temperature cycles. Curr. Biol. *23*, 185–195.
53. Li, P.H., Lindsey, L.F., Januszewski, M., Zheng, Z., Bates, A.S., Taisz, I., Tyka, M., Nichols, M., Li, F., Perlman, E., et al. (2019). Automated Reconstruction of a Serial-Section EM Drosophila Brain with Flood-Filling Networks and Local Realignment. bioRxiv. https://doi.org/10.1101/394403.
54. Thorne, N., and Amrein, H. (2008). Atypical expression of Drosophila gustatory receptor genes in sensory and central neurons. J. Comp. Neurol. *506*, 548–568.
55. Pfeiffer, B.D., Truman, J.W., and Rubin, G.M. (2012). Using translational enhancers to increase transgene expression in Drosophila. Proc. Natl. Acad. Sci. USA *109*, 6626–6631.
56. Port, F., Chen, H.-M., Lee, T., and Bullock, S.L. (2014). Optimized CRISPR/Cas tools for efficient germline and somatic genome engineering in Drosophila. Proc. Natl. Acad. Sci. USA *111*, E2967–E2976.
57. Diao, F., Ironfield, H., Luan, H., Diao, F., Shropshire, W.C., Ewer, J., Marr, E., Potter, C.J., Landgraf, M., and White, B.H. (2015). Plug-and-play genetic access to Drosophila cell types using exchangeable exon cassettes. Cell Rep. *10*, 1410–1421.
58. Dolan, M.-J., Frechter, S., Bates, A.S., Dan, C., Huoviala, P., Roberts, R.J., Schlegel, P., Dhawan, S., Tabano, R., Dionne, H., et al. (2019). Neurogenetic dissection of the *Drosophila* lateral horn reveals major outputs, diverse behavioural functions, and interactions with the mushroom body. eLife *8*, e43079.
59. Zheng, Z., Li, F., Fisher, C., Ali, I.J., Sharifi, N., Calle-Schuler, S., Hsu, J., Masoodpanah, N., Kmecova, L., Kazimiers, T., et al. (2020). Structured sampling of olfactory input by the fly mushroom body. bioRxiv. https://doi.org/10.1101/2020.04.17.047167.
60. Chiang, A.-S., Lin, C.-Y., Chuang, C.-C., Chang, H.-M., Hsieh, C.-H., Yeh, C.-W., Shih, C.-T., Wu, J.-J., Wang, G.-T., Chen, Y.-C., et al. (2011). Three-dimensional reconstruction of brain-wide wiring networks in Drosophila at single-cell resolution. Curr. Biol. *21*, 1–11.
61. Saalfeld, S., Cardona, A., Hartenstein, V., and Tomancak, P. (2009). CATMAID: collaborative annotation toolkit for massive amounts of image data. Bioinformatics *25*, 1984–1986.
62. Schneider-Mizell, C.M., Gerhard, S., Longair, M., Kazimiers, T., Li, F., Zwart, M.F., Champion, A., Midgley, F.M., Fetter, R.D., Saalfeld, S., and Cardona, A. (2016). Quantitative neuroanatomy for connectomics in Drosophila. eLife *5*, e12059. https://doi.org/10.7554/eLife.12059.
63. Bates, A.S., Manton, J.D., Jagannathan, S.R., Costa, M., Schlegel, P., Rohlfing, T., and Jefferis, G.S.X.E. (2014). The natverse: a versatile computational toolbox to combine and analyse neuroanatomical data. bioRxiv. https://doi.org/10.1101/006353.
64. Schlegel, P., Texada, M.J., Miroschnikow, A., Schoofs, A., Hückesfeld, S., Peters, M., Schneider-Mizell, C.M., Lacin, H., Li, F., Fetter, R.D., et al. (2016). Synaptic transmission parallels neuromodulation in a central food-intake circuit. eLife *5*, e16799.
65. Ni, L., Klein, M., Svec, K.V., Budelli, G., Chang, E.C., Ferrer, A.J., Benton, R., Samuel, A.D., and Garrity, P.A. (2016). The Ionotropic Receptors IR21a and IR25a mediate cool sensing in Drosophila. eLife *5*, e13254.
66. Prokop, A., and Meinertzhagen, I.A. (2006). Development and structure of synaptic contacts in Drosophila. Semin. Cell Dev. Biol. *17*, 20–30.
67. Frechter, S., Bates, A.S., Tootoonian, S., Dolan, M.-J., Manton, J., Jamasb, A.R., Kohl, J., Bock, D., and Jefferis, G. (2019). Functional and anatomical specificity in a higher olfactory centre. eLife *8*, e44590.

STAR★METHODS

KEY RESOURCES TABLE

REAGENT or RESOURCE	SOURCE	IDENTIFIER
Antibodies		
mouse anti-nc82 (1:200)	DSHB, University of Iowa	Bruchpilot; RRID:AB_2314866
chicken anti-GFP (1:100)	Avēs Labs Inc.	Cat#: GFP-1010; RRID:AB_10000240
rabbit anti-DsRED (1:200)	Takara Bio	Cat#: 632392; RRID:AB_10013483
goat anti-mouse Cy5 (1:200)	Jackson ImmunoResearch Labs	Cat#: 115-175-166; RRID:AB_2338714
goat anti-chicken 488 (1:200)	Invitrogen	Cat#: A-11039; RRID:AB_142924
goat anti-rabbit Cy3 (1:200)	Jackson ImmunoResearch Labs	Cat#: #115-165-166; RRID:AB_2338692
Experimental Models: Organisms/Strains		
Ir40a-LexA	[25]	N/A
Gr28b.d-Gal4	[54]	N/A
UAS-myr:GFP (P[10UAS-IVS-myr::GFP]attP1)	[55]	N/A
LexAop-mRFP	Bloomington Stock Center	Stock# 29956; RRID:BDSC_29956
Ir21a-T2A-Gal4	This paper	N/A
Ir68a-T2A-Gal4	This paper	N/A
yw, 3XP3-EGFP, Vasa-Cas9 attp18	Kate Koles, Rodal Lab Brandeis University	N/A
Oligonucleotides		
gRNA against Ir21a intronic region: GCGCGTGAGTATTGCTTAAT	This paper	N/A
gRNA against Ir68a intronic region: GTGTGATATGAAAAGCATTC	This paper	N/A
Primer 1: Ir21a 5′ homology arm: atacgactcactatagggcgacccaccggtCGATATG TCATATTATTGGGTAGCTCTGGT	This paper	N/A
Primer 2: Ir21a 5′ homology arm: ccgaaaaccgcttctgacctggggcggccgcAA GCAATACTCACGCGCAT	This paper	N/A
Primer 3: Ir21a 3′ homology arm: ccgaaaaccgcttctgacctgggggcgcgccAATAGG GATACGTTTTTGTAACAAATAATGCGCTT CACACAGGAG	This paper	N/A
Primer 4: Ir21a 3′ homology arm: aagggaacctccccactagtggtaccCTCAAATGA AGCGCCGATCGG	This paper	N/A
Primer 5: Ir68a 5′ homology arm: atacgactcactatagggcgacccaccggtGGAAC CTCTTTGCCAGTTGCC	This paper	N/A
Primer 6: Ir68a 5′ homology arm: ccgaaaaccgcttctgacctggggcggccgcTGCTT TTCATATCACACTAGATTATTTTG	This paper	N/A
Primer 7: Ir68a 3′ homology arm: ccgaaaaccgcttctgacctgggggcgcgccTTCC GGCGACTTAATGGCTTTGT	This paper	N/A
Primer 8: Ir68a 3′ homology arm: aagggaacctccccactagtggtaccCTTCTAAAG AGATGGCCAAGCAAAAGC	This paper	N/A
Recombinant DNA		
pCFD3-dU6:3gRNA	[56]	N/A
pGEM (phase 0)	[57]	N/A

(Continued on next page)

Continued

REAGENT or RESOURCE	SOURCE	IDENTIFIER
Deposited Data		
The Full Adult Fly Brain ssTEM dataset	[30]	https://temca2data.org/
FAFB manual neuronal reconstructions	This paper [30, 33, 51, 58, 59],	https://fafb.catmaid.virtualflybrain.org/ Neurons can be retrieved by their SKID (skeleton identifier).
Data S1 Skeletons and meshes	This paper [30, 33, 51, 58, 59],	https://doi.org/10.5281/zenodo.3879033
FlyCircuit 1.2 database of *Drosophila* brain neurons	[60]	http://www.flycircuit.tw/
Fly Light GAL4/LexA collection	[26]	http://flweb.janelia.org/cgi-bin/flew.cgi
Software and Algorithms		
CATMAID	[61, 62]	https://github.com/catmaid/CATMAID
natverse, including the nat, nat.flybrains, elmr, tracerutils, flycircuit, and rcatmaid packages	[63]	https://github.com/natverse/
NBLAST algorithm and R package	[31, 63]	https://github.com/natverse/nat.nblast
Pymaid, python code for interacting with CATMAID	[33]	https://github.com/schlegelp/pymaid
FAFBseg, python code for working with the partial autosegmentation of FAFB from [53]	[33]	https://github.com/flyconnectome/fafbseg-py
CATMAID-to-Blender	[64]	https://github.com/schlegelp/CATMAID-to-Blender
rmushroom package	[30]	https://github.com/flyconnectome/rmushroom

RESOURCE AVAILABILITY

Lead Contact

All queries regarding EM reconstructions, software and algorithms should be directed to the Lead Contact, Gregory Jefferis (jefferis@mrc-lmb.cam.ac.uk).

Materials Availability

All plasmids and fly strains generated in this study are freely available upon request. Please contact Paul A. Garrity (pgarrity@brandeis.edu).

Data and Code Availability

All neuron reconstructions described in this study will be uploaded to a public CATMAID instance hosted by Virtual Fly Brain (https://fafb.catmaid.virtualflybrain.org/) following publication. Additionally, the skeletons and meshes have been deposited in Zenodo: https://doi.org/10.5281/zenodo.3879033. The full source code and documentation for natverse packages is available at https://github.com/natverse/ and http://natverse.org/.

EXPERIMENTAL MODEL AND SUBJECT DETAILS

Standard *Drosophila* husbandry techniques were used for the maintenance and propagation of flies. For immunohistochemistry, flies were raised at 25°C on standard food.

METHOD DETAILS

Drosophila Strains and Driver Line Generation

Prior to this publication, Ir21a- and Ir68a-Gal4 drivers were available, but neither one portrayed complete expression pattern of their respective genes as these lines were made by making a guess about what sequences that might comprise a gene's regulatory region (e.g., 5′ sequences) and placing those sequences upstream of Gal4. The Ir21a(putative promoter)-Gal4 expression in the larva was described previously [65]. Unfortunately, that construct lacks key regulatory information and does not express in the adult. The construction and expression of the Ir68a(putative promoter)-Gal4 was also previously described [20]. That Gal4 drives very weak expression in the adult and can only be detected in sacculus chamber II. For these reasons we sought to generate new drivers using the T2A-Gal4 knock-in strategy that would provide a more complete view of these genes' expression patterns.

Ir21a-T2A-Gal4 and Ir68a-T2A-Gal4 were generated using techniques previously described [57]. Briefly, intronic regions in the Ir21a and Ir68a genes were targeted via homology directed repair by injecting two plasmids, one containing gRNA (Ir21a: 5′-GCGCGTGAGTATTGCTTAAT; Ir68a: 5′-GTGTGATATGAAAAGCATTC) and another with T2A-Gal4 3XP3-RFP at phase0 (flanked by homology arms; Ir21a: 5′arm primers 1-2, 3′arm primers 3-4; Ir68a: 5′arm: primers 5-6, 3′arm primers 7-8) into embryos carrying Vasa-Cas9 3XP3-EGFP (kindly shared by Kate Koles and Rodal Lab, Brandeis University).

Immunohistochemistry

For brain dissections, whole flies were fixed for 2 h in 4% paraformaldehyde while rotating and washed several times prior to dissections. The dissected brains were incubated at room temperature in blocking solution (10% normal goat serum) for one h. Primary and secondary antibody incubations were done at 4°C for 48 h each. Washing between antibody incubations was performed over 24 h at 4°C.

Third antennal sections were dissected, fixed in 4% paraformaldehyde for 1 h on ice, and incubated in blocking solution for 1 h at room temperature. Primary and secondary antibody incubations were done at 4°C for 24 h each while rotating. Washing between antibody incubations were performed at room temperature 3-4 times with PBS-Tx (PBS with 0.5% Triton X).

IHC Image Acquisition

Imaging of antennae and the antennal lobe were performed using a Zeiss LSM 880 confocal microscope with a 63X oil lens. Maximum intensity z stack projections were made from z stack sections taken every 1μm.

Sparse EM Reconstruction

Neuron skeletons were traced in a full adult female *Drosophila* brain ssTEM (serial section transmission electron microscope) volume (FAFB, https://fafb.catmaid.virtualflybrain.org/, http://temca2data.org), either manually as previously reported [30] or by automated segmentation, using a modified version of CATMAID (http://www.catmaid.org) [61, 62]. Skeleton fragments that had been automatically segmented with the flood-filling technique [53] were manually joined in a separate CATMAID instance, imported into the manual CATMAID instance, and merged with manually traced partial skeletons where possible (https://github.com/flyconnectome/fafbseg-py). Skeletons were either traced to identification (with soma, backbone, and main branches) or to completion (with all twigs, including pre- and postsynapses, and fully reviewed) [33].

Chemical synapses were annotated based on previously described criteria: thick, dark active zone, presynaptic membrane specializations (T-bars, vesicles), and a synaptic cleft [66]. We scored each continuous synaptic cleft as a single presynapse and opposing neuronal membranes in contact with that synaptic cleft as associated postsynapses.

Analysis and Representation of Traced Skeletons

R packages from the natverse collection (http://natverse.org/) [63] were used to plot traced skeletons and analyze their morphology. Custom R and Python scripts were used to perform additional analysis, as described below.

Neuron Clustering via NBLAST

The nat.nblast R package (https://github.com/natverse/nat.nblast) was used to compare neuron skeletons by morphology and position and generate a hierarchical clustering with Ward's method of the neurons within each group (e.g., VP PNs), providing a method for identifying neuron types [31].

Light versus EM Comparisons of Neuron Morphology

To assist in the identification of our reconstructed neurons, we used nat.nblast to compare them with segmentations of annotated PNs in the light-level FlyCircuit database [31, 60] (http://www.flycircuit.tw/). We used linear and then non-rigid transformations to bring neurons from FAFB into FCWB (FlyCircuit whole brain) space, then performed an all-by-all NBLAST to look for the closest matches. Neurons that did not have clear matches in FlyCircuit could sometimes be manually identified by comparing morphology to published data. Sparse driver lines predicted to label our reconstructed neurons were identified in a similar way through comparisons with the Fly Light database (http://flweb.janelia.org/cgi-bin/flew.cgi) [26].

Neuronal Nomenclature

Sensory neurons were typed by the AL glomerulus they innervated. Projection neurons were typed according to their AL glomerular innervation, AL tract, and neuroblast lineage [33, 37] and their likely neurotransmitter types assigned by tract [33, 36]. Kenyon cells were typed according to their positions in the peduncle and the MB axon lobes they innervated [39]. Lateral horn neurons were typed based on their primary neurite tracts and areas of innervation, according to previously established conventions [67]. Numbers were added to names with # when multiple individuals were assigned the same type.

QUANTIFICATION AND STATISTICAL ANALYSIS

Analysis of Biglomerular PN Inputs

A best fit line to a linear regression model showing correlation of cable length in each glomerulus with RN input was plotted in R using the lm() function. This function provided the Multiple R-squared and p values (Figure 3H).

Analysis of lACA Circuit Connectivity

Meshes were made in Blender from the completed axons and synapses of the VP3 vPN and VP2 adPN, which ramify throughout the lACA, and imported into CATMAID as lACA volumes. All PNs with presynapses in these lACA volumes were identified with the CATMAID connectivity widget and designated as lACA PNs. We manually annotated all postsynapses downstream of these VP3 vPN and VP2 adPN, which together contribute > 90% of lACA presynapses. These downstream postynapses were then randomly sampled and the corresponding skeletons reconstructed to identification. We continued sampling until the 10 most recent novel hits featured fewer than five connections with the query neuron (Figures S5D–S5D''), corresponding to 21 - 30% of postsynapses for each PN. All identified neurons were reconstructed in the lACA, and those with at least 5 connections to lACA PNs were designated as lACA circuit neurons. We reconstructed 42 sampled neurons within the lACA to determine their inputs, then completely reconstructed 20 (47.6%) of these, including representatives of all morphological types receiving more than 15 lACA PN inputs. Input PNs and target neurons of the same type were pooled, and adjacency matrices were constructed in CATMAID using the Graph widget. For simplicity, a few neurons in the adjacency matrices were omitted from the circuit diagrams (Figures 5L and 5M).

Analysis of lACA-associated Kenyon Cell Inputs

The dendrites of KCs that shared 5 or more synapses with lACA-associated PNs within the lACA volume on the RHS were reconstructed in CATMAID, and their postsynapses were manually associated with upstream neurons - mainly antennal lobe PNs which had already been traced to identification [30, 33]. Analysis of KC inputs was performed in R with a customised script, utilizing the nat and elmr packages from the natverse. KC skeletons were bridged from FAFB space into the JFRC2 template [26] (http://www.virtualflybrain.org/) using the nat.flybrains package (natverse) and then pruned with the split_neuron_local() function in the tracerutils package (https://flyconnectome.github.io/tracerutils/) to include only dendrites (branches off the main tract in the calyx). For each KC branch, all upstream PNs contributing at least 5 synapses were identified and weighted equally as inputs observed. (Non-PN partners and inputs from lACA-associated PNs within the lACA volume were omitted from further analysis.)

Expected numbers of inputs representing each AL glomerulus were based on the total number of boutons reported [30], or newly identified, for each PN class. PN boutons were manually annotated and extracted using the rmushroom package (https://github.com/zhihaozheng). A virtual KC population with the same number of KCs and PN inputs was created by random draw, with the probability of each input based on the percentage share of associated PN boutons. This was repeated 1000 times to generate an average number of expected inputs for the population (Figures 4L and 4M). Inputs from each glomerulus were assigned a putative valence based on the olfactory literature [33].

Current Biology, Volume *30*

Supplemental Information

Connectomics Analysis Reveals First-, Second-, and Third-Order Thermosensory and Hygrosensory Neurons in the Adult *Drosophila* Brain

Elizabeth C. Marin, Laurin Büld, Maria Theiss, Tatevik Sarkissian, Ruairí J.V. Roberts, Robert Turnbull, Imaan F.M. Tamimi, Markus W. Pleijzier, Willem J. Laursen, Nik Drummond, Philipp Schlegel, Alexander S. Bates, Feng Li, Matthias Landgraf, Marta Costa, Davi D. Bock, Paul A. Garrity, and Gregory S.X.E. Jefferis

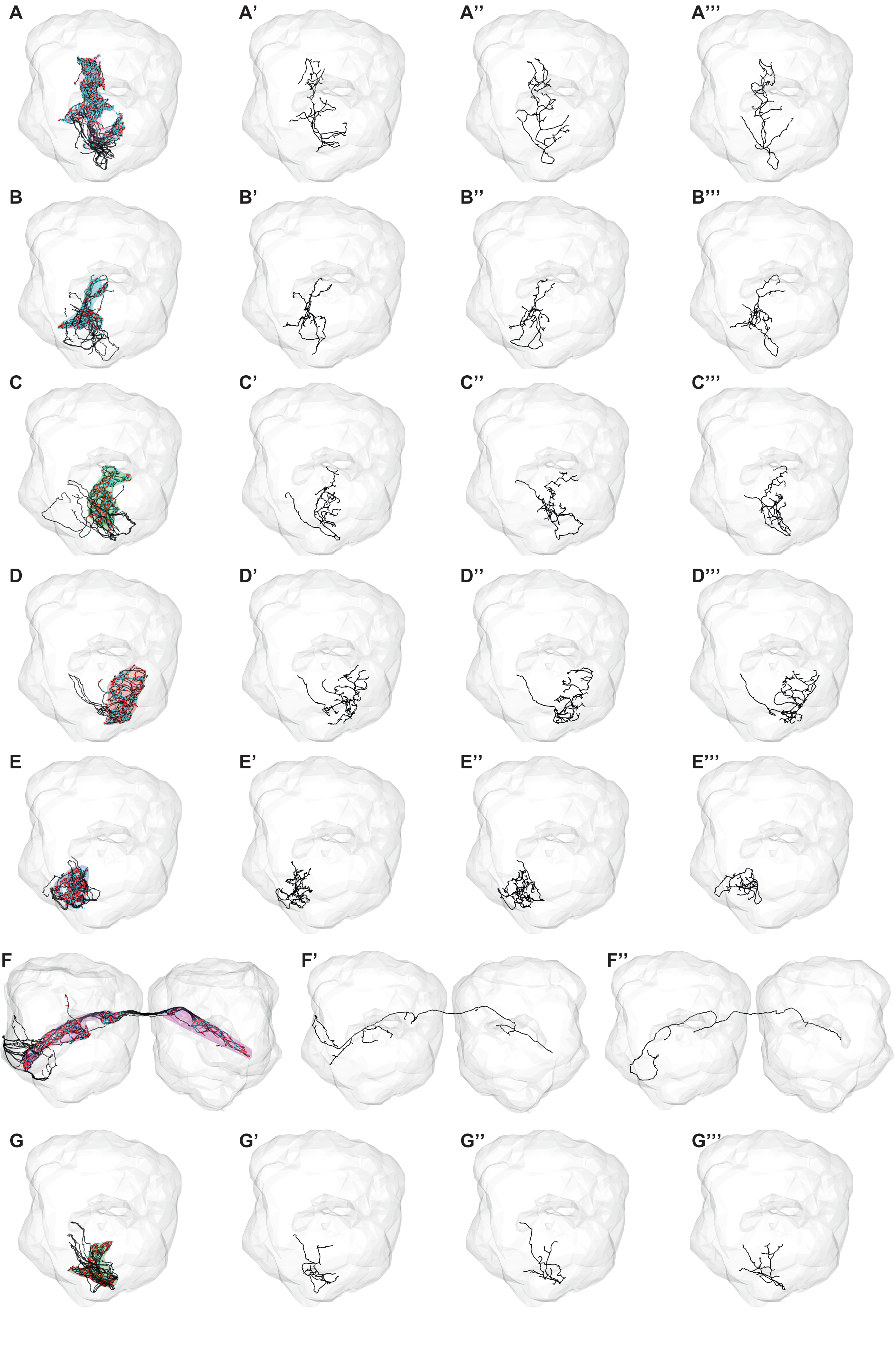
A
A'
A''
A'''
B
B'
B''
B'''
C
C'
C''
C'''
D
D'
D''
D'''
E
E'
E''
E'''
F
F'
F''
G
G'
G''
G'''

Figure S1. Individual reconstructions reveal morphological stereotypy for seven distinct classes of VP sensory neurons, related to Figure 1. Right antennal lobe, frontal view. Reconstructed neurons in black, with presynaptic sites in red and postsynaptic sites in cyan. **A-A’’’.** Full population (9) and three completed examples of VP1d RNs. **B-B’’’.** Full population (6) and three completed examples of VP1l RNs. **C-C’’’.** Full population (6) and three completed examples of VP1m RNs. **D-D’’’.** Full population (3) and three completed examples of VP2 RNs. **E-E’’’.** Full population (4) and three completed examples of VP3 RNs. **E’’’** depicts VP3RNbr#1 (the simpler, suspected non-aristal, RN). **F-F’’’.** Full population (14) and two completed examples of VP4 RNs. **G-G’’’.** Full population (9) and three completed examples of VP5 RNs.

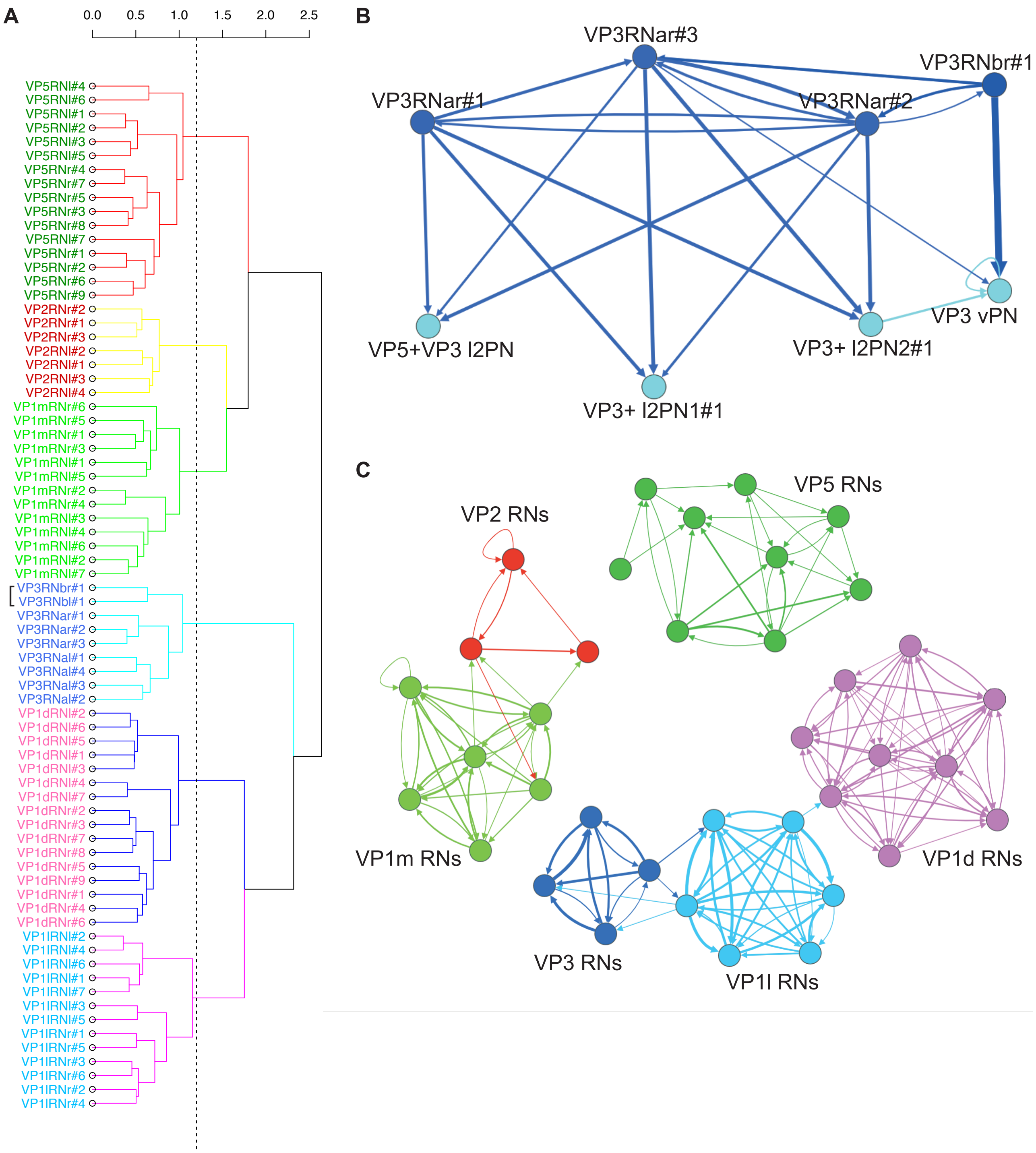
A
0.0
0.5
1.0
1.5
2.0
2.5
VP5RNl#4
VP5RNl#6
VP5RNl#1
VP5RNl#2
VP5RNl#3
VP5RNl#5
VP5RNr#4
VP5RNr#7
VP5RNr#5
VP5RNr#3
VP5RNr#8
VP5RNl#7
VP5RNr#1
VP5RNr#2
VP5RNr#6
VP5RNr#9
VP2RNr#2
VP2RNr#1
VP2RNr#3
VP2RNl#2
VP2RNl#1
VP2RNl#3
VP2RNl#4
VP1mRNr#6
VP1mRNr#5
VP1mRNr#1
VP1mRNr#3
VP1mRNl#1
VP1mRNl#5
VP1mRNr#2
VP1mRNr#4
VP1mRNl#3
VP1mRNl#4
VP1mRNl#6
VP1mRNl#2
VP1mRNl#7
VP3RNbr#1
VP3RNbl#1
VP3RNar#1
VP3RNar#2
VP3RNar#3
VP3RNal#1
VP3RNal#4
VP3RNal#3
VP3RNal#2
VP1dRNl#2
VP1dRNl#6
VP1dRNl#5
VP1dRNl#1
VP1dRNl#3
VP1dRNl#4
VP1dRNl#7
VP1dRNr#2
VP1dRNr#3
VP1dRNr#7
VP1dRNr#8
VP1dRNr#5
VP1dRNr#9
VP1dRNr#1
VP1dRNr#4
VP1dRNr#6
VP1lRNl#2
VP1lRNl#4
VP1lRNl#6
VP1lRNl#1
VP1lRNl#7
VP1lRNl#3
VP1lRNl#5
VP1lRNr#1
VP1lRNr#5
VP1lRNr#3
VP1lRNr#6
VP1lRNr#2
VP1lRNr#4
B
VP3RNar#3
VP3RNbr#1
VP3RNar#1
VP3RNar#2
VP3 vPN
VP5+VP3 l2PN
VP3+ l2PN2#1
VP3+ l2PN1#1
C
VP5 RNs
VP2 RNs
VP1m RNs
VP1d RNs
VP3 RNs
VP1l RNs

Figure S2. There are seven distinct populations of thermosensory and hygrosensory neurons, related to Figure 1. A. Morphological hierarchical clustering of the unilateral sensory neurons from both hemispheres. Cut at height 1.2 (dashed line) which groups the RNs by type. Neuron names are colour-coded by glomerulus. Bracket: simple VP3 TRNs. **B-C.** Connectivity graphs for thermo- and hygrosensory neuron populations. Arrow directions and thicknesses reflect direction of information flow and numbers of synaptic connections. Each circle (node) represents one neuron, colour-coded by class. **B.** The simpler VP3 TRN (VP3RNbr#1) provides input to the “slow-cool” VP3 vPN but not to VP5+VP3 lPN, VP3+ lPN1#1, or VP3+ lPN2#1. VP3RNar#1-3: remaining three (putative aristal) VP3 TRNs. Edges of <3 connections have been omitted for visual clarity. **C.** VP1d, VP1l, and VP1m sensory neurons constitute three separate populations.

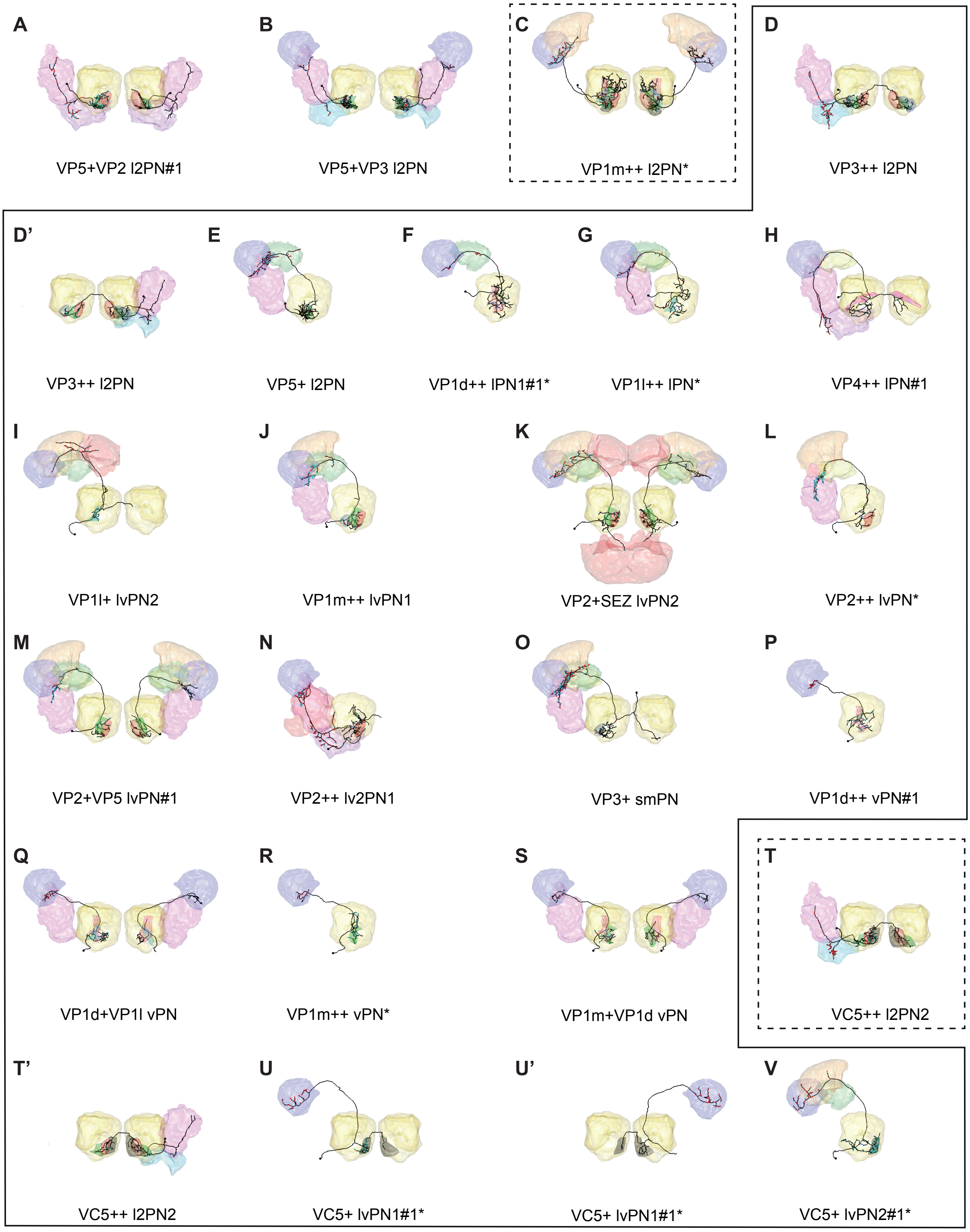
A
VP5+VP2 l2PN#1
B
VP5+VP3 l2PN
C
VP1m++ l2PN*
D
VP3++ l2PN
D'
VP3++ l2PN
E
VP5+ l2PN
F
VP1d++ lPN1#1*
G
VP1l++ lPN*
H
VP4++ lPN#1
I
VP1l+ lvPN2
J
VP1m++ lvPN1
K
VP2+SEZ lvPN2
L
VP2++ lvPN*
M
VP2+VP5 lvPN#1
N
VP2++ lv2PN1
O
VP3+ smPN
P
VP1d++ vPN#1
Q
VP1d+VP1l vPN
R
VP1m++ vPN*
S
VP1m+VP1d vPN
T
VC5++ l2PN2
T'
VC5++ l2PN2
U
VC5+ lvPN1#1*
U'
VC5+ lvPN1#1*
V
VC5+ lvPN2#1*

Figure S3. Additional types of putative thermo- and hygrosensory PNs, related to Table 1. Frontal views of reconstructions (black) of additional candidate thermo- and hygrosensory PNs that innervate VP glomeruli (colour-coded as in Figure 1B-B', non-VP glomeruli in grey). Presynaptic sites in red and postsynaptic sites in cyan. Brain neuropils colour-coded as in Figure 2C. Asterisks: primarily olfactory PNs. Black outline: candidate novel thermosensory PNs. Dashed outline: PNs previously described but not recognised as VP/thermosensory, or associated with the wrong glomeruli.

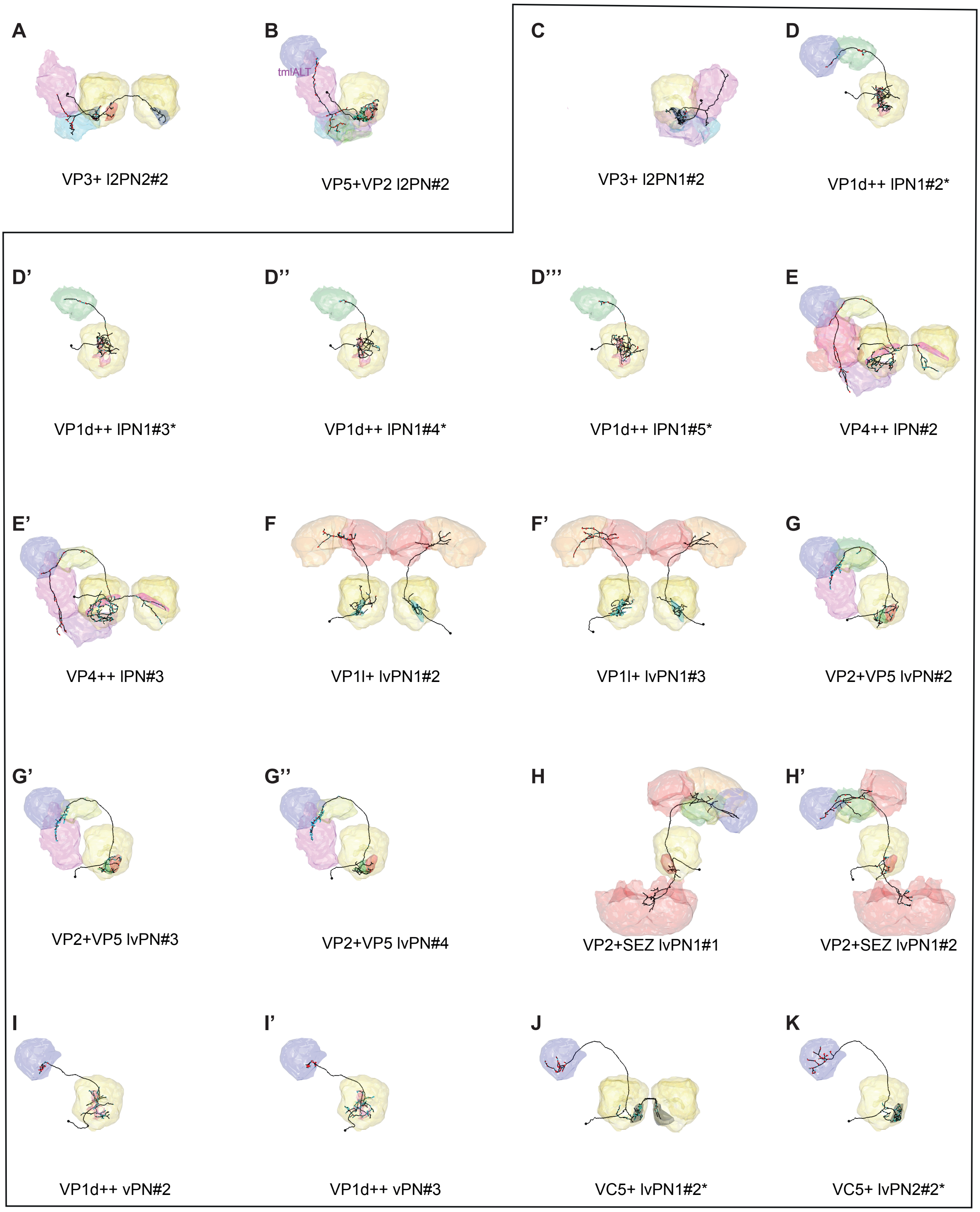
A
VP3+ l2PN2#2
B
tmlALT
VP5+VP2 l2PN#2
C
VP3+ l2PN1#2
D
VP1d++ lPN1#2*
D’
VP1d++ lPN1#3*
D’’
VP1d++ lPN1#4*
D’’’
VP1d++ lPN1#5*
E
VP4++ lPN#2
E’
VP4++ lPN#3
F
VP1l+ lvPN1#2
F’
VP1l+ lvPN1#3
G
VP2+VP5 lvPN#2
G’
VP2+VP5 lvPN#3
G’’
VP2+VP5 lvPN#4
H
VP2+SEZ lvPN1#1
H’
VP2+SEZ lvPN1#2
I
VP1d++ vPN#2
I’
VP1d++ vPN#3
J
VC5+ lvPN1#2*
K
VC5+ lvPN2#2*

Figure S4. Extra examples of putative thermo- and hygrosensory PN types, related to Table 1. Frontal views of reconstructions (black) of additional candidate thermo- and hygrosensory PNs that innervate VP glomeruli (colour-coded as in Figure 1B-B', non-VP glomeruli in grey). Presynaptic sites in red and postsynaptic sites in cyan. Brain neuropils colour-coded as in Figure 2C. Asterisks: primarily olfactory PNs. Black outline: candidate novel thermosensory PNs.

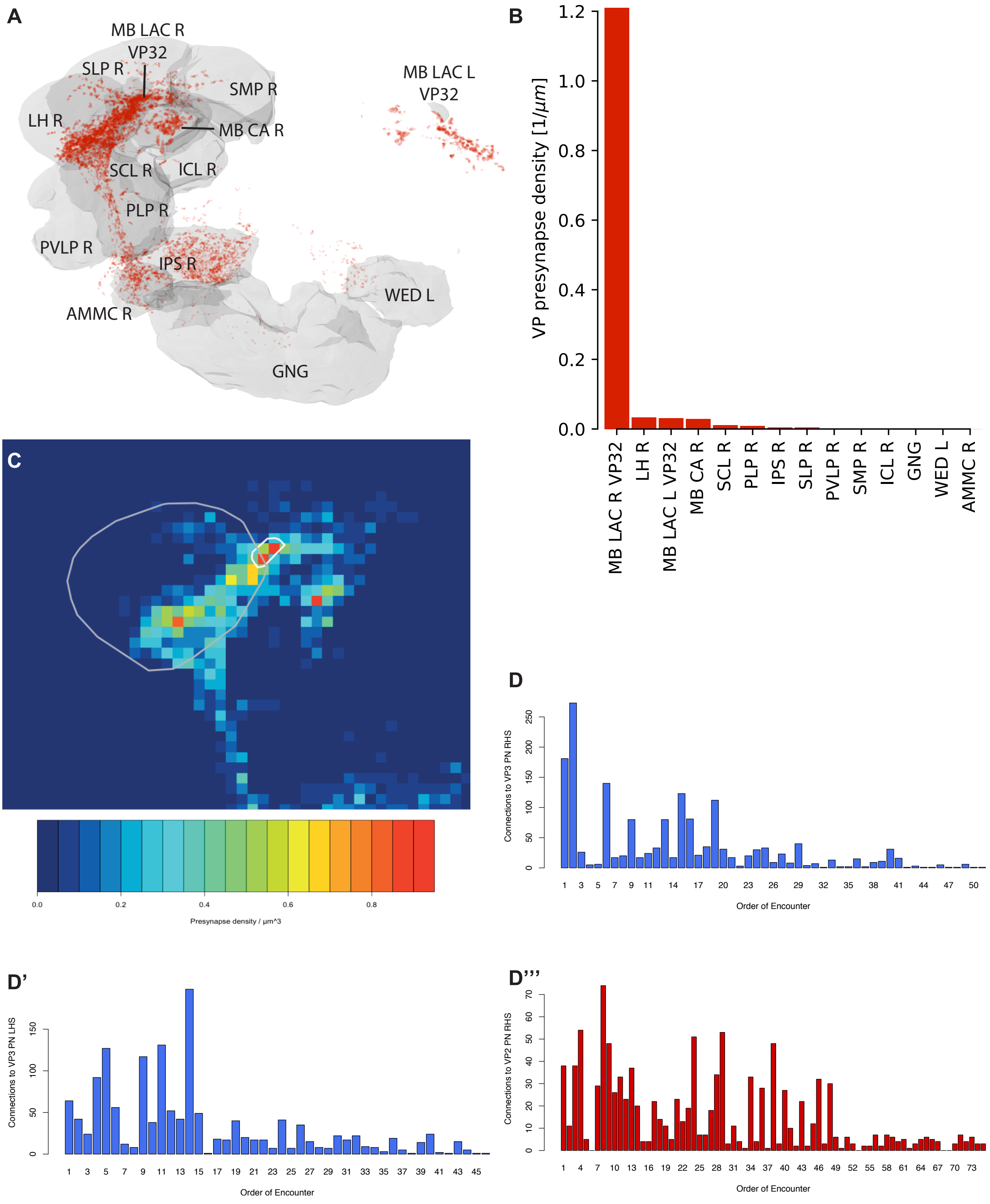
A
MB LAC R
VP32
SLP R
SMP R
LH R
MB CA R
SCL R
ICL R
PLP R
PVLP R
IPS R
AMMC R
GNG
WED L
MB LAC L
VP32
B
VP presynapse density [1/μm]
1.2
1.0
0.8
0.6
0.4
0.2
0.0
MB LAC R VP32
LH R
MB LAC L VP32
MB CA R
SCL R
PLP R
IPS R
SLP R
PVLP R
SMP R
ICL R
GNG
WED L
AMMC R
C
0.0
0.2
0.4
0.6
0.8
Presynapse density / μm^3
D
Connections to VP3 PN RHS
Order of Encounter
D'
Connections to VP3 PN LHS
Order of Encounter
D'''
Connections to VP2 PN RHS
Order of Encounter

Figure S5. Random sampling to define circuitry in the lACA, the neuropil most densely targeted by VP PNs, related to Figure 4. A. Presynapses of all RHS AL VP PNs, plotted with neuropil volumes. AMMC R: RHS antennal mechanosensory and motor centre. MB CA R: RHS mushroom body calyx. ICL R: RHS inferior clamp. IPS R: RHS inferior posterior slope. LH: RHS lateral horn. MB LAC R VP32: RHS lateral accessory calyx. MB LAC L VP32: LHS lateral accessory calyx. PLP R: RHS posterior lateral protocerebrum. PVLP R: RHS posterior ventral protocerebrum. SCL R: RHS superior clamp. GNG: suboesophageal zone. SLP R: RHS superior lateral protocerebrum. SMP R: RHS superior medial protocerebrum. WED L: LHS wedge. **B.** VP PN presynapse density plotted in each neuropil (abbreviations as in A). **C.** VP PN presynapse density (presynapses/ cubic micron), with LH and lACA outlined in white. **D-D''.** Histograms depicting connection strength of recovered novel hits from successive sampling of randomised postsynaptic profiles downstream of the RHS and LHS VP3 PN and the RHS VP2 PN in the lACA.

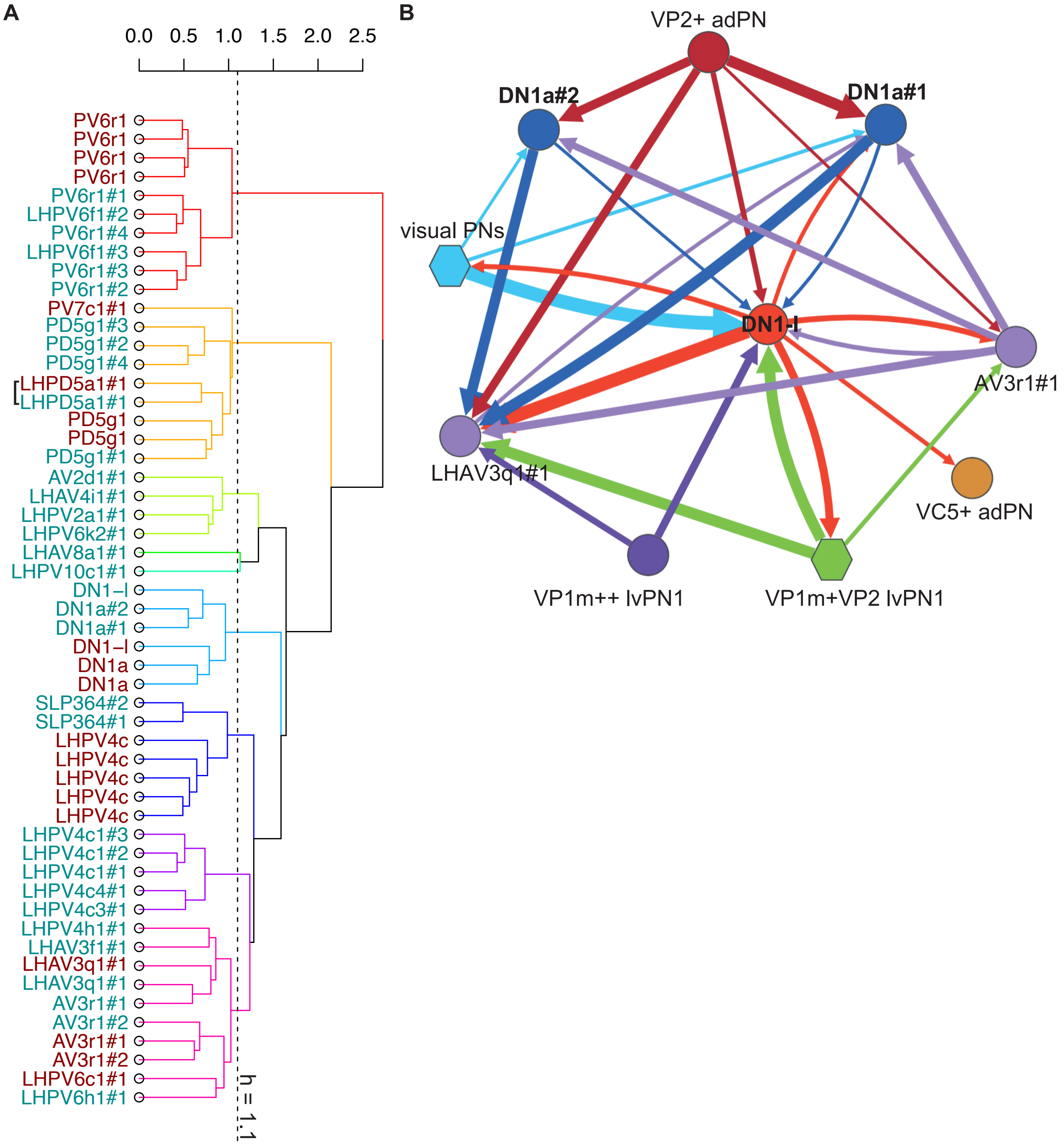
A
B
0.0 0.5 1.0 1.5 2.0 2.5
PV6r1
PV6r1
PV6r1
PV6r1
PV6r1#1
LHPV6f1#2
PV6r1#4
LHPV6f1#3
PV6r1#3
PV6r1#2
PV7c1#1
PD5g1#3
PD5g1#2
PD5g1#4
LHPD5a1#1
LHPD5a1#1
PD5g1
PD5g1
PD5g1#1
AV2d1#1
LHAV4i1#1
LHPV2a1#1
LHPV6k2#1
LHAV8a1#1
LHPV10c1#1
DN1–l
DN1a#2
DN1a#1
DN1–l
DN1a
DN1a
SLP364#2
SLP364#1
LHPV4c
LHPV4c
LHPV4c
LHPV4c
LHPV4c
LHPV4c1#3
LHPV4c1#2
LHPV4c1#1
LHPV4c4#1
LHPV4c3#1
LHPV4h1#1
LHAV3f1#1
LHAV3q1#1
LHAV3q1#1
AV3r1#1
AV3r1#2
AV3r1#1
AV3r1#2
LHPV6c1#1
LHPV6h1#1
h = 1.1
VP2+ adPN
DN1a#2
DN1a#1
visual PNs
DN1-l
AV3r1#1
LHAV3q1#1
VC5+ adPN
VP1m++ lvPN1
VP1m+VP2 lvPN1

Figure S6. Non-Kenyon cell IACA circuit neuron stereotypy and connectivity, related to Figure 5. A. Morphological hierarchical clustering of non-KC IACA neurons using NBLAST. Cut at height 1.1 (dashed line) to retrieve groups by neuron type. Dark cyan: RHS targets. Dark red: LHS targets. Square brackets: unique neurons matched across hemispheres. **B.** Connectivity graph showing that DN1a#1 and DN1a#2 have similar connectivity, distinct from that of DN1-I. Each circle (node) represents one neuron and each hexagon represents multiple pooled neurons of the same modality. Arrow directions and thicknesses reflect direction of information flow and numbers of synaptic connections. Edges of <10 connections and the VP3 vPN and VP2 adPN have been omitted for clarity.

SKID	NAME	SIDE	VP3	VP2	LACA	LP	VP	OTHER
5816884	KCg-s2R	RHS	138	48	187	9	11	AV3f2#1, DN1-l, mPN, visual
4181431	tKCa'b'R#1	RHS	26	48	75	0	0	NA
5824701	tKCa'b'R#2	RHS	33	34	67	0	0	NA
6279367	tKCa'b'R#3	RHS	31	32	63	0	0	NA
8799774	KCa'b'R#1	RHS	31	22	53	0	0	VL2p
4182914	KCa'b'R#2	RHS	23	38	61	0	0	DC1
8139894	KCa'b'R#3	RHS	24	30	57	0	20	NA
5824641	KCa'b'R#4	RHS	15	38	53	0	0	V, DL2d
5832021	KCa'b'R#5	RHS	16	26	43	16	0	(DA4l)
8821288	KCa'b'R#6	RHS	17	27	45	11	0	NA
5813673	KCa'b'R#7	RHS	13	33	49	0	0	VC1
4180842	KCa'b'R#8	RHS	17	21	41	5	0	DA2
478805	KCa'b'R#9	RHS	9	23	32	0	0	DP1l
16274	KCa'b'R#10	RHS	2	5	7	4	31	VL2p
175027	KCa'b'R#11	RHS	1	4	11	31	11	NA
8808581	KCa'b'R#12	RHS	0	4	7	18	0	DP1m, (VL2a), (DC1)
9117955	KCa'b'R#13	RHS	0	0	11	14	0	NA
9210	KCgR#1	RHS	0	3	17	25	0	DC1, DP1l, VA3, VA7l, VL2a
5459431	KCgR#2	RHS	1	6	7	1	0	VC3m, DL5, DA4l
8808541	KCgR#3	RHS	2	5	15	16	6	VL2a, DA2, VP1m + VP5
8808887	KCgR#4	RHS	0	4	6	15	0	DL2d, VC3m, DA4l, DM4
8822544	KCgR#5	RHS	1	3	19	8	0	VC3l, DL2v
8142	KCgR#6	RHS	1	3	6	27	0	DM4, DP1l, VA4, DM3, V
10228816	KCgR#7	RHS	0	0	14	13	0	DC1, DP1m, VC3m
10586365	KCgR#8	RHS	0	0	5	5	0	DC1, (VC1)
8361575	KCgR#9	RHS	0	0	10	4	20	(DA2), DA4l, DM5
36909	KCgR#10	RHS	1	1	7	23	0	DP1l, VA6, VC3m
10527316	KCgR#11	RHS	1	0	6	13	0	DC1, DP1m, V, VC3m
8798	KCg-s1R	RHS	4	0	4	3	0	(AV3f2#1), visual
8853436	KCg-s2L	LHS	92	18	111	6	3	AV3f2#1, (mPN), visual
8854339	tKCa'b'L#1	LHS	41	8	51	1	0	NA
8880337	tKCa'b'L#2	LHS	24	18	42	0	0	NA
8884784	tKCa'b'L#3	LHS	17	14	31	0	0	NA
8867359	KCa'b'L#1	LHS	38	19	57	3	0	VL2p
8885938	KCa'b'L#2	LHS	42	6	48	0	0	(VA7l)
8851349	KCa'b'L#3	LHS	15	23	38	0	0	(DP1m), (VM1)
8866744	KCa'b'L#4	LHS	22	14	36	0	5	NA
8869397	KCa'b'L#5	LHS	20	12	37	8	0	NA
8848927	KCa'b'L#6	LHS	17	11	28	0	0	(DP1l)
8877321	KCa'b'L#7	LHS	24	4	28	6	0	VM1
8847299	KCa'b'L#8	LHS	7	19	28	9	0	V
8870427	KCa'b'L#9	LHS	8	15	23	14	0	(VM1)
8864744	KCa'b'L#10	LHS	14	6	21	10	0	NA

8869803	KCa'b'L#11	LHS	8	8	17	12	4	NA
8865424	KCa'b'L#12	LHS	5	7	13	10	5	NA
9942769	KCgL#1	LHS	12	6	32	4	0	DC2, DM3, VA5, (VM3)
8865505	KCgL#2	LHS	3	0	6	12	0	DL2v, DM4, DM5, DP1m
8865603	KCgL#3	LHS	1	2	10	17	0	VA3, VM2, VL2p

Table S1. Table of lACA-associated Kenyon cells and their inputs, related to Figure 4. SKID: Kenyon cell skeleton ID. NAME: our assigned name. SIDE: brain hemisphere. VP3, VP2, LACA: Number of inputs from the VP3 vPN, VP2 adPN, and all lACA PNs, within the lACA. LP: Number of inputs from lACA PNs outside of the lACA. VP: Number of inputs from other VP PNs outside of the lACA. OTHER: Other inputs, including from lACA targets (Figure 5).

NEW NAME	PAN	SKID_R	SKID_L	FC MATCH	SCORE	NAMES	DRIVER	SCORE
LHAV3q1#1	5B	2315173	8873592	VGlut-F-200441?	0.643		R64A07	0.677
AV3r1#1	5C	6852116	9018826	VGlut-F-200441?	0.496		R64A07	0.690
DN1a#1	5D	4129363	8867611		NA	DN1a [2]	R43D05 [44]	0.648
PD5g1#1	5H	11210831	8883836	Gad1-F-000010?	0.469			NA
DN1a#2	5D'	3973434	9362353		NA	DN1a [2]	R43D05 [44]	0.650
LHPD5a1#1	5I	5982276	8865547	Gad1-F-000010?	0.487		R91C09?	NA
DN1-l	5E	11524453	8876600		NA		R18H11; R91C09?	0.511
LHPV4c1#1	5F	6292583	8853753	VGlut-F-800074	0.671		R64A07	0.696
LHPV4c1#2	5F'	3968249	8883804	VGlut-F-800074	0.661		R64A07	0.668
PV6r1#1	5K	4214681	8867765		NA		R80C12	0.458
VP2+ adPN	2G	1712057	8864990		NA			NA
VP2 adPN	2O	57516	9159128	Gad1-F-000090	0.615	VP2 uACT1 [3]	R21C11	0.568
LHAV3f1#1	5G	11545803	9711486	VGlut-F-200021	0.681	AV3e1#1 [4]		NA
PV6r1#2		4182361	8870514		NA		R80C12	0.501
AV3r1#2		1309241	9159383		NA		R64A07	0.663
LHPV4c1#3		1120955	8854268	VGlut-F-800074	0.688		R64A07	0.735
VP1m+VP2 lvPN1#3	3F''	57114	13931917		NA		R42H11	0.483
LHAV2d1#1	5J	11543519	2610603		NA	AV2d1#1 [4]		
LHPV4c3#1		5605174	8872558	VGlut-F-800074	0.558		R64A07	0.684
VP1m+ lvPN1	3K	192430	9588978	VGlut-F-700270?	0.579		R31H04?	0.539
PD5g1#2		4294123	8875932	Gad1-F-000010	0.542			NA
LHPV4c4#1		1091854	8880798	VGlut-F-800074	0.593		R64A07	0.683
LHAV4i1#1		3439570		Gad1-F-200137	0.649			NA
PV6r1#3		4182148	8896969		NA		R80C12	0.515
VC5+ adPN*	2N	39254	3420237	Cha-F-200265	0.579	VM6+VP 1 adPN [5]		NA
PPL202#1		30571		TH-F-000011	0.610			NA
LHPV6k2#1		5062598		Gad1-F-400027	0.625		32G09?	0.631
VP1m+VP2 lvPN1#1	3F	57122	9101937		NA		R31H04	0.629
LHPV6f1#3		5824712	8877293		NA		R80C12	0.509
PV6r1#4		11128057			NA		R80C12	0.594
PD5g1#3		9951834	8880131	Gad1-F-000010	0.578			NA
SLP364#1		6292328			NA		R10G01	0.631
LHPV2a1#1		79579		Cha-F-300168	0.697		R37F05	0.615
LHPV6f1#2		8808525			NA		R80C12	0.526
PD5g1#4		8820702		Gad1-F-000010	0.638			NA
LHPV10c1#1	6E	369583			NA			NA
LHAV8a1#1		57110			NA			NA
LHPV6h1#1		9069835			NA			NA
SLP364#2		6292134			NA		R10G01	0.613
VP1m+VP2 lvPN1#2	3F'	57059	12681995		NA		R31H04	0.605
LHPV4h1#1		4044728		Cha-F-500298	NA			NA
LHPV6c1#1			2466931		NA			NA
PV7c1#1			8880705	Cha-F-000074	0.549			NA

Table S2. Table of lACA-associated non-Kenyon cells, related to Figure 5. NEW NAME: our designation, based on neuroblast lineage or lateral horn tract [67]. PAN: Figure panel where example is plotted. SKID_R: skeleton ID of RHS example. SKID_L: skeleton ID of LHS example. FC MATCH: best corresponding single-cell clone in FlyCircuit. SCORE: Average of forward and reverse NBLAST scores for Fly Circuit match. NAMES: Names used in previous publications. DRIVER: best corresponding sparse driver line. SCORE: Forward NBLAST scores for Fly Light match.

TYPE	SKID1	SKID2	SKID3	SKID4
VC5++ l2PN1*	1363077	8825246		
VC5++ l2PN2	8406430			
VP1d il2PN	192423	203504		
VP1d++ vPN	3813442	3813447	4632023	
VP1d+VP1l vPN	3813438			
VP1d+VP4 l2PN1	23005			
VP1d+VP4 l2PN2	3908507			
VP1l+VP3 ilPN	57487	57495		
VP1m l2PN	11524119			
VP1m+ lvPN	192430			
VP1m++ lvPN	57142			
VP1m+VP2 lvPN1	57059	57114	57122	
VP1m+VP2 lvPN2	4058666			
VP1m+VP5 ilPN	46105	57503		
VP2 adPN	57516			
VP2 l2PN	5672990			
VP2+ adPN	1712057			
VP2++ lv2PN	4002166			
VP2+SEZ lvPN1	57146	57166		
VP2+SEZ lvPN2	57063			
VP2+VC5 l2PN	2600341			
VP2+VP5 lvPN	57051	57134	57138	1372988
VP3 vPN	5471515			
VP3+ lPN1	11234968			
VP3+ lPN2	1356477	4869882		
VP3+VP1l ivPN	37513	45882		
VP4 vPN	1149173			
VP4+ vPN	2484510			
VP5 l2PN	4672650			
VP5+ l2PN	10078400			
VP5+SEZ adPN	14003783			
VP5+VP2 l2PN	4876532	5946848		
VP5+VP3 l2PN	4671556			

Table S3. Table of PNs pooled for downstream target analysis, related to Figure 6.
TYPE: our designated morphological type, as in Table 1. SKID: Skeleton ID.

SUPPLEMENTAL REFERENCES

1. Frechter, S., Bates, A.S., Tootoonian, S., Dolan, M.-J., Manton, J., Jamasb, A.R., Kohl, J., Bock, D., and Jefferis, G. (2019). Functional and anatomical specificity in a higher olfactory centre. Elife *8*. Available at: http://dx.doi.org/10.7554/eLife.44590.

2. Shafer, O.T., Helfrich-Förster, C., Renn, S.C.P., and Taghert, P.H. (2006). Reevaluation of Drosophila melanogaster's neuronal circadian pacemakers reveals new neuronal classes. J. Comp. Neurol. *498*, 180–193.

3. Sekiguchi, M., Inoue, K., Yang, T., Luo, D.-G., and Yoshii, T. (2020). A Catalog of GAL4 Drivers for Labeling and Manipulating Circadian Clock Neurons in. J. Biol. Rhythms *35*, 207–213.

4. Stocker, R.F., Lienhard, M.C., Borst, A., and Fischbach, K.F. (1990). Neuronal architecture of the antennal lobe in Drosophila melanogaster. Cell Tissue Res. *262*, 9–34.

5. Huoviala, P., Dolan, M.-J., Love, F.M., Frechter, S., Roberts, R.J.V., Mitrevica, Z., Schlegel, P., Bates, A.S., Aso, Y., Rodrigues, T., *et al.* (2018). Neural circuit basis of aversive odour processing in Drosophila from sensory input to descending output. bioRxiv, 1180. Available at: http://dx.doi.org/10.1101/394403.

6. Yu, H.-H., Kao, C.-F., He, Y., Ding, P., Kao, J.-C., and Lee, T. (2010). A complete developmental sequence of a Drosophila neuronal lineage as revealed by twin-spot MARCM. PLoS Biol. *8*. Available at: http://dx.doi.org/10.1371/journal.pbio.1000461.

7. Bates, A.S., Schlegel, P., Roberts, R.J.V., Drummond, N., Tamimi, I.F.M., Turnbull, R., Zhao, X., Marin, E.C., Popovici, P.D., Dhawan, S., *et al.* (2020). Complete connectomic reconstruction of olfactory projection neurons in the fly brain. bioRxiv, 9. Available at: https://www.biorxiv.org/content/10.1101/2020.01.19.911453v1.

8. Zheng, Z., Lauritzen, J.S., Perlman, E., Robinson, C.G., Nichols, M., Milkie, D., Torrens, O., Price, J., Fisher, C.B., Sharifi, N., *et al.* (2018). A Complete Electron Microscopy Volume of the Brain of Adult Drosophila melanogaster. Cell *174*, 730–743.e22.

9. Zheng, Z., Li, F., Fisher, C., Ali, I.J., Sharifi, N., Calle-Schuler, S., Hsu, J., Masoodpanah, N., Kmecova, L., Kazimiers, T., *et al.* (2020). Structured sampling of olfactory input by the fly mushroom body. bioRxiv, 8393. Available at: https://www.biorxiv.org/content/10.1101/2020.04.17.047167v2.

10. Dolan, M.-J., Frechter, S., Bates, A.S., Dan, C., Huoviala, P., Roberts, R.J., Schlegel, P., Dhawan, S., Tabano, R., Dionne, H., *et al.* (2019). Neurogenetic dissection of the lateral horn reveals major outputs, diverse behavioural functions, and interactions with the mushroom body. Elife *8*. Available at: http://dx.doi.org/10.7554/eLife.43079.
